# Catalytic [2 + 2 + 2] cycloaddition with indium(iii)-activated formaldimines: a practical and selective access to hexahydropyrimidines and 1,3-diamines from alkenes†
†Electronic supplementary information (ESI) available. CCDC 1538722 and 1554489. For ESI and crystallographic data in CIF or other electronic format see DOI: 10.1039/c7sc02576a



**DOI:** 10.1039/c7sc02576a

**Published:** 2017-07-24

**Authors:** Hui Zhou, Hetti Handi Chaminda Lakmal, Jonathan M. Baine, Henry U. Valle, Xue Xu, Xin Cui

**Affiliations:** a Department of Chemistry , Mississippi State University , Mississippi State , MS 39762 , USA . Email: xcui@chemistry.msstate.edu

## Abstract

Catalytic [2 + 2 + 2] cycloaddition with imines has, for the first time, been developed as a practical and selective approach for direct construction of hexahydropyrimidine derivatives from various alkenes. With formaldimines as reagents and simple InCl_3_ as the catalyst, this ionic [2 + 2 + 2] approach is applicable for a wide scope of alkenes and allenes with various electronic and steric properties, as well as substitution patterns. Through facile hydrolysis of the resulting hexahydropyrimidines, this catalytic process also provides a new synthetic strategy for the aminomethylamination of alkenes and allenes to practically access 1,3-diamine derivatives.

## Abstract 2

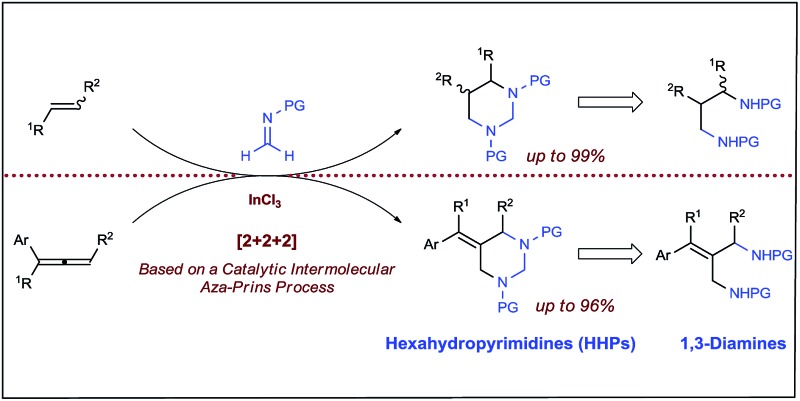
Catalytic [2 + 2 + 2] cycloaddition with three double bond systems has been developed for construction of hexahydropyrimidine and 1,3-diamine derivatives.

## Introduction

**Scheme 1:**
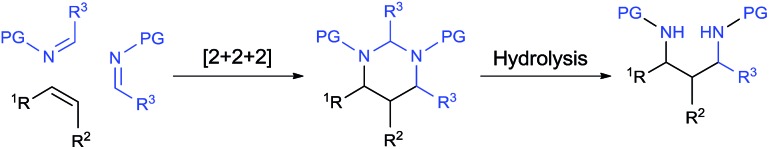
Catalytic [2 + 2 + 2] pathway for transforming alkenes to hexahydropyrimidines and 1,3-diamines.

Catalytic [2 + 2 + 2] cycloaddition reaction represents one of the most efficient approaches for constructing six-membered rings from simple and readily available building blocks.^1^ A number of catalytic systems have been developed to produce aromatic rings from alkynes, and partially saturated cyclic compounds from at least one triple bond system. Cycloaddition that produces saturated cyclohexanes or their heterocyclic analogues from all double bonds, especially simple alkenes, remains as the most challenging process and largely underdeveloped.^1a,1b^ Among different combinations, a well-organized catalytic [2 + 2 + 2] cycloaddition with one molecule of alkene and two molecules of imine would enable direct assembly of hexahydropyrimidines (HHPs) (Scheme 1). As a particularly attractive class of *N*-hetereocycles,^2^ HHPs commonly present as key structural units in numerous natural products,^3^ drugs,^4^ and biologically active molecules.^5^ Besides rich medicinal and biological applications, HHPs have also been developed as polymer stabilizers^6^ and chelating ligands for functional metal complexes.^7^ Synthetically, hydrolysis of the aminal moiety of HHPs would readily produce 1,3-diamine derivatives, another class of molecules that are of synthetic and biological importance.^8^ However, this strategy has never been practically utilized because traditional syntheses of HHPs mainly rely on condensation reactions with presynthesized 1,3-diamines.^2,9^ Development of the aforesaid [2 + 2 + 2] system would provide a direct synthesis of HHPs from alkenes, yet synthetically constitutes a two-step aminoalkyl amination approach for preparing 1,3-diamines from alkenes, a highly applicable but challenging new process that awaits extensive development (Scheme 1).^8^

**Scheme 2:**
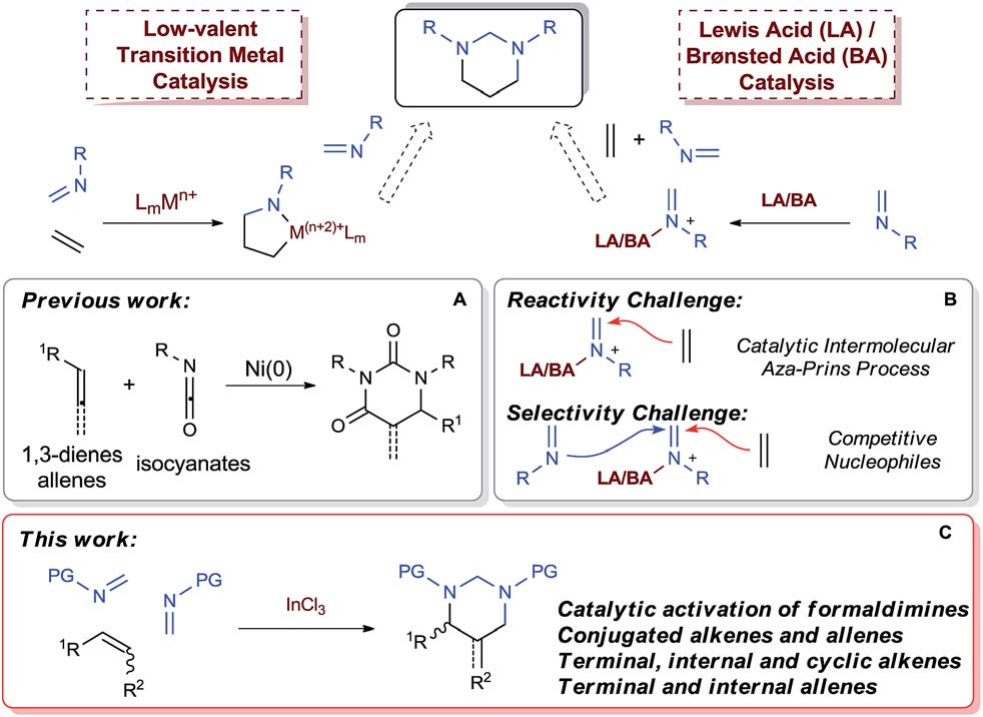
Potential catalytic [2 + 2 + 2] pathways for synthesizing HHPs from alkenes.

Low-valent transition metal-catalyzed [2 + 2 + 2] cycloaddition has emerged as a major tool for the synthesis of carbo- and heterocycles beyond aromatic systems.^1b–f^ New catalytic systems involving Rh^I^,^1b,10^ Ni^0^,^1b,11^ and Co^I^ ^1*b*^ resulted in effective usage of alkenes as cycloaddition partners. While isocyanates have been proven to cyclize with 1,3-dienes or allenes to form dihydropyrimidine-2,4-diones (Scheme 2A),^11c–e^ imines, which bear less electrophilic carbon centers, have not been reported to undergo cyclization with alkenes to form saturated *N*-heterocycles, including HHPs.

Alternatively, acid-catalyzed [2 + 2 + 2] cycloaddition provides an ionic approach for the synthesis of HHPs, although being rarely developed. Recently Sun and coworkers have successfully demonstrated a catalytic [2 + 2 + 2] system with triazines and allenes.^12^ While the Au^I^-catalyst was designed to employ amidoallenes and allenoates for the cycloaddition, a general reactivity toward various alkenes, as well as allenes without activating substituents, remain to be developed. The expected reactivity should require new mechanistic pathways that do not rely on specific electronic properties of the alkene substrates. Among different strategies, a proposed [2 + 2 + 2] process starting with an intermolecular aza-Prins reaction with an acid-complexed imine is particularly attractive as it would be generally applicable for various olefin compounds (Scheme 2). However, although the intramolecular aza-Prins reactions are well studied^13^ and widely utilized in target syntheses,^14^ catalytic intermolecular aza-Prins processes are highly challenging due to the insufficient nucleophilicity of the iminium species.^13d,15^ On the other hand, for a [2 + 2 + 2] cycloaddition toward HHPs, the iminium intermediates are expected to react with one alkene and one imine sequentially. Therefore, selectivity issues brought by alkene and imine as competitive nucleophiles, such as potential imine trimerization or oligomerization,^16^ and iminium-initiated styrene oligomerization, must be addressed, especially for a totally intermolecular version (Scheme 2B).

Herein we wish to report the first catalytic [2 + 2 + 2] system that enables direct construction of HHPs from alkenes and allenes with a wide range of electronic properties and substitution patterns (Scheme 2C). Environmentally benign and inexpensive InCl_3_ (ref. 17) was demonstrated to be a practical and selective catalyst for the three-component cyclization *via* an intermolecular aza-Prins reaction of *N*-sulfonyl formaldimines. Furthermore, the formed HHP derivatives could be readily hydrolyzed to afford various sulfonyl-protected 1,3-diamine derivatives.

## Results and discussion

Initial experiments were performed to identify an effective catalyst for the proposed [2 + 2 + 2] reaction of styrene (**1a**) (Table 1). Compared to many other imines, formaldimines tend to feature thermodynamically and kinetically benefited reactivity as a result of their weaker imine π bond and less steric hindrance. While *N*-aryl and *N*-alkyl formaldimine equivalents have recently been explored as effective aminomethylation reagents,^12,18^ we envisioned *N*-sulfonyl-protected formaldimine would form a more electrophilic iminium with acids to facilitate an intermolecular aza-Prins reaction. Furthermore, the electron-withdrawing sulfonyl group would turn the imine less nucleophilic to suppress its oligomerization.

**Table 1:** Catalytic ionic [2 + 2 + 2] cycloaddition with styrene and *N*-tosyl formaldimine^*a*^

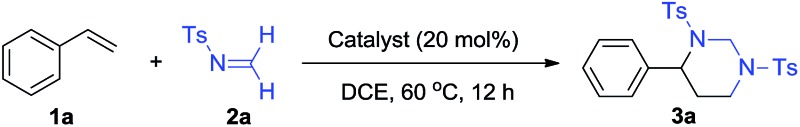					
Entry	Catalyst	Yield (%)^*b*^	Entry	Catalyst	Yield (%)^*b*^
1	—	n.d.^*c*^	13	Cul	n.d.
2	CF_3_CO_2_H	n.d.	14	Cu(OTf)_2_·C_6_H_6_	n.d.
3	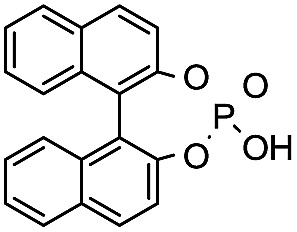	n.d.	15	Zn(OTf)_2_	n.d.
			16	Pd(OAc)_2_	n.d.
			17	RhCl_2_	n.d.
4	BF_3_·Et_2_O	trace	18	RuCl_3_	n.d.
5	B(C_6_F_5_)_3_	trace	19	La(OTf)_3_	13
6	MgCl_2_	n.d.	20	BiCl_3_	49
7	AlCl_3_	n.d.	21	ln(OTf)_3_	37
8	Sc(OTf)_3_	23	**22**	**lnCl**_**3**_	**98**
9	Mn(acac)_3_	n.d.	23^*d*^	lnCl_3_	92
10	FeCl_3_	n.d.	24^*e*^	ln(OTf)_3_	<20
11	CoCl_3_	n.d.	25^*e*^	lnCl_3_	<20
12	NiBr_2_	n.d.	26	lnBr_3_	75

^*a*^Carried out with **1a** (0.1 mmol), **2a** (0.3 mmol), and InCl_3_ (0.02 mmol) in 1.5 mL anhydrous 1,2-dichloroethane (DCE).

^*b*^Isolation yields.

^*c*^Not detected.

^*d*^10 mol% InCl_3_, 60 hours.

^*e*^Reaction carried at room temperature.

Although sulfonyl formaldimine has been shown to react with various strong nucleophiles,^19^ there was no desired reaction observed with styrene under catalyst-free condition at 60 °C (entry 1). Two representative Brønsted acids, trifluoroacetic acid and BINOL-derived phosphoric acid, were tested as catalysts under the same conditions and did not form any desired product (entries 2 and 3). Various Lewis acids, including main group elements, as well as both early and late transition metals with different valences, were then screened with 20 mol% loading at 60 °C (entries 4–21). Moderate to good yields of the desired [2 + 2 + 2] product were observed with several catalysts, including Sc^3+^, Fe^3+^, La^3+^, Bi^3+^ and In^3+^.^20^ While In(OTf)_3_ gave a poorly selective reaction with several side products observed, InCl_3_ was found to catalyze a clean reaction to produce the desired 4-phenyl-1,3-ditosylhexahydropyrimidine (**3a**) in 98% yield (entry 22). Decreasing the catalyst loading to 10 mol% could still afford **3a** in 92% yield albeit with prolonged time (entry 23). Reactions at room temperature were less effective with In^3+^ catalysts (entries 24 and 25). Moreover, InBr_3_ was employed as an alternative catalyst and afforded **3a** in 75% yield (entry 26).

Under the optimized conditions, the InCl_3_-catalyzed [2 + 2 + 2] cycloaddition was evaluated by employing various alkenes (Table 2). Using *N*-tosyl formaldimine **2a**,^21^ electron-rich styrene derivatives bearing *para*- and *meta*-alkyl groups could all be cyclized to form the corresponding HHP products (**3b–3d**). Electron-rich styrene derivatives, such as **3b–3d**, all displayed high reactivity, although slight decrease in selectivity toward HHPs was observed. Considering their increased nucleophilicity that would raise competitive side reactions, such as polymerization, slow addition of these olefins was used and indeed effectively ensured good yields. The structure of **3c** was further confirmed by X-ray analysis on the single crystal. Remarkably, electron-poor 4-trifluoromethylstyrene worked well, affording HHP **3e** in 89% yield at 90 °C. Halogenated styrenes with F, Cl, and Br atoms at different positions, including sterically hindered *ortho*-chlorostyrene and *ortho*-bromostyrene, all served as excellent substrates for the cycloaddition in up to 99% yield (**3f–3j**). In addition to expanded aromatic olefins such as 2-vinylnaphthalene (**3k**), a conjugated diene was shown to produce bicyclic vinyl HHP **3l**, although in decreased yield. Other internal alkenes with both *cis*- and *trans*-configurations are also suitable substrates for synthesizing 2,3-disubstituted HHPs. For example, tricyclic HHP **3m** was synthesized from indene in 60% yield as a single *cis*-diastereomer. Using *cis-β*-methylstyrene and *trans*-stilbene, 2,3-disubstuted HHPs **3n** and **3o** were produced respectively in satisfying yields with excellent *trans*-diastereoselectivity. Finally, *N*-protecting groups beyond tosyl were examined (**2b** and **2c**). HHP **3p** was synthesized in 69% yield with a 2-naphthalenesulfonyl group, which can be deprotected under milder conditions.^22^ Moreover, sterically hindered 2-mesitylenesulfonyl was also well tolerated to form HHP **3q**.

**Table 2:** InCl_3_-catalyzed [2 + 2 + 2] cycloaddition of various olefins with *N*-protected formaldimine^*a*^

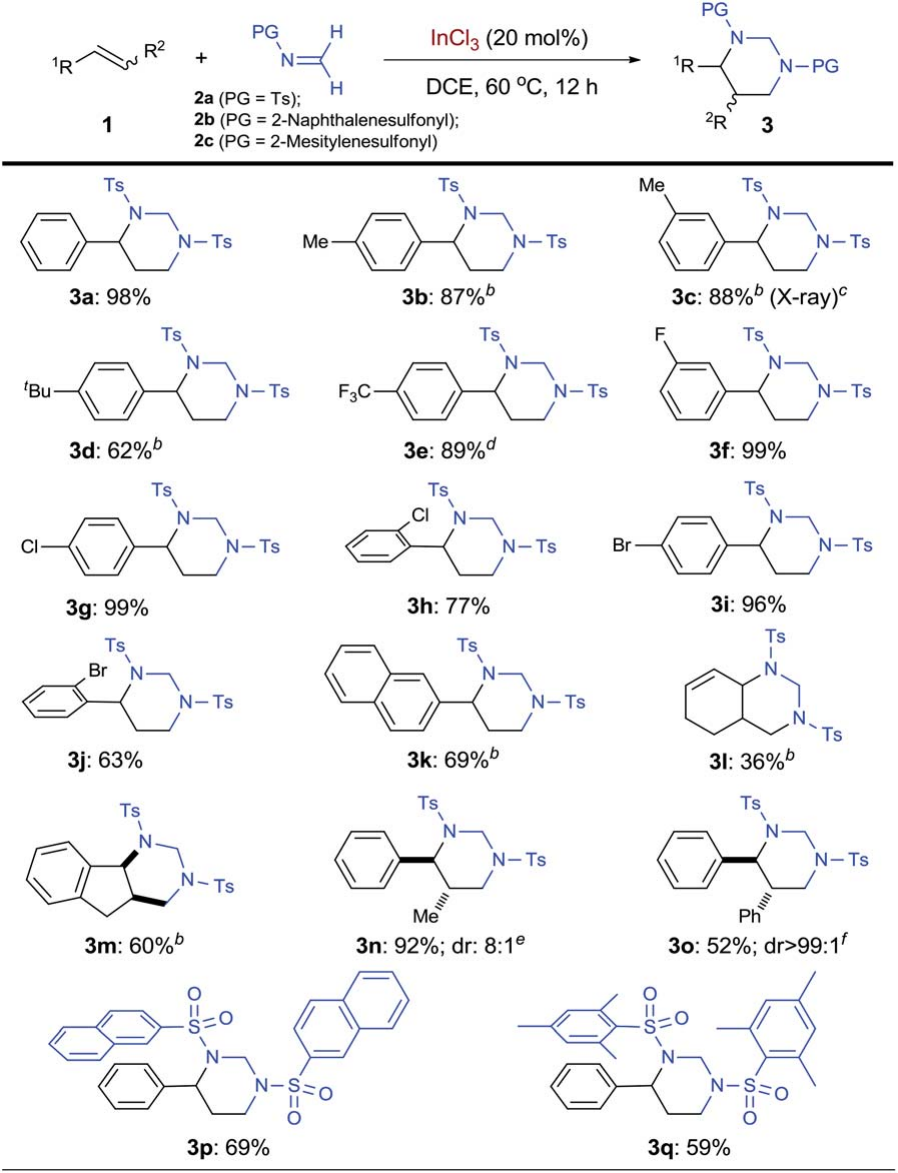

^*a*^Carried out with **1** (0.1 mmol), **2** (0.3 mmol), and InCl_3_ (0.02 mmol) in 1.5 mL anhydrous DCE. Isolation yields are shown.

^*b*^Slow addition of **1** in 3 h followed by stirring for 9 h.

^*c*^The structure was determined by X-ray crystal analysis.

^*d*^90 °C.

^*e*^Starting with *cis-β*-methylstyrene. Diastereomeric ratio was determined by crude HNMR.

^*f*^Starting with *trans*-stilbene.

With the success on alkenes, research efforts were then extended to examine allenes as substrates for the synthesis of vinylidenehexahydropyrimidines, a class of HHP derivatives bearing alkenyl sp^2^ carbon on the ring (Table 3). Interestingly, terminal arylallenes exclusively cyclize with formaldimine **2a** with their terminal double bonds, resulting in the formation of 5-arylidenehexahydropyrimidines (5-AHHPs, **5**). Under the standard conditions, phenylallene and its derivatives with methyl groups at *para*-, *meta*-, and *ortho*-positions all selectively formed 5-AHHPs **5a–5d**, respectively. Halogenated arylallenes with different substitution patterns were all ideal substrates for the [2 + 2 + 2] cycloaddition with 62–96% yield (**5e–5g**). The structure of **5e** was further confirmed by X-ray analysis on the single crystal. Moreover, 1,1-disubstituted allene **4h** was able to form 5-AHHP **5h** bearing a tetrasubstituted olefin unit, albeit in a lower yield. More broadly, an internal allene, 1-methyl-3-phenylallene, was shown to form 5-AHHPs **5i**, with exclusive regioselectivity and 6 : 1 *E*/*Z* ratio.1
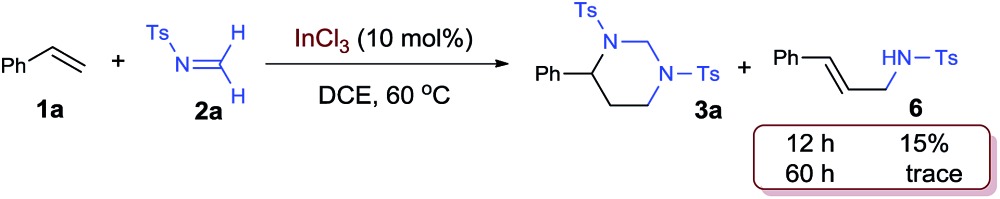

2
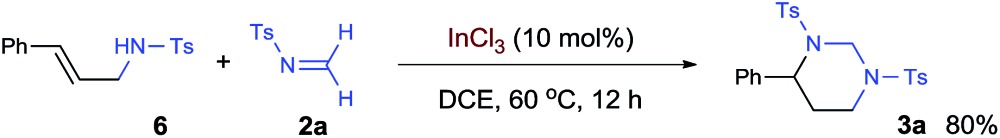



**Table 3:** InCl_3_-catalyzed [2 + 2 + 2] cycloaddition of various allenes with *N*-tosyl formaldimine^*a*^

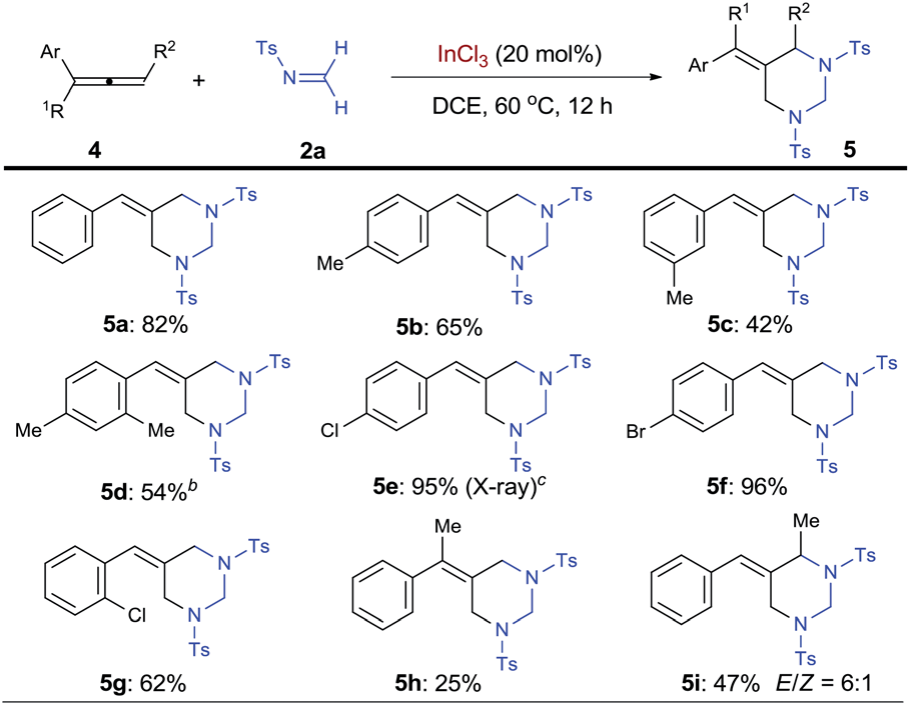

^*a*^Carried out with **4** (0.1 mmol), **2a** (0.3 mmol), and InCl_3_ (0.02 mmol) in 1.5 mL anhydrous DCE. Isolation yields are shown.

^*b*^24 h reaction.

^*c*^The structure was determined by X-ray crystal analysis.

The [2 + 2 + 2] cycloaddition is believed to start with catalytic activation of formaldimine to form an In-complexed iminium species, which was attacked by the alkene and then another imine. The proposed “iminium–alkene–imine” sequence is supported by the observation of an allylamide compound **6** when the reaction was interrupted at an earlier time [eqn (1)]. The styrenyl and *N*-tosyl aminomethyl units in this compound clearly indicate the coupling of one styrene and one imine. While a 15% yield of **6** was observed during a reaction with 10 mol% catalyst, this compound disappeared at the end of the reaction. Furthermore, isolated compound **6** was proven to react with imine **2a** and formed HHP **3a** in a high yield under the standard reaction conditions [eqn (2)].

To shed more light on the proposed aza-Prins step, the diastereoselection of the [2 + 2 + 2] process with internal olefin **1n** was studied as a mechanistic probe [eqn (3)]. Both *cis*- and *trans*-**1n** led to the formation of *trans*-**3n** with the same diastereomeric ratio of 8 : 1, which is consistent with a stepwise mechanism and indicates that aza-Prins step may form a relatively long-lived carbocation before it was quenched by the second formaldimine.3
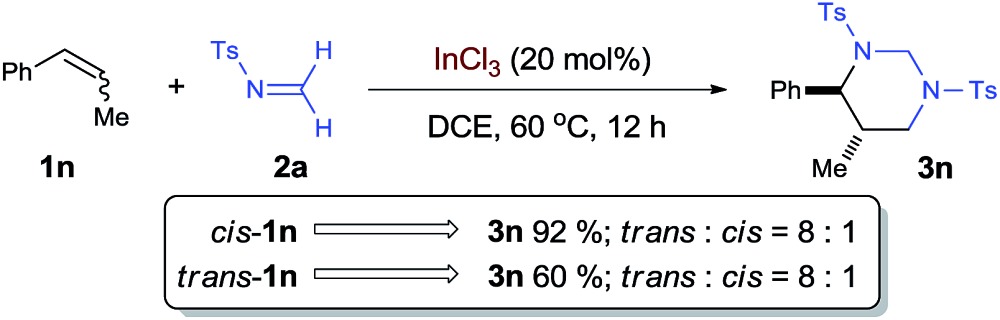



**Scheme 3:**
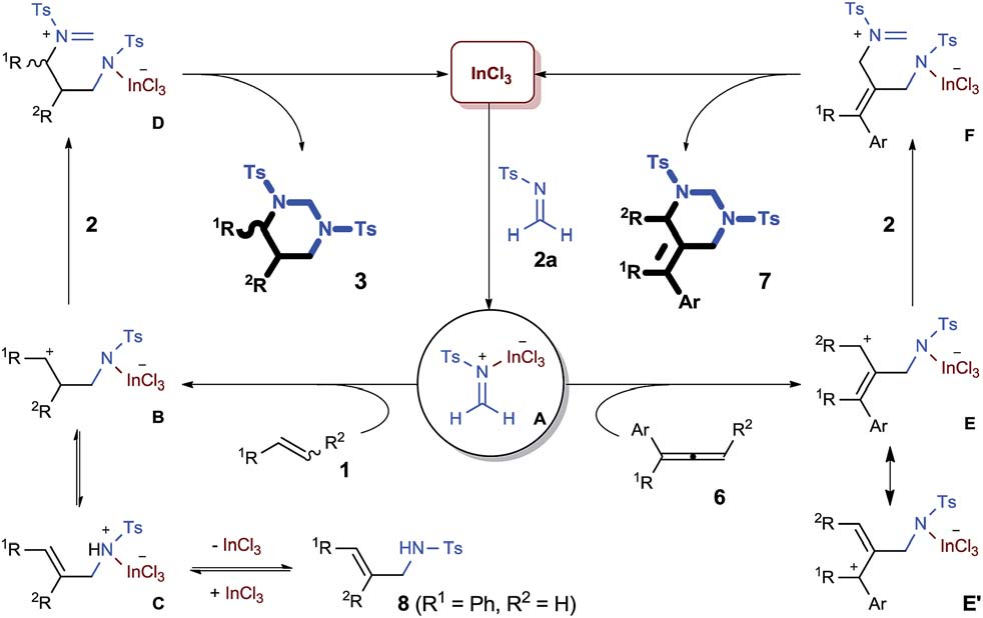
A plausible mechanism for In-catalyzed [2 + 2 + 2] cycloaddition.

A plausible mechanism of this new catalytic [2 + 2 + 2] process is illustrated (Scheme 3). As a key step, highly electrophilic *N*-tosyl formaldiminium **A** initiated an intermolecular aza-Prins reaction to an alkene (**1**) and generate carbocation **B**, which selectively attacked on another imine **2a**, forming formaldiminium **D**. Alternatively, **B** could experience an elimination reaction followed by protonation on the nitrogen center to form allylammonium **C**. The resulting allylamide **8**, which supports the “iminium–alkene–imine” pathway, could reform carbocation **B** through the reversible reactions. Finally a ring closure of intermediate **D** formed the HHP and regenerated the catalyst. The regioselectivity of the cycloaddition with allenes can also be well understood. When iminium **A** selectively attacked the central carbon of the allene unit in **6**, an allyl cation species, which is presented by the two resonance structures **E** and **E′**, was formed. Subsequent electrophilic attack on an imine **2** occurred selectively on the less hindered allyl carbon to yield iminium **F**, which exclusively produced 5-AHHPs (**7**).

**Scheme 4:**
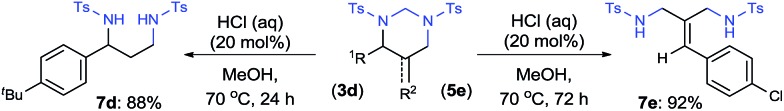
Synthesis of 1,3-diamine derivatives through Hydrolysis of HHPs and 5-AHHPs.

As a synthetic application, both HHP **3d** and 5-AHHP **5e** were readily hydrolyzed with catalytic amount of hydrochloric acid (Scheme 4). Tosyl-protected 1,3-diamines **7d** and **7e** were afforded in 88% and 92% yield, respectively. The simple removal of the aminal units in HHPs offers a practical strategy for the aminomethylamination of both alkenes and allenes.

## Conclusions

In summary, catalytic [2 + 2 + 2] cycloaddition has, for the first time, been proven to cyclize both alkenes and allenes with imines, affording saturated hexahydropyrimidine derivatives. Using environmentally benign catalyst InCl_3_, along with *N*-sulfonyl formaldimines as highly reactive nitrogen source, this process features broad alkene scope, operational simplicity, and exclusive regioselectivity. Mechanistic probing experiments showed consistency with the expected “iminium–alkene–imine” addition pathway, which also result in the exclusive regioselectivity of the cycloaddition with allenes. As a result, a general and practical synthesis of hexahydropyrimidines, as well as 1,3-diamines, from various alkenes and allenes has been established. This catalytic system would encourage further development of catalytic ionic cycloaddition as effective strategy for [2 + 2 + 2] reactions that produce saturated cyclic structures. More broadly, the new catalytic pathway would stimulate further exploration of various tandem reactions that rapidly transform alkenes to highly functionalized molecules.
