# Supplementary material for: Catalytic [2 + 2 + 2] cycloaddition with indium(iii)-activated formaldimines: a practical and selective access to hexahydropyrimidines and 1,3-diamines from alkenes
Source: Chem Sci. 2017 Jul 24;8(9):6520–4. doi: 10.1039/c7sc02576a (PMC5628582; doi:10.1039/c7sc02576a)
Supplement: Supplementary file 1 [file SC-008-C7SC02576A-s001.pdf]

Electronic Supplementary Material (ESI) for Chemical Science.
This journal is © The Royal Society of Chemistry 2017

Supporting Information

Catalytic [2+2+2] Cycloaddition with In(III)-Activated Formaldimines: A General and Selective Access to Hexahydropyrimidines and 1,3-Diamines from Alkenes and Allenes

Hui Zhou, Hetti Handi Chaminda Lakmal, Jonathan M. Baine, Henry U. Valle, Xue Xu, Xin Cui*

Department of Chemistry, Mississippi State University, Mississippi State, Mississippi 39762, United States

Index

General considerations:.. 3

General procedure for the preparation of sulfonyl formaldimines： 3

General procedure for $InCl_3$-catalyzed [2+2+2] cyclization of various alkenes and allenes with *N*-protected formaldimines： 4

Experimental characterization data for hexahydropyrimidines (HHPs)： 5

Experimental characterization data for 5-arylidenehexahydropyrimidines (5-AHHPs)： 13

Isolation of *N*-cinnamyl-4-methylbenzenesulfonamide (**6**) in an interrupted reaction:................ 16

General procedure for the synthesis of 1, 3-diamine derivatives through hydrolysis of HHPs and 5-AHHPs： 17

References:.. 18

^{1}H and ^{13}C NMR spectra for all compounds： 19

X-ray Crystallography 49

General Considerations:

Experimental: Unless otherwise noted, all solvents were dried with sodium benzophenone and distilled before use. All reactions were set up under inert atmosphere (Argon or N_2) utilizing glassware that was flame-dried and cooled under vacuum. All non-aqueous manipulations were using standard Schlenk techniques. Reactions were monitored using thin-layer chromatography (TLC) on Silica Gel plates. Visualization of the developed plates was performed under UV light (254 nm) or $KMnO_4$ stain. Silica-gel flash column chromatography was performed on SYNTHWARE 40-63 μm silica gel.

Materials: Unless otherwise indicated, starting catalysts and materials were obtained from Sigma Aldrich, Oakwood, Strem, or Acros Co. Ltd. Moreover, commercially available reagents were used without additional purification. Imine precursors[1]、tosyl imines[2] and allenes[3-4] with different substituents were prepared according to literature procedures.

Instrumentation: All NMR spectra were run at 300 MHz (^1H NMR) or 500 MHz (^{13}C NMR) in $CDCl_3$ solution. ^1H NMR spectra were internally referenced to TMS. ^{13}C NMR spectra were internally referenced to the residual solvent signal. Data for ^1H NMR are reported as follows: chemical shift (δ ppm), multiplicity (s = singlet, d = doublet, t = triplet, q = quartet, m= multiplet, br = broad), coupling constants (*J*) were reported in Hz. High resolution mass spectra (HRMS) were recorded on Bruker MicrOTOF-QII mass instrument (ESI).

General procedure for the preparation of sulfonyl formaldimines：

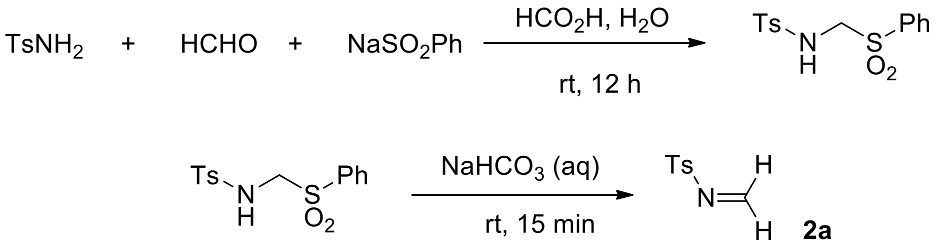

4-methyl-N-(phenylsulfonylmethyl)benzenesulfonamide

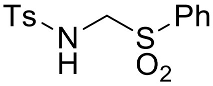

Prepared according to the methods of DolbierJr [1] and Kinoshita[2]. Formaldehyde (1.02 mL, 10.0 mmol, 1.0 equiv) was added to a stirring solution of p-toluenesulfonamide (1.71 g, 10.0 mmol, 1.0 equiv) and sodium phenylsulfinate (1.96 g, 11.0 mmol, 1.1 equiv) in formic acid and water (1:1, 30 mL). After stirring for 12 h at room temperature, the reaction mixture was filtered under reduced pressure and then washed successively with water (50 mL) and hexane (50 mL), after air dry, the desired product was received (3.09 g, 95% yield). **^{1}H NMR** (300 MHz, $CDCl_3$) δ = 7.89 (d, *J* = 8.2 Hz, 2H), 7.72 (t, *J* = 7.2 Hz, 1H), 7.59 (dd, *J* = 13.3, 7.7 Hz, 4H), 7.26 (s, 2H), 5.43 (t, *J* = 6.7 Hz, 1H), 4.38 (d, *J* = 7.1 Hz, 2H), 2.44 (s, 3H); **^{13}C NMR** (126 MHz, $CDCl_3$) δ = 144.30, 136.69, 135.96, 134.54, 129.94, 129.36, 129.14, 126.82, 63.49, 21.58; **HRMS** (ESI) calcd. for $C_{14}H_{15}KNO_4S_2$ [M+K]: 364.0074, found: 364.0075. Observed data was consistent with that reported in the literature. The resulting 4-methyl-N-(phenylsulfonylmethyl)benzenesulfonamide was dissolved in DCE (100 mL) and saturated aqueous sodium bicarbonate solution (100 mL) was added. The resulting biphasic solution was vigorously stirred for 15 mins at rt, after which the organic layer was separated, dried (Na_2SO_4) and the solvent was filtered under reduced pressure to afford the imine/DCE solution (0.2 mol/L, based on the amount of the precursor), and the solution can be stabilized under -20 °C for about 30 days.

General procedure for $InCl_3$-catalyzed [2+2+2] cyclization of various alkenes and allenes with *N*-protected formaldimines:

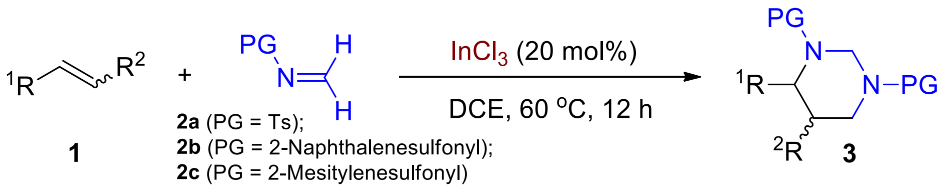

An oven dried Schlenk tube was charged with catalyst $InCl_3$ (20 mol %). The Schlenk tube was then evacuated and back filled with argon. The Teflon screw cap was replaced with a rubber septum and alkenes (**1**, 0.1 mmol) was added followed by sulfonyl imines **2** in dichloroethane solution (0.2 mol/L). The Schlenk tube was then purged with argon for 1 minute and the rubber septum was replaced with a Teflon screw cap. The reaction mixture was then stirred at 60 °C.

After 12 h, the reaction mixture was purified by flash chromatography. For the slow addition protocol: a 10 mL screwtop vial was charged with the following substances: **2** (0.2 mol/L, 1.5 mL, 3 equiv), $InCl_3$ (4.4 mg, 0.02 mmol, 0.2 equiv) and a magnetic stir bar. The vial was placed on a stir plate and stirred vigorously at room temperature while open to ambient atmosphere. After the reaction temperature increased to 60 ^{o}C, a 1.0 mL glass syringe was charged with a solution of alkenes **1** (0.1 mmol, 1 equiv) in dichloroethane (1 mL, 0.1 M) and loaded into a syringe pump set with an addition rate of 0.33 mL/ h (0.0055 mL/min). The syringe was equipped with long needles and directed into the center of the uncapped vial, precautions should be taken not to touch the sides. The addition were initiated simultaneously added to the reaction vial over the course of 3 hours and go on stirring in the oil bath for another 9 hours. The crude mixture was concentrated via rotary evaporation to a minimal amount of dichloroethane and purified by flash chromatography. The fractions containing the product were collected and concentrated by rotary evaporation to afford the compound.

Experimental characterization data for hexahydropyrimidines (HHPs):

4-phenyl-1, 3-ditosylhexahydropyrimidine (3a):

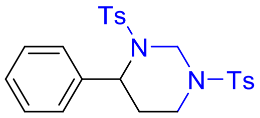

The title compound was prepared according to the general procedure as white solid (46.2 mg, 98% yield, mp: 163.2 -164.7 °C). **^{1}H NMR** (300 MHz, $CDCl_3$) δ = 7.95 (d, *J* = 8.3 Hz, 2H), 7.57 (d, *J* = 8.3 Hz, 2H), 7.40 (d, *J* = 8.1 Hz, 2H), 7.35 – 7.27 (m, 7H), 5.73 (ABq, J_{AB} = 13.1 Hz, 1H), 5.12 (d, *J* = 4.9 Hz, 1H), 3.74 (ABq, J_{AB} = 13.1 Hz, 1H), 3.47 (d, *J* = 12.1 Hz, 1H), 2.56 (dd, *J* = 12.2, 2.2 Hz, 1H), 2.49 (s, 3H), 2.40 (s, 3H), 2.12 (dd, *J* = 14.4, 2.3 Hz, 1H), 1.70 – 1.55 (m, 1H); **^{13}C NMR** (126 MHz, $CDCl_3$) δ = 144.05, 143.97, 136.85, 136.71, 134.19, 129.97, 129.88, 128.91, 127.98, 127.57, 127.20, 126.85, 56.97, 53.43, 41.33, 24.81, 21.70, 21.54; **HRMS** (ESI) calcd. for $C_{24}H_{26}KN_2O_4S_2$ [M+K]: 509.0966, found: 509.0966.

4-p-tolyl-1, 3-ditosylhexahydropyrimidine (3b):

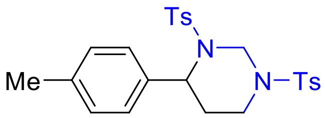

The title compound was prepared according to the general procedure

as white solid (42.2 mg, 87% yield, mp: 143.0 -144.3 °C). **^{1}H NMR** (500 MHz, $CDCl_3$) δ = 7.95 (d, *J* = 8.3 Hz, 2H), 7.57 (d, *J* = 8.2 Hz, 2H), 7.39 (d, *J* = 8.1 Hz, 2H), 7.31 – 7.24 (m, 2H), 7.20 (d, *J* = 8.0 Hz, 2H), 7.13 (d, *J* = 8.1 Hz, 2H), 5.72 (ABq, J_{AB} = 13.1 Hz, 1H), 5.08 (d, *J* = 5.0 Hz, 1H), 3.75 (ABq, J_{AB} = 13.0 Hz, 1H), 3.46 (d, *J* = 12.0 Hz, 1H), 2.56 (td, *J* = 12.2, 2.2 Hz, 1H), 2.49 (s, 3H), 2.41 (s, 3H), 2.32 (s, 3H), 2.14 – 2.05 (m, 1H), 1.65 – 1.55 (m, 1H); **^{13}C NMR** (126 MHz, $CDCl_3$) δ = 143.98, 143.90, 137.33, 136.92, 134.33, 133.62, 129.95, 129.85, 129.58, 127.98, 127.20, 126.79, 56.89, 53.26, 41.32, 29.72, 24.79, 21.69, 21.54, 20.95; **HRMS** (ESI) calcd. for $C_{25}H_{28}N_2NaO_4S_2$ [M+Na]: 507.1383, found: 507.1382.

4-m-tolyl-1, 3-ditosylhexahydropyrimidine (3c):

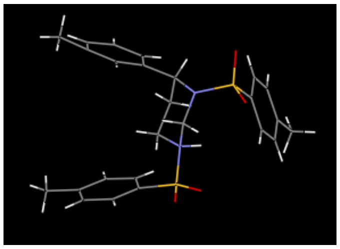

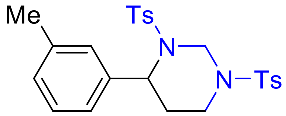

The title compound was prepared according to the general procedure as white solid (42.7 mg, 88% yield, mp: 143.7 -145.1 °C). **^{1}H NMR** (500 MHz, $CDCl_3$) δ = 7.94 (d, *J* = 8.2 Hz, 2H), 7.58 (d, *J* = 8.2 Hz, 2H), 7.39 (d, *J* = 8.1 Hz, 2H), 7.28 (s, 2H), 7.20 (t, *J* = 7.6 Hz, 1H), 7.14 (s, 1H), 7.07 (t, *J* = 6.7 Hz, 2H), 5.73 (ABq, J_{AB} = 13.1 Hz, 1H), 5.08 (d, *J* = 5.0 Hz, 1H), 3.76 (ABq, J_{AB} = 13.1 Hz, 1H), 3.46 (d, *J* = 12.0 Hz, 1H), 2.56 (td, *J* = 12.2, 2.3 Hz, 1H), 2.49 (s, 3H), 2.41 (s, 3H), 2.32 (s, 3H), 2.10 (dd, *J* = 14.3, 2.4 Hz, 1H), 1.59 (s, 1H); **^{13}C NMR** (126 MHz, $CDCl_3$) δ = 144.00, 143.94, 138.72, 136.92, 136.65, 134.34, 129.96, 129.85, 128.71, 128.33, 127.98, 127.61, 127.22, 123.73, 56.99, 53.45, 41.36, 24.88, 21.69, 21.56, 21.54; **HRMS** (ESI) calcd. for $C_{25}H_{28}N_2NaO_4S_2$ [M+Na]: 507.1383, found: 507.1383.

4-(4-tert-butylphenyl)-1, 3-ditosylhexahydropyrimidine (3d):

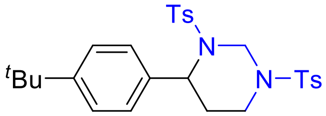

The title compound was prepared according to the general procedure as yellow oil (32.7 mg, 62% yield). **^{1}H NMR** (500 MHz, $CDCl_3$) δ = 7.93 (d, *J* = 8.3 Hz, 2H), 7.60 (d, *J* = 8.3 Hz, 2H), 7.42 – 7.33 (m, 3H), 7.31 (d, *J* = 4.7 Hz, 2H), 7.28 – 7.19 (m, 3H), 5.72 (ABq, J_{AB} = 13.1 Hz, 1H), 5.07 (d, *J* = 4.9 Hz, 1H), 3.84 (ABq, J_{AB} = 13.1 Hz, 1H), 3.44 (s, 1H), 2.63 (d, *J* = 2.3 Hz, 1H), 2.49 (s, 3H),

2.41 (s, 3H), 2.10 (dd, J = 14.4, 2.5 Hz, 1H), 1.63 (d, J = 5.7 Hz, 1H), 1.30 (s, 9H); **^{13}C NMR** (126 MHz, $CDCl_3$) δ = 150.59, 143.92, 143.88, 136.95, 134.58, 133.66, 129.93, 129.84, 127.95, 127.27, 126.55, 125.80, 56.83, 53.34, 41.31, 34.44, 31.27, 25.02, 21.68, 21.53; **HRMS** (ESI) calcd. for $C_{28}H_{35}N_2O_4S_2$ [M+H]: 527.2033, found: 527.2032.

1, 3-ditosyl-4-(4-(trifluoromethyl)phenyl)hexahydropyrimidine (3e):

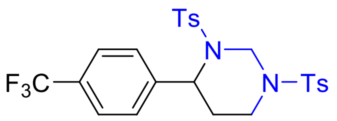

The title compound was prepared according to the general procedure as white solid (48.1 mg, 89% yield, mp: 153.4 -155.0 °C). **^{1}H NMR** (500 MHz, $CDCl_3$) δ = 7.93 (d, J = 8.2 Hz, 2H), 7.58 (d, J = 8.2 Hz, 4H), 7.42 (dd, J = 11.4, 8.5 Hz, 4H), 7.28 (s, 2H), 5.73 (ABq, J_{AB} = 13.0 Hz, 1H), 5.12 (d, J = 4.3 Hz, 1H), 3.72 (ABq, J_{AB} = 13.2 Hz, 1H), 3.48 (d, J = 11.8 Hz, 1H), 2.48 (dd, J = 23.2, 12.8 Hz, 7H), 2.17 – 2.06 (m, 1H), 1.75 – 1.61 (m, 1H); **^{13}C NMR** (126 MHz, $CDCl_3$) δ = 144.34, 144.14, 141.18, 136.49, 134.05, 130.04, 130.01 (q, J = 32.5 Hz), 129.97, 127.96, 127.32, 127.20, 125.86 (q, J = 3.1 Hz), 123.98 (q, J = 271.6 Hz), 57.08, 53.38, 41.24, 25.09, 21.70, 21.56.; **^{19}F NMR** (470 MHz, $CDCl_3$) δ = -63.04 (with PhF as internal standard).; **HRMS** (ESI) calcd. for $C_{25}H_{25}F_3KN_2O_4S_2$ [M+K]: 577.0839, found: 577.0835.

4-(3-fluorophenyl)-1, 3-ditosylhexahydropyrimidine (3f):

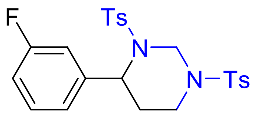

The title compound was prepared according to the general procedure as colorless oil (48.4 mg, 99% yield). **^{1}H NMR** (500 MHz, $CDCl_3$) δ = 7.94 (d, J = 8.2 Hz, 2H), 7.57 (d, J = 8.2 Hz, 2H), 7.40 (d, J = 8.1 Hz, 2H), 7.28 (dd, J = 7.3, 4.9 Hz, 3H), 7.10 (d, J = 7.8 Hz, 1H), 7.02 (d, J = 10.2 Hz, 1H), 6.98 – 6.91 (m, 1H), 5.72 (ABq, J_{AB} = 13.1 Hz, 1H), 5.08 (d, J = 5.0 Hz, 1H), 3.71 (ABq, J_{AB} = 13.1 Hz, 1H), 3.46 (d, J = 12.0 Hz, 1H), 2.55 – 2.45 (m, 4H), 2.41 (s, 3H), 2.11 – 2.01 (m, 1H), 1.63 – 1.57 (m, 1H); **^{13}C NMR** (126 MHz, $CDCl_3$) δ = 163.29 (d, J = 247.2 Hz), 144.17 (d, J = 18.3 Hz), 139.68 (d, J = 6.7 Hz), 136.61, 134.04, 130.49 (d, J = 8.2 Hz), 129.98 (d, J = 10.3 Hz), 127.97, 127.20, 122.41 (d, J = 2.8 Hz), 114.64 (d, J = 21.2 Hz), 114.10 (d, J = 22.8 Hz), 57.04, 53.20, 53.18, 41.27, 24.96, 21.62 (d, J = 17.6 Hz); **^{19}F NMR** (470 MHz, $CDCl_3$) δ = -113.69 (with $PhCF_3$ as internal standard).; **HRMS** (ESI) calcd. for $C_{24}H_{26}FN_2O_4S_2$ [M+H]: 489.1312, found: 489.1312.

4-(4-chlorophenyl)-1, 3-ditosylhexahydropyrimidine (3g):

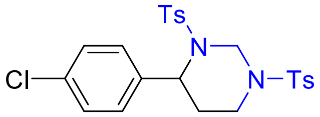

The title compound was prepared according to the general procedure as colorless oil (50.1 mg, 99% yield). **^{1}H NMR** (500 MHz, $CDCl_3$) δ = 7.93 (d, *J* = 8.1 Hz, 2H), 7.56 (d, *J* = 8.1 Hz, 2H), 7.40 (d, *J* = 8.1 Hz, 2H), 7.34 – 7.21 (m, 6H), 5.70 (ABq, J_{AB} = 13.1 Hz, 1H), 5.06 (d, *J* = 4.9 Hz, 1H), 3.66 (ABq, J_{AB} = 13.1 Hz, 1H), 3.46 (d, *J* = 11.9 Hz, 1H), 2.49 (s, 4H), 2.42 (s, 3H), 2.12 – 2.00 (m, 1H), 1.69 – 1.57 (m, 1H); **^{13}C NMR** (126 MHz, $CDCl_3$) δ = 144.23, 144.11, 136.62, 135.35, 133.96, 133.56, 130.03, 129.94, 129.06, 128.36, 127.96, 127.18, 56.96, 53.04, 41.24, 24.76, 21.70, 21.57; **HRMS** (ESI) calcd. for $C_{24}H_{26}ClN_2O_4S_2$ [M+H]: 505.1017, found: 505.1015, [(M+2)+H]: 507.0992. (relative intensity ratio: 3:1)

4-(2-chlorophenyl)-1, 3-ditosylhexahydropyrimidine (3h):

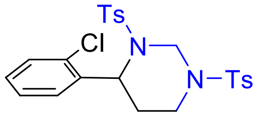

The title compound was prepared according to the general procedure as white solid (39.1 mg, 77% yield, mp: 163.5 -164.1 °C). **^{1}H NMR** (300 MHz, $CDCl_3$) δ = 7.75 (d, *J* = 8.2 Hz, 4H), 7.37 (d, *J* = 8.1 Hz, 2H), 7.30 (dd, *J* = 11.1, 6.6 Hz, 3H), 7.13 (ddt, *J* = 11.7, 7.3, 3.8 Hz, 3H), 5.47 (ABq, J_{AB} = 13.1 Hz, 1H), 4.97 (t, *J* = 6.8 Hz, 1H), 4.79 (ABq, J_{AB} = 13.1 Hz, 1H), 3.16 – 2.92 (m, 2H), 2.47 (d, *J* = 6.9 Hz, 6H), 2.06 – 1.93 (m, 1H), 1.87 – 1.71 (m, 1H); **^{13}C NMR** (126 MHz, $CDCl_3$) δ = 144.15, 144.03, 137.86, 134.92, 134.91, 131.61, 130.01, 129.86, 129.74, 128.70, 127.99, 127.84, 127.50, 126.93, 57.36, 54.02, 41.30, 27.97, 21.64, 21.62; **HRMS** (ESI) calcd. for $C_{24}H_{26}ClN_2O_4S_2$ [M+H]: 505.1017, found: 505.1018, [(M+2)+H]: 507.1297. (relative intensity ratio: 3:1)

4-(4-bromophenyl)-1, 3-ditosylhexahydropyrimidine (3i):

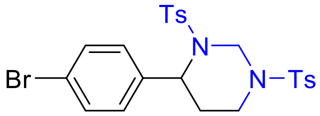

The title compound was prepared according to the general procedure as white solid (52.5 mg, 96% yield, mp: 96.4 -98.0 °C). **^{1}H NMR** (500 MHz, $CDCl_3$) δ = 7.93 (d, *J* = 8.3 Hz, 2H), 7.56 (d, *J* = 8.2 Hz, 2H), 7.43 (d, *J* = 8.5 Hz, 2H), 7.39 (d, *J* = 8.2 Hz, 2H), 7.29 (d, *J* = 4.9 Hz, 2H), 7.19 (d, *J* = 8.4 Hz, 2H), 5.70 (ABq, J_{AB} = 13.1 Hz, 1H), 5.04 (d, *J* = 5.1 Hz, 1H), 3.66 (ABq, J_{AB} = 13.1 Hz, 1H), 3.46 (d, *J* = 12.1 Hz, 1H), 2.49 (s, 3H), 2.46 (dd, *J* = 12.2, 2.3 Hz, 1H), 2.42 (s, 3H), 2.05 (dd, *J* = 13.8, 3.1 Hz, 1H), 1.60 (s, 1H); **^{13}C NMR** (126 MHz, $CDCl_3$) δ = 144.24, 144.12, 136.60, 135.92, 133.95, 132.02, 130.05, 129.94, 128.71, 127.96, 127.17, 121.70, 56.98, 53.10, 41.25,

24.72, 21.71, 21.58; **HRMS** (ESI) calcd. for $C_{24}H_{25}BrN_2NaO_4S_2$ [M+Na]: 571.0331, found: 571.0327, [(M+2)+Na]: 573.0310. (relative intensity ratio: 1:1)

4-(2-bromophenyl)-1,3-ditosylhexahydropyrimidine (3j):

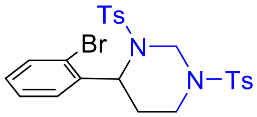

The title compound was prepared according to the general procedure as white solid (34.7 mg, 63% yield, mp: 161.1-163.5 °C). **^{1}H NMR** (300 MHz, $CDCl_3$) δ = 7.75 (dd, *J* = 8.0, 2.2 Hz, 4H), 7.47 (d, *J* = 7.6 Hz, 1H), 7.38 (d, *J* = 7.9 Hz, 2H), 7.32 (d, *J* = 8.0 Hz, 2H), 7.09 (dt, *J* = 12.7, 7.8 Hz, 3H), 5.42 (ABq, J_{AB} = 13.1 Hz, 1H), 4.91 (d, *J* = 6.9 Hz, 1H), 4.85 (ABq, J_{AB} = 13.3 Hz, 1H), 3.13 (dd, *J* = 12.2, 5.6 Hz, 1H), 3.03 – 2.91 (m, 1H), 2.47 (d, *J* = 8.8 Hz, 6H), 2.04 (dd, *J* = 14.5, 4.1 Hz, 1H), 1.84 – 1.65 (m, 1H); **^{13}C NMR** (126 MHz, $CDCl_3$) δ = 144.17, 144.02, 139.60, 134.97, 134.67, 133.07, 130.01, 129.73, 128.98, 128.09, 127.95, 127.54, 127.52, 121.72, 57.30, 56.34, 41.21, 28.19, 21.64, 21.62; **HRMS** (ESI) calcd. for $C_{24}H_{25}BrN_2NaO_4S_2$ [M+Na]: 571.0331, found: 571.0331, [(M+2)+Na]: 573.0310. (relative intensity ratio: 1:1)

4-(naphthalen-2-yl)-1, 3-ditosylhexahydropyrimidine (3k):

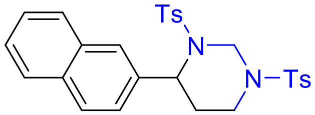

The title compound was prepared according to the general procedure as white solid (35.9 mg, 69% yield, mp: 164.3 -165.2 °C). **^{1}H NMR** (500 MHz, $CDCl_3$) δ = 8.00 (d, *J* = 8.3 Hz, 2H), 7.86 – 7.80 (m, 2H), 7.77 (dd, *J* = 5.9, 3.5 Hz, 1H), 7.67 (s, 1H), 7.58 – 7.47 (m, 5H), 7.41 (d, *J* = 8.1 Hz, 2H), 7.23 (d, *J* = 8.1 Hz, 2H), 5.78 (ABq, J_{AB} = 13.1 Hz, 1H), 5.28 (d, *J* = 5.2 Hz, 1H), 3.75 (ABq, J_{AB} = 13.1 Hz, 1H), 3.58 – 3.49 (m, 1H), 2.59 (td, *J* = 12.3, 2.2 Hz, 1H), 2.50 (s, 3H), 2.35 (s, 3H), 2.27 (dd, *J* = 14.4, 2.2 Hz, 1H), 1.74 – 1.61 (m, 1H); **^{13}C NMR** (126 MHz, $CDCl_3$) δ = 144.14, 144.02, 136.87, 134.16, 134.03, 133.14, 132.62, 130.03, 129.93, 128.91, 128.05, 128.02, 127.56, 127.13, 126.41, 126.39, 125.79, 124.93, 57.18, 53.63, 41.47, 24.69, 21.71, 21.51; **HRMS** (ESI) calcd. for $C_{28}H_{28}KN_2O_4S_2$ [M+K]: 559.1122, found: 559.1120.

1, 3-ditosyl-1, 2, 3, 4, 4a, 5, 6, 8a-octahydroquinazoline (3l):

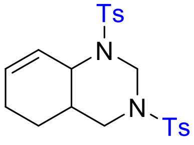

The title compound was prepared according to the general procedure as white solid (16.0 mg, 36% yield, mp: 185.2 -187.6 °C). **^{1}H NMR** (300 MHz, $CDCl_3$) δ = 7.87 (d, *J* = 8.3 Hz, 2H), 7.66 (d, *J* = 8.3 Hz, 2H), 7.35 (dd, *J* = 8.0, 3.6 Hz,

4H), 5.76 (dd, J = 10.2, 2.4 Hz, 1H), 5.68 (ABq, J_{AB} = 12.9 Hz, 1H), 5.32 (d, J = 10.1 Hz, 1H), 4.47 (s, 1H), 3.69 (ABq, J_{AB} = 12.9 Hz, 1H), 3.34 (ddd, J = 11.8, 4.5, 1.8 Hz, 1H), 2.46 (d, J = 1.6 Hz, 7H), 2.00 – 1.66 (m, 3H), 1.63 (d, J = 8.4 Hz, 2H), 1.53 (s, 1H); **^{13}C NMR** (126 MHz, $CDCl_3$) δ = 143.95, 143.81, 137.33, 134.37, 131.38, 129.95, 129.80, 127.77, 127.38, 125.33, 56.24, 52.06, 44.38, 29.25, 23.97, 21.65, 21.60, 20.11; **HRMS** (ESI) calcd. for $C_{22}H_{27}N_2O_4S_2$ [M+H]: 447.1407, found: 447.1407.

1, 3-ditosyl-2, 3, 4, 4a, 5, 9b-hexahydro-1H- indeno[1, 2-d]pyrimidine (3m):

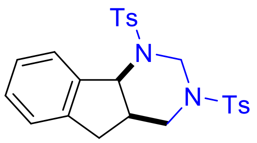

The title compound was prepared according to the general procedure as white solid (29.1 mg, 60% yield, mp: 200.2-202.1 °C). **^{1}H NMR** (300 MHz, $CDCl_3$) δ = 7.99 (d, J = 8.2 Hz, 2H), 7.54 (d, J = 8.2 Hz, 2H), 7.41 (d, J = 8.1 Hz, 2H), 7.37 (d, J = 7.0 Hz, 1H), 7.28 – 7.19 (m, 4H), 7.14 (d, J = 6.9 Hz, 1H), 5.76 (ABq, J_{AB} = 12.9 Hz, 1H), 5.29 (d, J = 6.5 Hz, 1H), 3.57 (ABq, J_{AB} = 13.0 Hz, 1H), 3.48 (ddd, J = 11.8, 6.4, 1.8 Hz, 1H), 2.89 (dd, J = 16.0, 6.3 Hz, 1H), 2.50 (s, 3H), 2.39 (s, 3H), 2.33 (d, J = 16.1 Hz, 1H), 2.27 (dd, J = 11.6, 6.3 Hz, 1H), 1.95 (t, J = 11.7 Hz, 1H); **^{13}C NMR** (126 MHz, $CDCl_3$) δ = 144.05, 144.04, 139.75, 137.88, 136.58, 133.99, 130.01, 129.84, 128.42, 128.12, 127.56, 127.19, 125.54, 124.31, 60.15, 57.13, 46.33, 34.87, 33.83, 21.70, 21.53; **HRMS** (ESI) calcd. for $C_{25}H_{26}KN_2O_4S_2$ [M+K]: 521.0966, found: 521.0965.

***Trans*-5-methyl-4-phenyl-1, 3-ditosylhexahydropyrimidine (3n):**

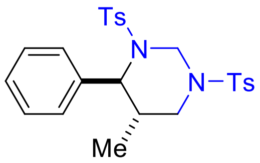

The title compound was prepared according to the general procedure as white solid (44.2 mg, 92% yield, mp: 148.1-148.6 °C). **^{1}H NMR** (500 MHz, $CDCl_3$) δ = 7.69 (d, J = 7.4 Hz, 4H), 7.32 (d, J = 8.1 Hz, 2H), 7.28 (d, J = 3.2 Hz, 3H), 7.26 – 7.20 (m, 2H), 7.17 (d, J = 6.8 Hz, 2H), 5.56 (ABq, J_{AB} = 12.9 Hz, 1H), 4.48 (d, J = 5.3 Hz, 1H), 4.34 (ABq, J_{AB} = 12.9 Hz, 1H), 3.01 (dd, J = 12.0, 3.5 Hz, 1H), 2.95 (dd, J = 12.0, 6.5 Hz, 1H), 2.44 (s, 6H), 2.31 (d, J = 2.4 Hz, 1H), 0.73 (d, J = 6.8 Hz, 3H) ; **^{13}C NMR** (126 MHz, $CDCl_3$) δ = 143.90, 143.65, 138.16, 137.08, 135.01, 129.91, 129.59, 128.64, 127.63, 127.50, 127.37, 127.14, 62.31, 57.11, 47.62, 32.87, 21.59, 21.58, 17.02; **HRMS** (ESI) calcd. for Chemical Formula: $C_{25}H_{28}N_2NaO_4S_2$ [M+Na]: 507.1383, found: 507.1378.

***Trans*-4, 5-diphenyl-1, 3-ditosylhexahydropyrimidine (3o):**

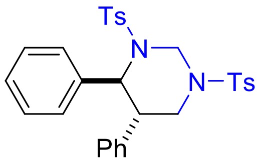

The title compound was prepared according to the general procedure as white solid (28.4 mg, 52% yield, mp: 193.8-196.1 °C). **^{1}H NMR** (300 MHz, $CDCl_3$) δ = 7.77 (d, *J* = 8.1 Hz, 2H), 7.48 (d, *J* = 8.1 Hz, 2H), 7.38 (d, *J* = 8.0 Hz, 2H), 7.24 – 7.04 (m, 8H), 6.93 (d, *J* = 6.8 Hz, 4H), 5.52 (ABq, J_{AB} = 12.9 Hz, 1H), 4.86 (d, *J* = 7.5 Hz, 1H), 4.77 (ABq, J_{AB} = 12.9 Hz, 1H), 3.42 (d, *J* = 8.5 Hz, 1H), 3.23 – 3.09 (m, 2H), 2.49 (s, 3H), 2.42 (s, 3H); **^{13}C NMR** (126 MHz, $CDCl_3$) δ = 144.10, 143.59, 138.68, 138.34, 136.27, 134.99, 129.99, 129.53, 128.76, 128.27, 127.77, 127.66, 127.58, 127.44, 126.94, 63.13, 56.98, 46.97, 45.73, 21.64, 21.58; **HRMS** (ESI) calcd. for Chemical Formula: $C_{30}H_{31}N_2O_4S_2$ [M+H]: 547.1720, found: 547.1721.

1, 3-bis(naphthalen-2-ylsulfonyl)-4-phenylhexahydropyrimidine (3p):

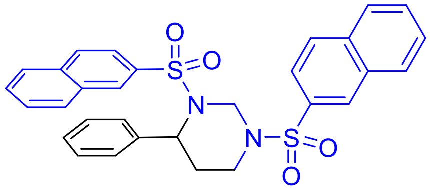

The title compound was prepared according to the general procedure as white solid (37.5 mg, 69% yield, mp: 215.2 - 215.7 °C). **^{1}H NMR** (500 MHz, $CDCl_3$) δ =8.63 (s, 1H), 8.27 (s, 1H), 8.09 (dd, *J* = 8.7, 1.7 Hz, 1H), 8.03 (dd, *J* = 8.0, 6.1 Hz, 2H), 7.95 (d, *J* = 8.2 Hz, 1H), 7.91 (d, *J* = 8.0 Hz, 1H), 7.86 (d, *J* = 8.5 Hz, 2H), 7.68 (t, *J* = 7.0 Hz, 1H), 7.66 – 7.56 (m, 4H), 7.26 – 7.22 (m, 4H), 7.18 (d, *J* = 7.1 Hz, 1H), 5.91 (ABq, J_{AB} = 13.1 Hz, 1H), 5.18 (d, *J* = 5.0 Hz, 1H), 3.87 (ABq, J_{AB} = 13.1 Hz, 1H), 3.46 (d, *J* = 12.2 Hz, 1H), 2.59 (td, *J* = 12.2, 2.2 Hz, 1H), 2.09 – 2.02 (m, 1H), 1.61 – 1.50 (m, 1H); **^{13}C NMR** (126 MHz, $CDCl_3$) δ = 136.70, 136.46, 135.13, 134.94, 134.33, 132.30, 132.22, 129.71, 129.60, 129.45, 129.29, 129.02, 128.96, 128.70, 128.02, 127.96, 127.72, 127.68, 127.58, 126.81, 123.11, 122.13, 57.04, 53.67, 41.38, 25.04; **HRMS** (ESI) calcd. for Chemical Formula: $C_{30}H_{26}KN_2O_4S_2$ [M+K]: 581.0971, found: 581.0971.

1, 3-bis(mesitylsulfonyl)-4-phenylhexahydropyrimidine (3q):

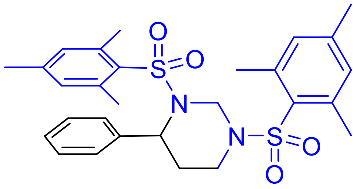

The title compound was prepared according to the general procedure as colorless oil (30.9 mg, 59% yield, mp: 196.5-197.3 °C). **^{1}H NMR** (500 MHz, $CDCl_3$) δ = 7.27 – 7.19 (m, 3H), 7.05 (d, *J* = 7.1 Hz, 2H), 6.98 (s, 2H), 6.91 (s, 2H), 5.22 (ABq, J_{AB} = 12.6 Hz, 1H), 4.87 (t, *J* = 3.9 Hz, 1H), 4.38 (ABq, J_{AB} = 12.7 Hz, 1H), 3.48 – 3.41 (m, 1H), 3.25 (td, *J* = 11.9, 2.9 Hz, 1H), 2.66 (s, 6H), 2.54 (s, 6H), 2.51 (dd, *J* = 8.4, 3.2 Hz, 1H), 2.44 –

2.36 (m, 1H), 2.32 (d, *J* = 17.8 Hz, 6H); **^{13}C NMR** (126 MHz, $CDCl_3$) δ = 143.07, 142.78, 141.08, 139.96, 137.11, 132.73, 132.09, 131.98, 130.79, 128.75, 127.54, 127.05, 54.84, 54.63, 40.47, 26.97, 22.96, 22.74, 21.08, 20.98; **HRMS** (ESI) calcd. for Chemical Formula: $C_{28}H_{35}N_2O_4S_2$ [M+H]: 527.2033, found: 527.2034.

Experimental characterization data for 5-arylidenehexahydropyrimidines (5-AHHPs):

5-benzylidene-1, 3-ditosylhexahydropyrimidine (5a):

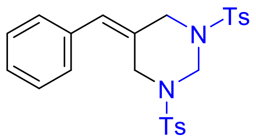

The title compound was prepared according to the general procedure as pare yellow liquid (39.5 mg, 82% yield). **^{1}H NMR** (500 MHz, $CDCl_3$) δ = 7.78 (d, *J* = 8.3 Hz, 2H), 7.53 (d, *J* = 8.3 Hz, 2H), 7.39 – 7.29 (m, 5H), 7.25 (d, *J* = 8.1 Hz, 2H), 6.94 – 6.85 (m, 2H), 6.24 (s, 1H), 4.81 (s, 2H), 3.95 (s, 2H), 3.80 (s, 2H), 2.45 (s, 3H), 2.41 (s, 3H); **^{13}C NMR** (126 MHz, $CDCl_3$) δ = 144.17, 143.92, 134.62, 134.60, 134.57, 129.78, 129.77, 129.68, 128.68, 128.47, 128.17, 127.84, 127.82, 125.26, 61.48, 52.21, 45.80, 21.62, 21.54; **HRMS** (ESI) calcd. for Chemical Formula: $C_{25}H_{27}N_2O_4S_2$ [M+H]: 483.1407, found: 483.1408.

5-(4-methylbenzylidene)-1, 3-ditosylhexahydropyrimidine (5b):

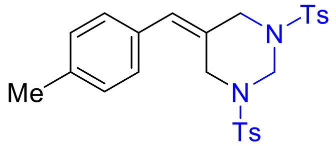

The title compound was prepared according to the general procedure as colorless oil (32.3 mg, 65% yield). **^{1}H NMR** (500 MHz, $CDCl_3$) δ = 7.77 (d, *J* = 8.2 Hz, 2H), 7.54 (d, *J* = 8.2 Hz, 2H), 7.33 (d, *J* = 8.1 Hz, 2H), 7.25 (d, *J* = 8.1 Hz, 2H), 7.16 (d, *J* = 7.9 Hz, 2H), 6.79 (d, *J* = 7.8 Hz, 2H), 6.20 (s, 1H), 4.80 (s, 2H), 3.96 (s, 2H), 3.78 (s, 2H), 2.45 (s, 3H), 2.41 (s, 3H), 2.39 (s, 3H); **^{13}C NMR** (126 MHz, $CDCl_3$) δ = 144.12, 143.87, 137.73, 134.65, 134.54, 131.75, 129.76, 129.64, 129.17, 128.62, 128.14, 127.88, 124.57, 61.48, 52.20, 45.85, 21.62, 21.55, 21.25; **HRMS** (ESI) calcd. for Chemical Formula: $C_{26}H_{28}N_2NaO_4S_2$ [M+Na]: 519.1383, found: 519.1383.

5-(3-methylbenzylidene)-1, 3-ditosylhexahydropyrimidine (5c):

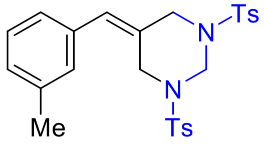

The title compound was prepared according to the general procedure as colorless oil (20.9 mg, 42% yield). **^{1}H NMR** (500 MHz, $CDCl_3$) δ = 7.79 (d, *J* = 8.3 Hz, 2H), 7.54 (d, *J* = 8.3 Hz, 2H), 7.33 (d, *J* = 8.0 Hz, 2H), 7.24 (t, *J* = 7.7 Hz, 3H), 7.12 (d, *J* = 7.6 Hz, 1H), 6.69 (d, *J* = 6.8 Hz, 2H), 6.21 (s, 1H), 4.81 (s, 2H), 3.93 (s, 2H), 3.80 (s, 2H), 2.45 (s, 3H), 2.41 (s, 3H), 2.38 (s, 3H); **^{13}C NMR** (126 MHz, $CDCl_3$) δ = 144.11, 143.88, 138.05, 134.69, 134.58, 129.94, 129.75, 129.66, 129.46, 128.55, 128.34, 128.21, 127.84, 125.69, 125.04, 61.54, 52.25, 45.93, 21.62, 21.54, 21.42; **HRMS** (ESI) calcd. for

Chemical Formula: $C_{26}H_{28}N_2NaO_4S_2$ [M+Na]: 519.1383, found: 519.1382.

5-(2, 4-dimethylbenzylidene)-1, 3-ditosylhexahydropyrimidine (5d):

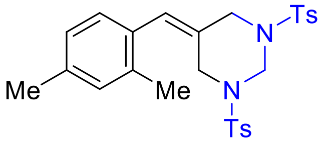

The title compound was prepared according to the general procedure as white solid (27.6 mg, 54% yield, mp: 157.4-159.5 °C). **^{1}H NMR** (500 MHz, $CDCl_3$) δ = 7.81 (d, *J* = 7.8 Hz, 2H), 7.55 (d, *J* = 7.9 Hz, 2H), 7.34 (d, *J* = 7.9 Hz, 2H), 7.25 (s, 2H), 7.00 (s, 1H), 6.94 (d, *J* = 7.8 Hz, 1H), 6.46 (d, *J* = 7.7 Hz, 1H), 6.25 (s, 1H), 4.81 (s, 2H), 3.89 (s, 2H), 3.80 (s, 2H), 2.44 (d, *J* = 3.8 Hz, 6H), 2.34 (s, 3H), 2.02 (s, 3H); **^{13}C NMR** (126 MHz, $CDCl_3$) δ = 144.08, 143.86, 137.87, 136.22, 135.01, 134.45, 130.88, 130.51, 129.79, 129.76, 128.84, 128.57, 128.17, 127.69, 126.42, 125.01, 61.44, 52.09, 46.08, 21.59, 21.57, 21.13, 19.68; **HRMS** (ESI) calcd. for Chemical Formula: $C_{27}H_{31}N_2O_4S_2$ [M+H]: 511.1720, found: 511.1723.

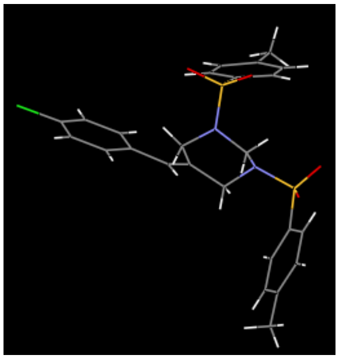

5-(4-chlorobenzylidene)-1, 3-ditosylhexahydropyrimidine (5e):

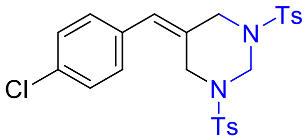

The title compound was prepared according to the general procedure as yellow solid (49.1 mg, 95% yield, mp: 191.7-192.3 °C). **^{1}H NMR** (300 MHz, $CDCl_3$) δ = 7.78 (d, *J* = 8.3 Hz, 2H), 7.54 (d, *J* = 8.3 Hz, 2H), 7.36 – 7.26 (m, 6H), 6.80 (d, *J* = 8.4 Hz, 2H), 6.19 (s, 1H), 4.81 (s, 2H), 3.86 (s, 2H), 3.82 (s, 2H), 2.45 (s, 3H), 2.41 (s, 3H); **^{13}C NMR** (126 MHz, $CDCl_3$) δ = 144.19, 144.07, 134.74, 134.42, 133.77, 133.02, 129.94, 129.74, 128.71, 128.52, 128.23, 127.79, 126.14, 61.48, 52.13, 45.75, 21.62, 21.56; **HRMS** (ESI) calcd. for Chemical Formula: $C_{27}H_{31}N_2O_4S_2$ [M+H]: 517.1017, found: 517.1017, [(M+2)+H]: 519.1008. (relative intensity ratio: 3:1)

5-(4-bromobenzylidene)-1, 3-ditosylhexahydropyrimidine (5f):

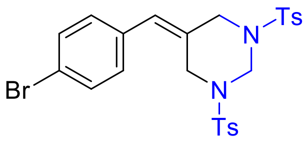

The title compound was prepared according to the general procedure as white solid (54.0 mg, 96% yield, mp: 201.1- 202.9 °C). **^{1}H NMR** (500 MHz, $CDCl_3$) δ = 7.79 (d, *J* = 8.2 Hz, 2H), 7.55 (d, *J* = 8.2 Hz, 2H), 7.48 (d, *J* = 8.4 Hz, 2H), 7.32 (d, *J* = 8.2 Hz, 2H), 7.25 (s, 2H), 6.74 (d, *J* = 8.3 Hz, 2H), 6.17 (s, 1H), 4.81 (s, 2H), 3.86 (s, 2H), 3.82 (s, 2H), 2.45 (s, 3H), 2.41 (s, 3H); **^{13}C NMR** (126 MHz, $CDCl_3$) δ = 144.20, 144.09, 134.74, 134.40, 133.49, 131.65, 130.24, 129.75, 128.55, 128.23, 127.78, 126.20, 121.92, 61.48, 52.13, 45.75, 21.63, 21.57; **HRMS** (ESI) calcd. for Chemical Formula: $C_{25}H_{26}BrN_2O_4S_2$ [M+H]: 561.0512 , found: 561.0508, [(M+2)+H]: 563.0464. (relative intensity ratio: 1:1)

5-(2-chlorobenzylidene)-1, 3-ditosylhexahydropyrimidine (5g):

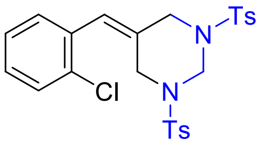

The title compound was prepared according to the general procedure as white solid (32.2 mg, 62% yield, mp: 147.4-149.9 °C). **^{1}H NMR** (300 MHz, $CDCl_3$) δ = 7.81 (d, *J* = 8.3 Hz, 2H), 7.55 (d, *J* = 8.3 Hz, 2H), 7.39 (dd, *J* = 7.9, 1.2 Hz, 1H), 7.32 (d, *J* = 8.0 Hz, 2H), 7.28 (t, *J* = 4.0 Hz, 2H), 7.27 – 7.19 (m, 2H), 6.65 (dd, *J* = 7.5, 1.4 Hz, 1H), 6.33 (s, 1H), 4.82 (s, 2H), 3.93 (s, 2H), 3.78 (s, 2H), 2.45 (s, 3H), 2.40 (s, 3H); **^{13}C NMR** (126 MHz, $CDCl_3$) δ = 144.13, 135.00, 134.12, 133.71, 132.77, 130.46, 129.91, 129.79, 129.59, 129.34, 128.28, 127.59, 126.92, 126.79, 126.64, 61.45, 51.98, 46.11, 21.63, 21.55; **HRMS** (ESI) calcd. for Chemical Formula: $C_{25}H_{25}ClKN_2O_4S_2$ [M+K]: 555.0578 , found: 555.0578, [(M+2)+Na]: 557.0584. (relative intensity ratio: 3:1)

5-(1-phenylethylidene)-1, 3-ditosylhexahydropyrimidine (5h):

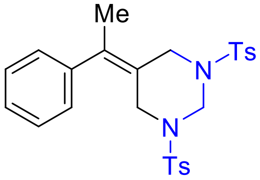

The title compound was prepared according to the general procedure as colorless oil (12.8 mg, 25% yield). **^{1}H NMR** (500 MHz, $CDCl_3$) δ = 7.87 (d, *J* = 8.2 Hz, 2H), 7.52 (d, *J* = 8.2 Hz, 2H), 7.36 (d, *J* = 8.1 Hz, 2H), 7.30 (s, 2H), 7.27 (s, 3H), 6.66 – 6.58 (m, 2H), 4.80 (s, 2H), 4.07 (s, 2H), 3.44 (s, 2H), 2.47 (s, 3H), 2.45 (s, 3H), 1.91 (s, 3H); **^{13}C NMR** (126 MHz, $CDCl_3$) δ = 144.04, 143.93, 140.85, 136.31, 135.96, 133.91, 129.73, 129.56, 128.33, 128.28, 127.63, 127.60, 127.44, 119.58, 61.49, 48.09, 46.88, 21.57, 20.59; **HRMS** (ESI) calcd. for Chemical Formula: $C_{26}H_{28}N_2NaO_4S_2$ [M+Na]: 519.1383 , found: 519.1384.

5-benzylidene-4-methyl-1, 3-ditosylhexahydropyrimidine (5i):

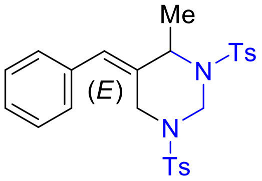

The title compound with an 6:1 *E/Z* ratio was prepared according to the general procedure as brown oil (23.4 mg, 47% yield). **^{1}H NMR** (300 MHz, $CDCl_3$) δ = 7.86 (d, *J* = 8.3 Hz, 2H), 7.56 (d, *J* = 8.3 Hz, 2H), 7.35 – 7.24 (m, 7H), 6.79 (dd, *J* = 7.3, 1.6 Hz, 2H), 6.20 (s, 1H), 5.67 (d, *J* = 13.0 Hz, 1H), 4.54 (d, *J* = 7.1 Hz, 1H), 4.25 (dd, *J* = 13.4, 1.4 Hz, 1H), 4.10 (d, *J* = 13.1 Hz, 1H), 3.15 (d, *J* = 13.4 Hz, 1H), 2.45 (s, 3H), 2.36 (s, 3H), 1.32 (d, *J* = 7.1 Hz, 3H); **^{13}C NMR** (126 MHz, $CDCl_3$) δ = 144.07, 143.86, 136.16, 134.60, 133.70, 129.92, 129.74, 129.50, 128.73, 128.51, 128.38, 127.88, 127.55, 127.49, 55.94, 55.87, 42.15, 21.60, 21.50, 18.64; **HRMS** (ESI) calcd. for Chemical Formula: $C_{26}H_{28}N_2NaO_4S_2$ [M+H]: 497.1563, found: 497.1567.

The *E* configuration of the major isomer was determined by NOESY experiment.

Isolation of *N*-cinnamyl-4-methylbenzenesulfonamide (6) in an interrupted reaction:

An oven dried Schlenk tube was charged with catalyst $InCl_3$ (10 mol %). The Schlenk tube was then evacuated and back filled with argon. The Teflon screw cap was replaced with a rubber septum and alkenes (**1**, 0.1 mmol) was added followed by sulfonyl imines **2** in dichloroethane solution (0.2 mol/L). The Schlenk tube was then purged with argon for 1 minute and the rubber septum was replaced with a Teflon screw cap. The reaction mixture was then stirred at 60 ^{o}C. After 12 h, the reaction mixture was purified by flash chromatography. Then got the desired product with 15% isolated yield.

N-cinnamyl-4-methylbenzenesulfonamide (6):

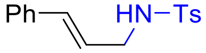

The title compound was prepared according to the method mentioned above as light yellow oil (4.3 mg, 15% yield). **^{1}H NMR** (500 MHz, $CDCl_3$) δ = 7.80 (d, *J* = 8.3 Hz, 2H), 7.33 (d, *J* = 7.7 Hz, 3H), 7.30 (d, *J* = 6.4 Hz, 2H), 7.26 (d, *J* = 7.2 Hz, 2H), 6.46 (d, *J* = 15.8 Hz, 1H), 6.04 (dt, *J* = 15.8, 6.4 Hz, 1H), 4.57 (t, *J* = 6.1 Hz, 1H), 3.78 (td, *J* = 6.3, 1.3 Hz, 2H), 2.44 (s, 3H); **^{13}C NMR** (126 MHz, $CDCl_3$) δ = 143.59, 137.12, 136.09, 133.15, 129.79, 128.59, 127.99, 127.24, 126.43, 124.08, 45.54, 21.53. This compound has previously

been reported[5] and its structure has been confirmed by comparison with the published spectral data.

General procedure for the synthesis of 1, 3-diamine derivatives through hydrolysis of HHPs and 5-AHHPs:

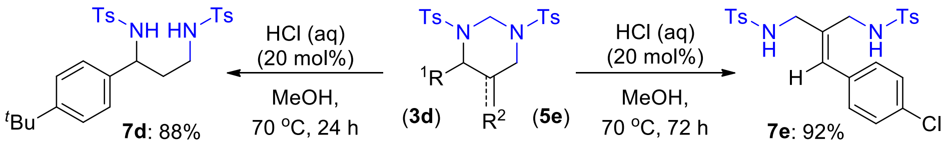

A solution of **3d** or **5e** (0.1 mmol) in 5 mL of methanol was treated with concentrated hydrochloric acid (20 mol%). The mixture was stirred for indicated time under gentle reflux until completion of the reactions. After cooling to room temperature, the solution was treated with NaOH solution (4 M, 5 mL, 20.0 mmol) and the resulting mixture was extracted with ethyl acetate (5 mL × 4). The combined organic layer was dried over Na_2SO_4, filtered, and concentrated to give 1, 3-diamines **7**.

N, N'-(1-(4-tert-butylphenyl)propane-1, 3-diyl)bis(4-methylbenzenesulfonamide) (7d):

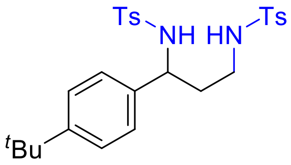

The title compound was prepared according to the general procedure as white solid (45.3 mg, 88% yield, mp: 144.0-146.7 °C). **^{1}H NMR** (300 MHz, $CDCl_3$) δ = 7.77 (d, *J* = 7.7 Hz, 2H), 7.51 (d, *J* = 7.9 Hz, 2H), 7.32 (d, *J* = 7.7 Hz, 2H), 7.10 (d, *J* = 7.6 Hz, 4H), 6.75 (d, *J* = 7.7 Hz, 2H), 5.16 (d, *J* = 6.1 Hz, 1H), 5.09 (d, *J* = 8.9 Hz, 1H), 4.30 (d, *J* = 7.7 Hz, 1H), 3.10 (d, *J* = 5.9 Hz, 2H), 2.45 (s, 3H), 2.36 (s, 3H), 1.95 (d, *J* = 6.3 Hz, 2H), 1.25 (s, 9H); **^{13}C NMR** (126 MHz, $CDCl_3$) δ = 150.69, 143.36, 143.14, 137.06, 136.75, 129.76, 129.34, 127.13, 127.11, 125.80, 125.42, 55.37, 39.91, 37.34, 34.41, 31.26, 21.54, 21.48; **HRMS** (ESI) calcd. for Chemical Formula: $C_{26}H_{28}N_2NaO_4S_2$ [M+Na]: 537.1852, found: 537.1850.

N, N'-(2-(4-chlorobenzylidene)propane-1,3-diyl)bis(4-methylbenzenesulfonamide) (7e)

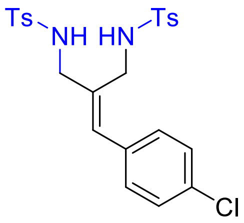

The title compound was prepared according to the general procedure as light yellow solid (46.5 mg, 92% yield, mp: 149.6-153.9 °C). **^{1}H NMR** (300 MHz, $CDCl_3$) δ = 7.77 (d, *J* = 8.0 Hz, 2H), 7.63 (d, *J* = 8.0 Hz, 2H), 7.32 (d, *J* = 8.0 Hz, 2H), 7.29 (s, 2H), 7.24 (d, *J* = 8.5 Hz, 2H), 6.96 (d, *J* = 8.2 Hz, 2H), 6.50 (s, 1H), 5.14 (t, *J* = 6.6 Hz, 1H), 5.07 (t, *J* = 6.3 Hz, 1H), 3.74 (d, *J* = 6.6 Hz, 2H), 3.63 (d, *J* = 6.3 Hz, 2H), 2.45 (s, 6H); **^{13}C NMR** (126 MHz, $CDCl_3$) δ = 143.81, 143.74, 136.90, 136.05, 133.73, 133.48, 131.00, 129.96, 129.85, 129.79, 128.55, 127.18, 127.16, 47.58, 40.81, 21.56; **HRMS** (ESI) calcd. for Chemical Formula: $C_{24}H_{25}ClN_2NaO_4S_2$ [M+Na]: 527.0836, found: 527.0833, [(M+2)+Na]: 529.0807. (relative intensity ratio: 3:1)

References:

1. Zhang, Z. X.; Tang, X. J.; Thomoson, C. S.; DolbierJr, W. R. *Org. Lett*. **2015**, *17*(14), 3528-3531.
2. Kinoshita, H.; Inomata, K.; Hayashi, M.; Kondoh, T.; Kotake, H. *Chem. Lett.* **1986,** *15*, 1033-1036.
3. Matsubara, T.; Takahashi, K.; Ishihara, J.; Hatakeyama, S. *Angew. Chem. Int. Ed.* **2014**, *53*(3), 757-760.
4. Rigby, J. H.; Laurent, S. B.; Kamal, Z.; Heeg, M. J. *Org. let*. **2008**, *10*(24), 5609-5612.
5. Ghorai, M. K.; Kumar, A.; Das, K. *Org. lett*. **2007**, *9*(26), 5441-5444.

^{1}H and ^{13}C NMR spectra for all compounds:

4-phenyl-1, 3-ditosylhexahydropyrimidine (3a):

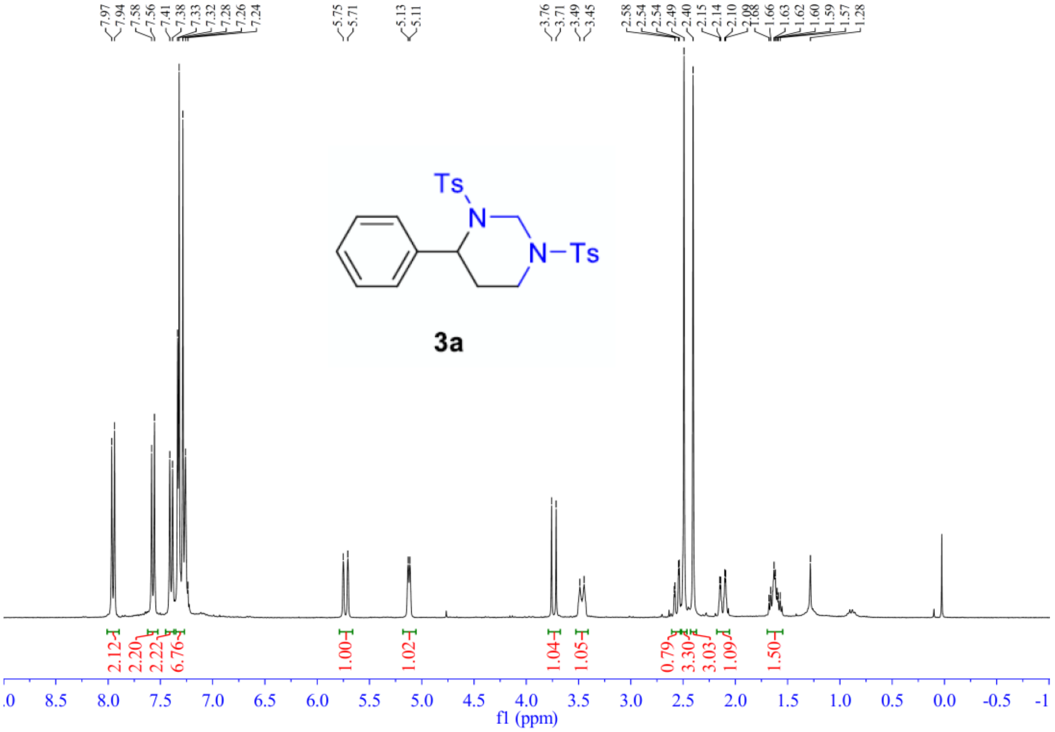

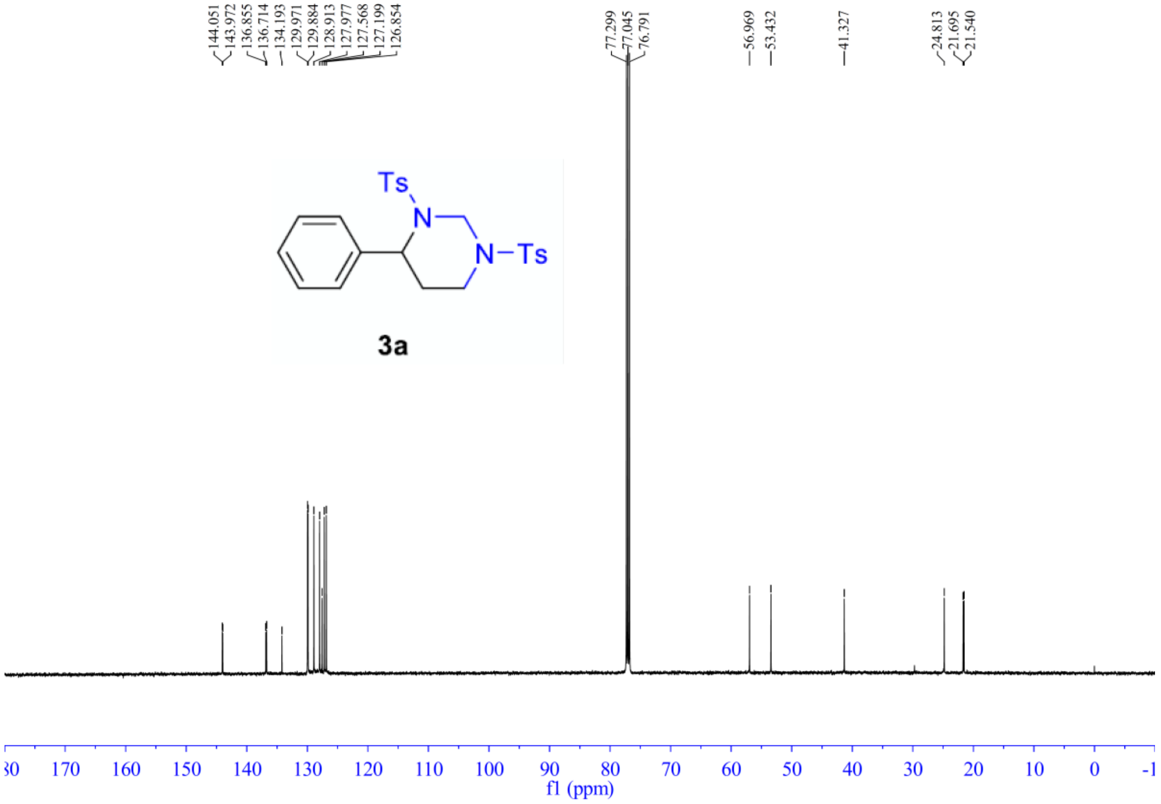

4-p-tolyl-1, 3-ditosylhexahydropyrimidine (3b):

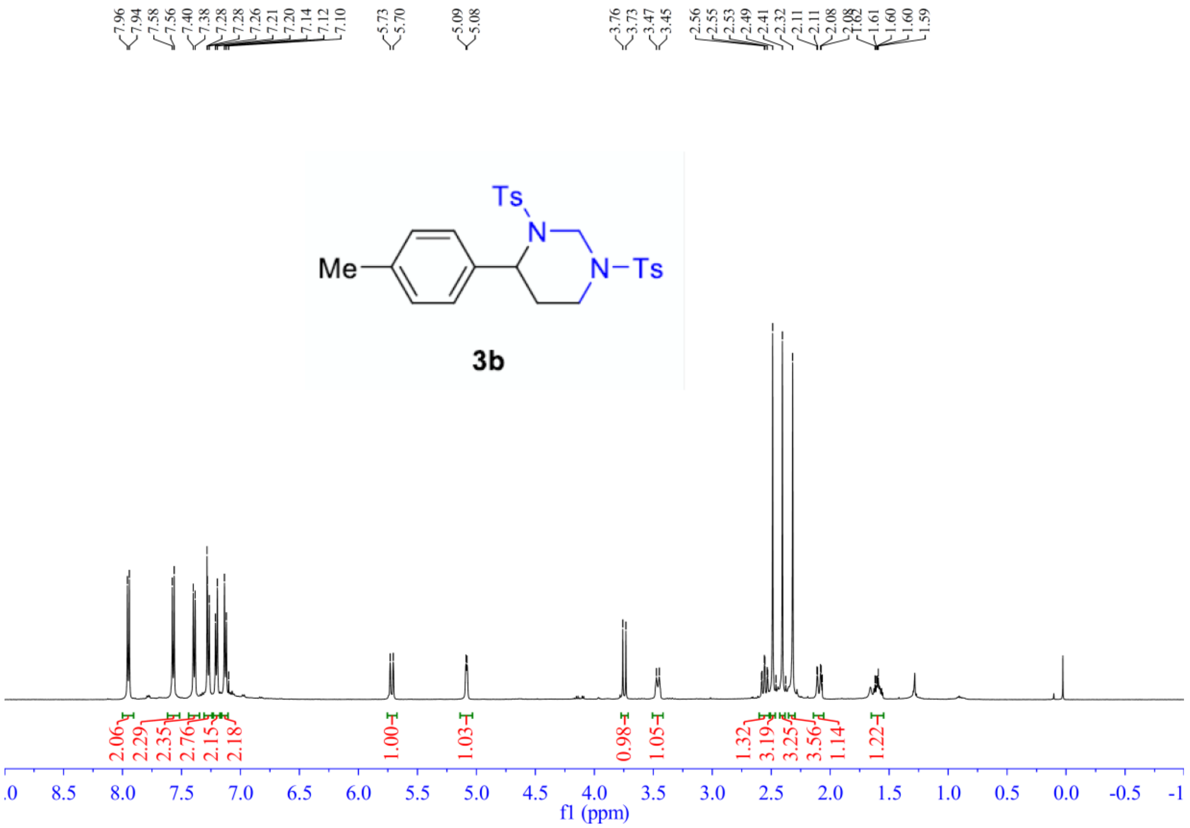

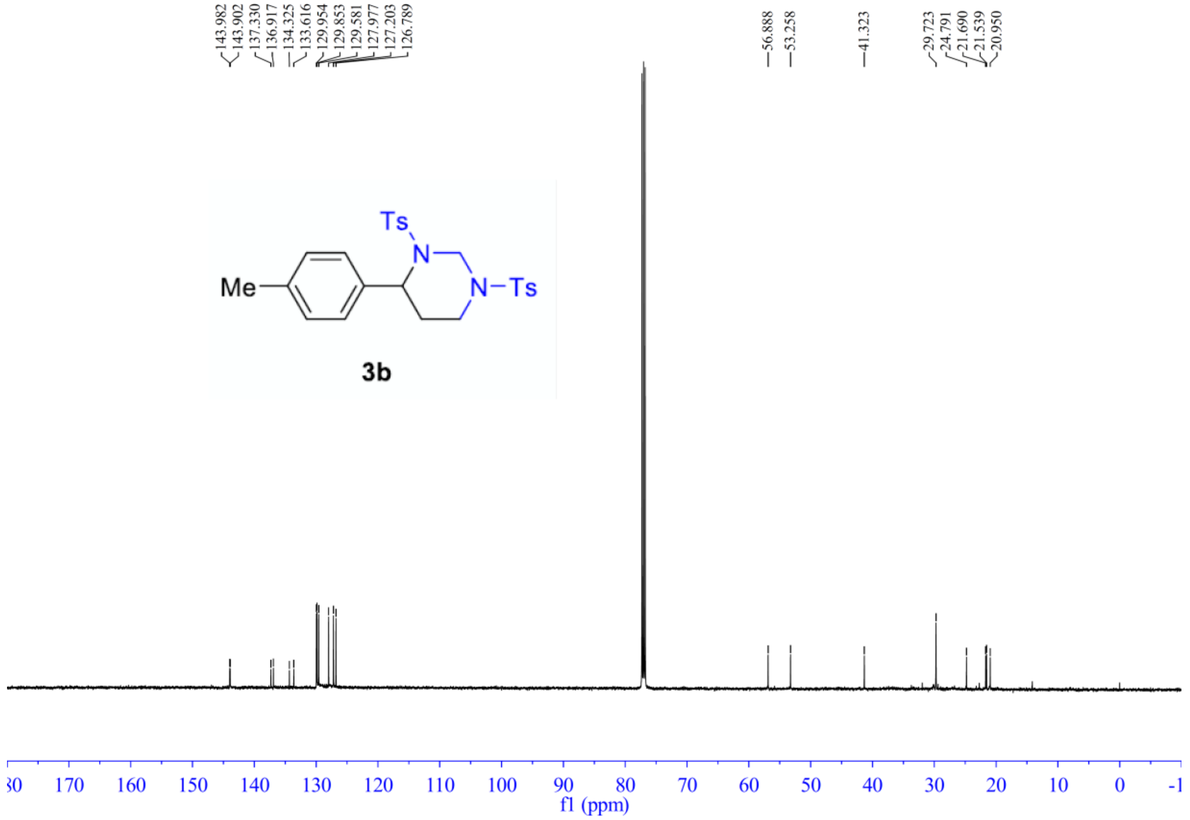

4-m-tolyl-1, 3-ditosylhexahydropyrimidine (3c)：

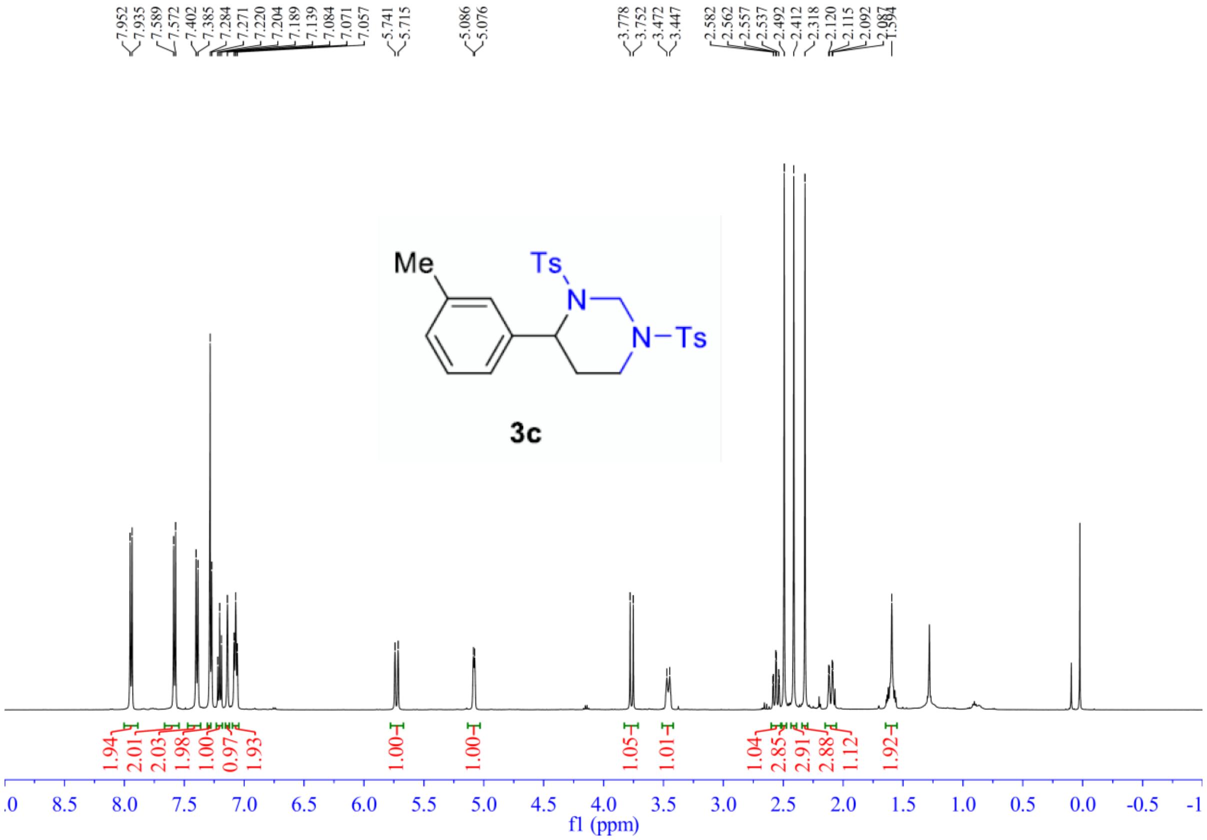

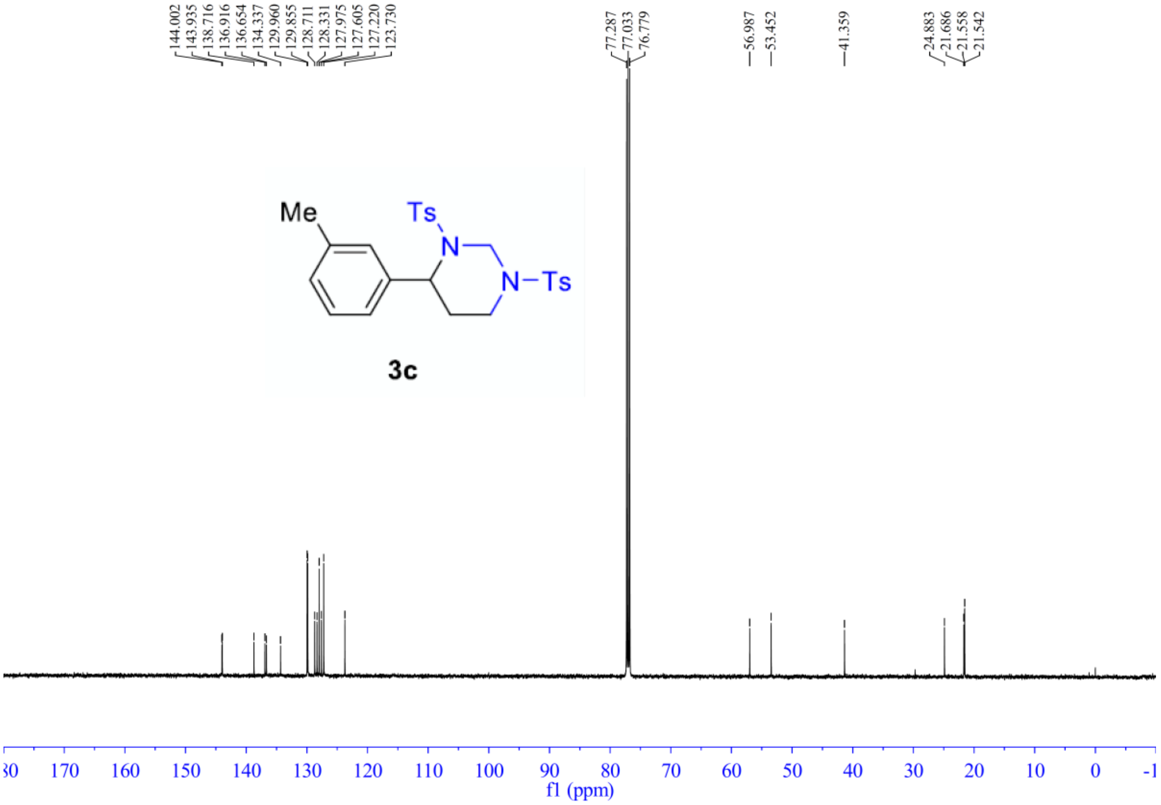

4-(4-tert-butylphenyl)-1, 3-ditosylhexahydropyrimidine (3d):

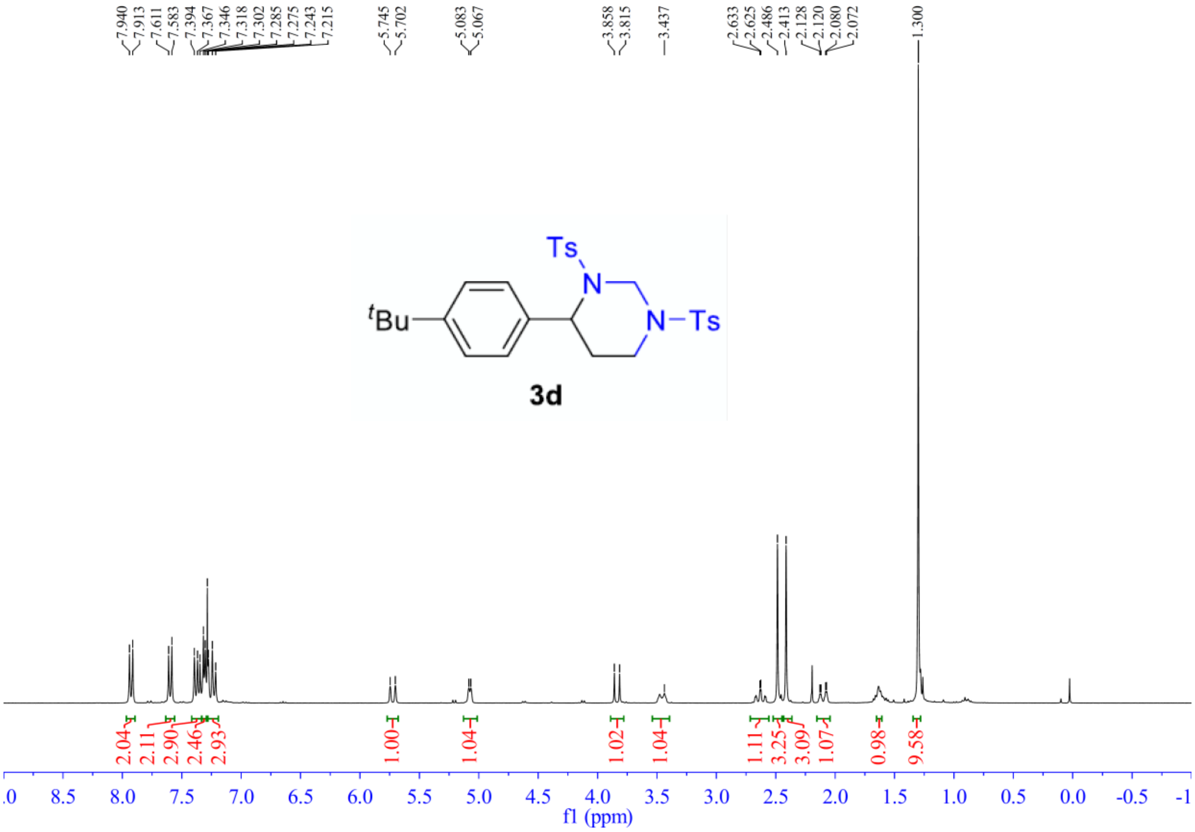

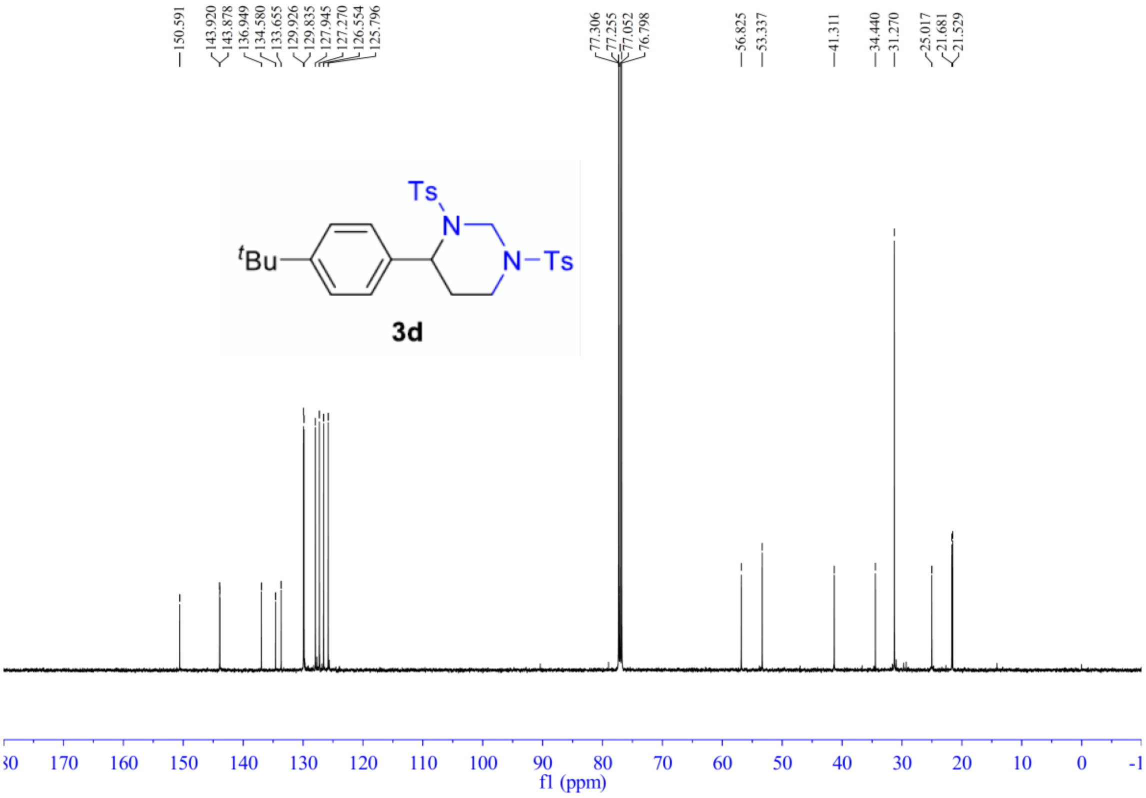

1, 3-ditosyl-4-(4-(trifluoromethyl)phenyl)hexahydropyrimidine (3e):

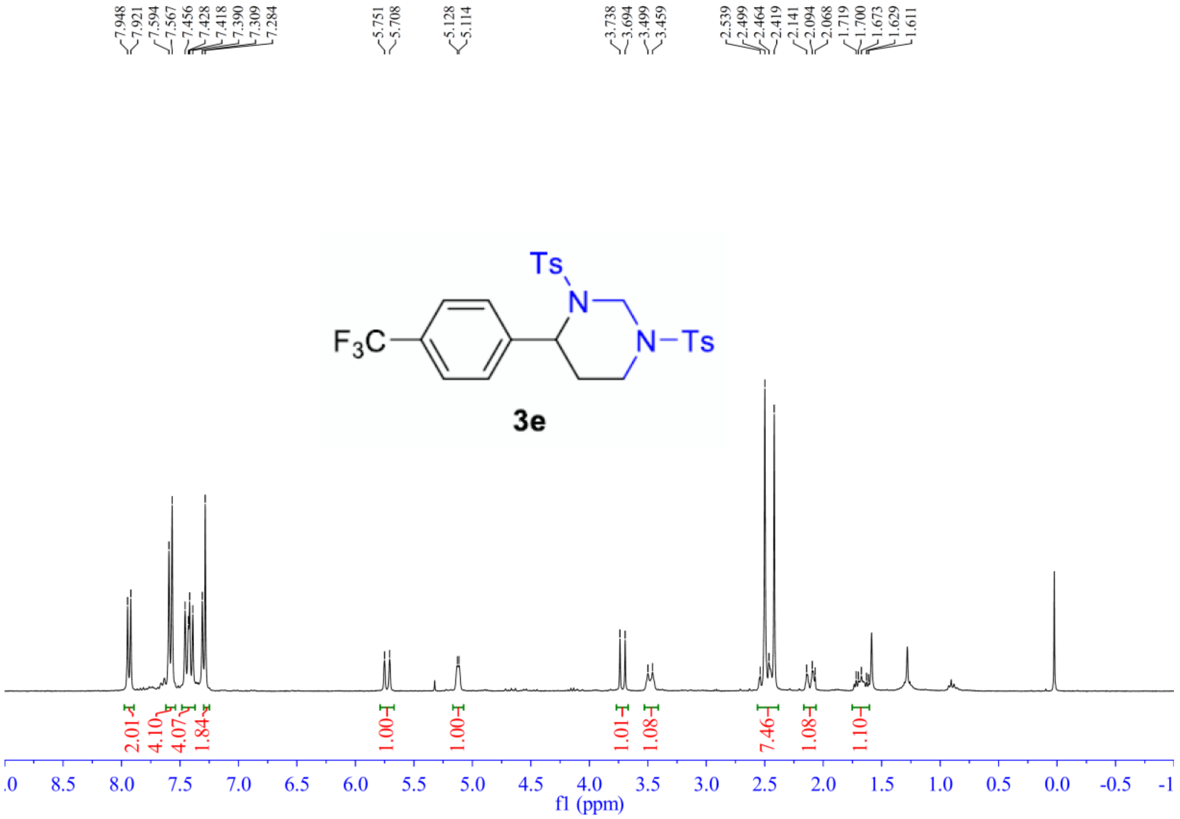

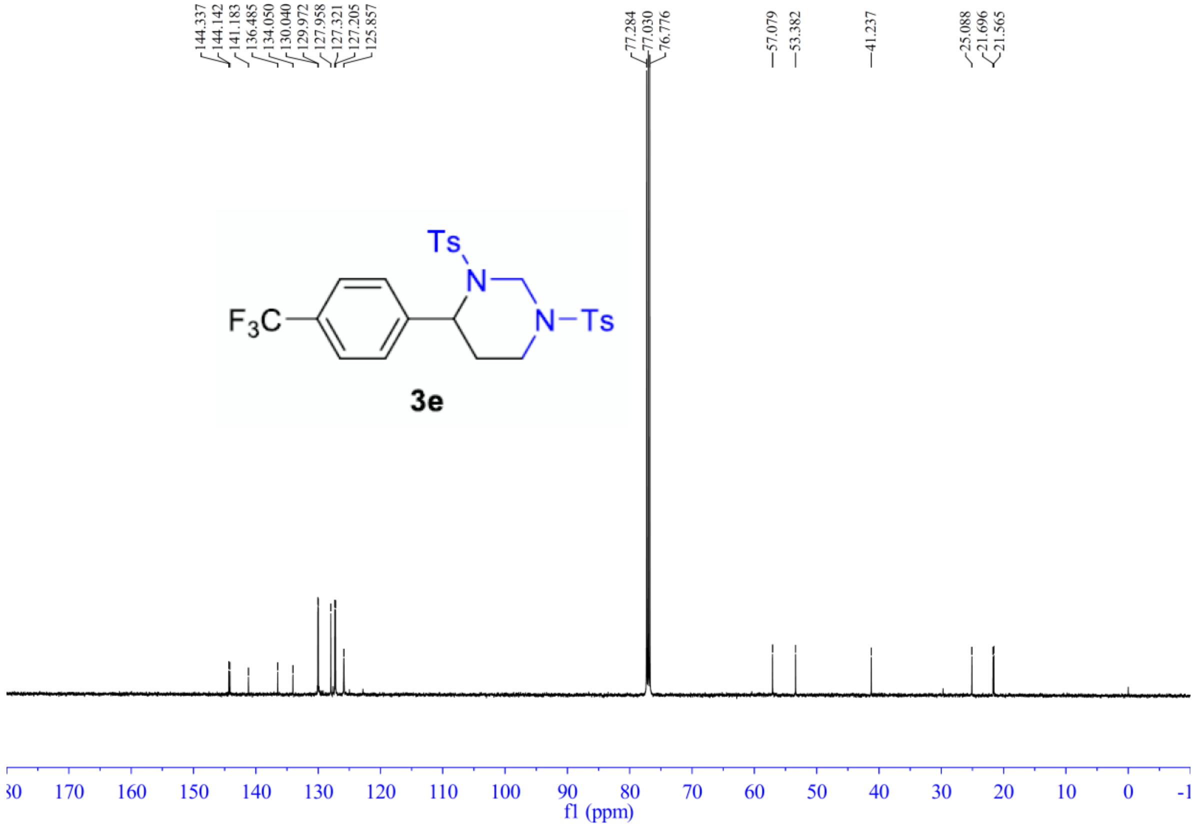

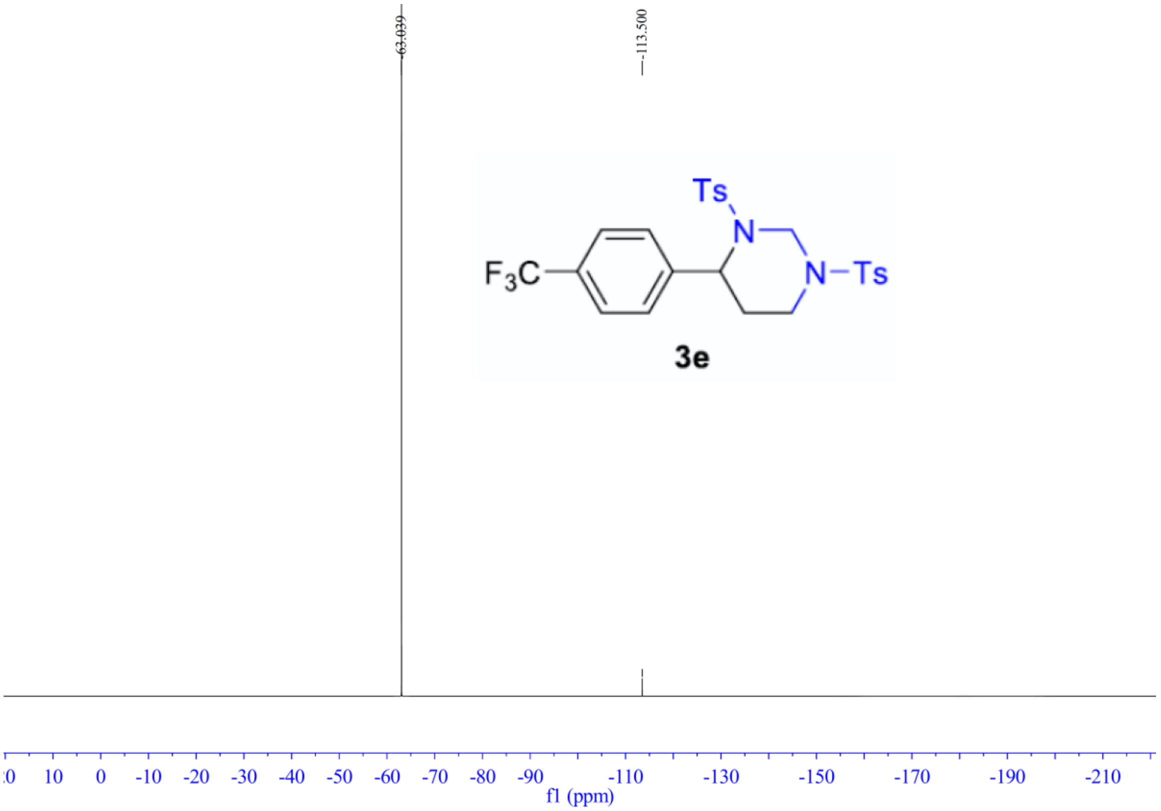

4-(3-fluorophenyl)-1, 3-ditosylhexahydropyrimidine (3f):

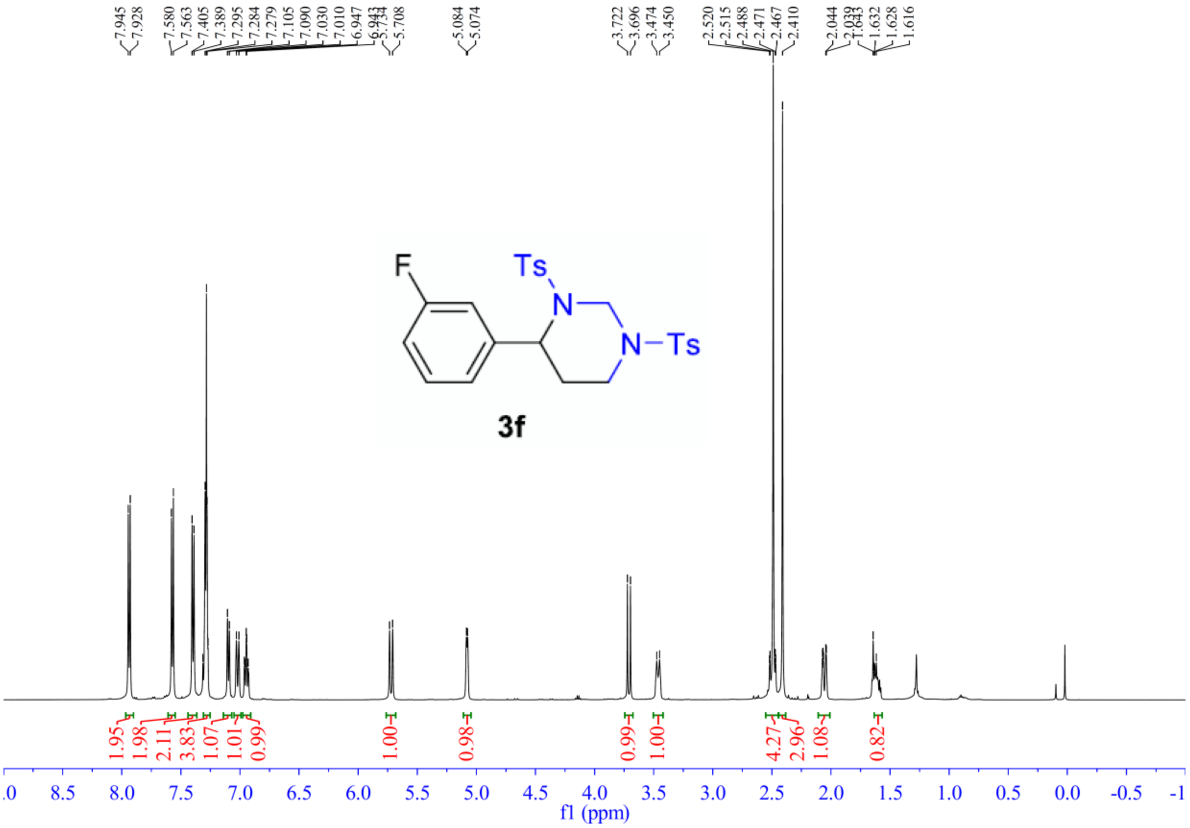

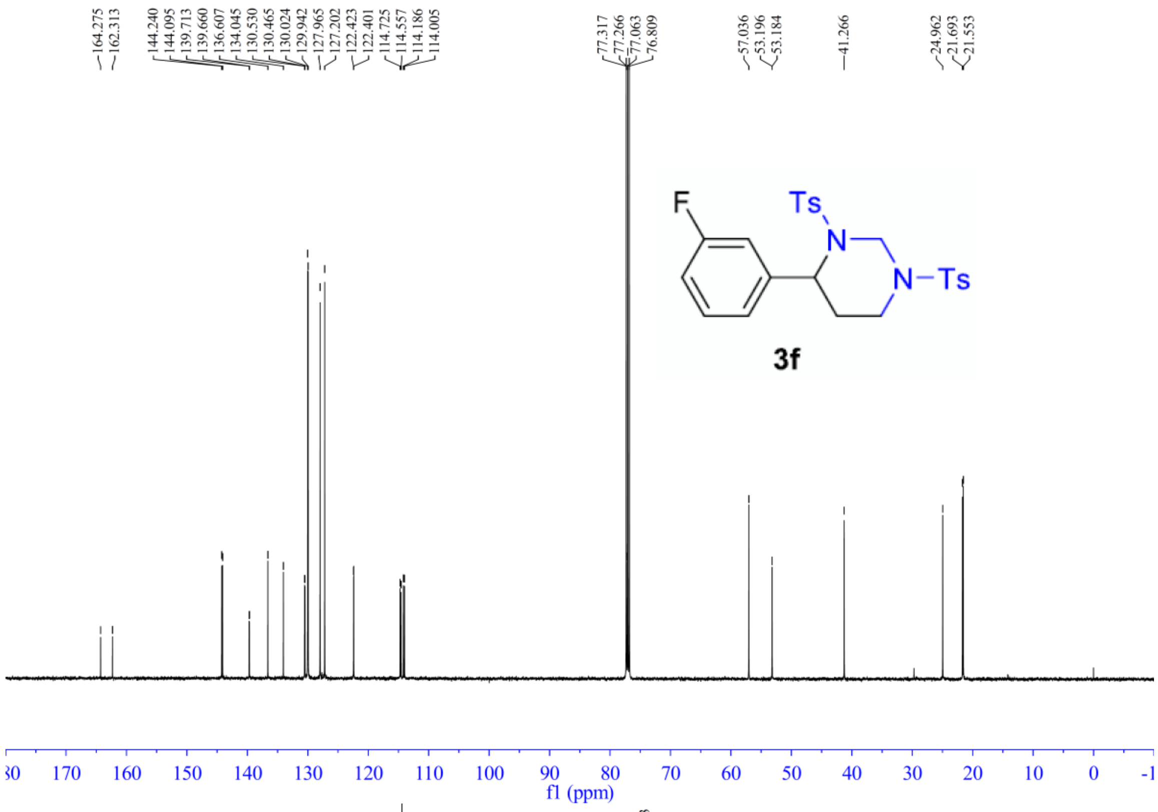
164.275
162.313
144.240
144.095
139.713
139.660
136.607
134.045
130.530
130.465
130.024
129.942
127.965
127.202
122.423
122.401
114.725
114.557
114.186
114.005
77.317
77.266
77.063
76.809
57.036
53.196
53.184
41.266
24.962
21.693
21.553
F
Ts
N
N−Ts
3f
170 160 150 140 130 120 110 100 90 80 70 60 50 40 30 20 10 0
f1 (ppm)

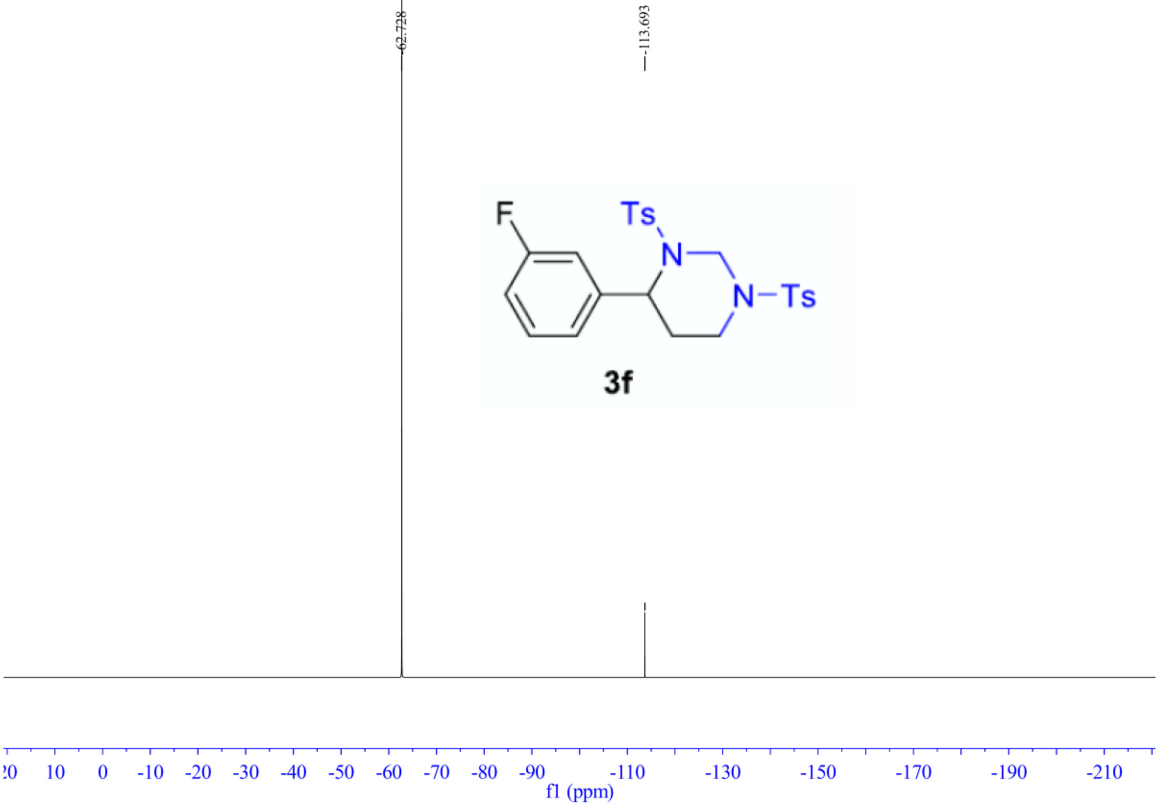
-62.728
-113.693
F
Ts
N
N−Ts
3f
10 0 -10 -20 -30 -40 -50 -60 -70 -80 -90 -110 -130 -150 -170 -190 -210
f1 (ppm)

4-(4-chlorophenyl)-1, 3-ditosylhexahydropyrimidine (3g):

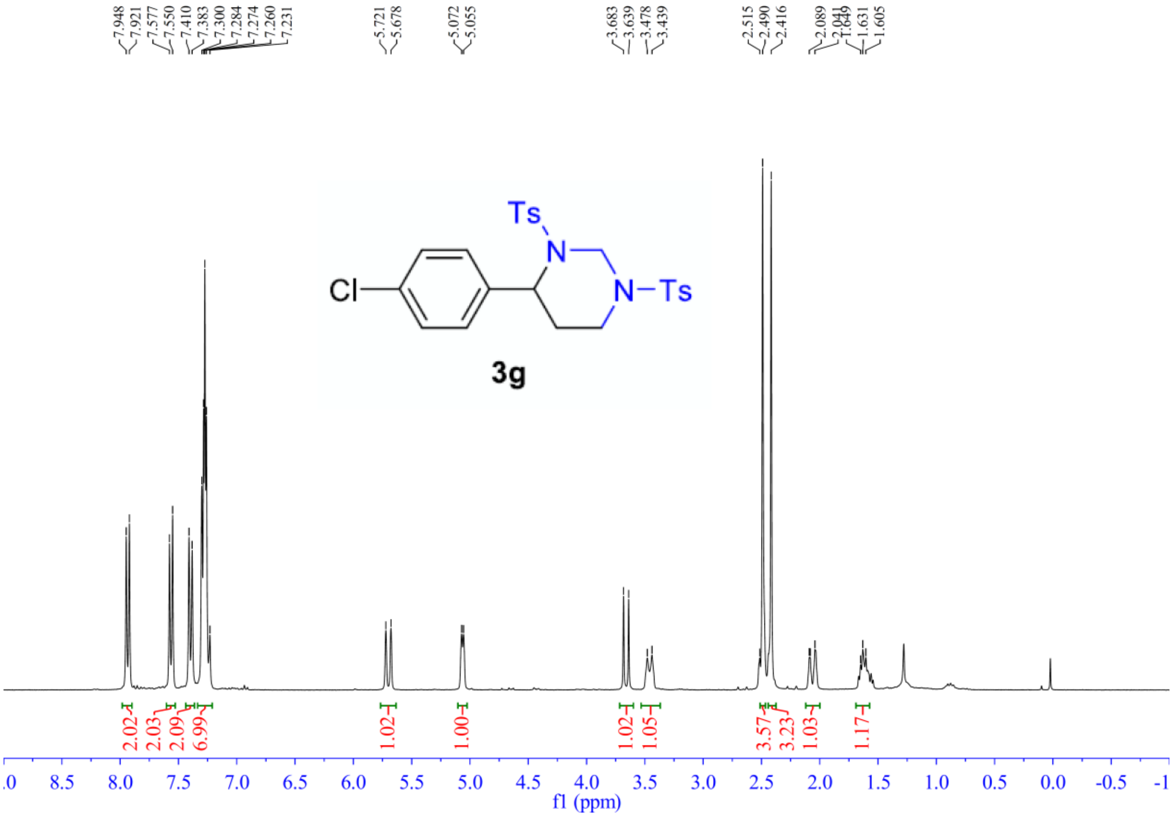

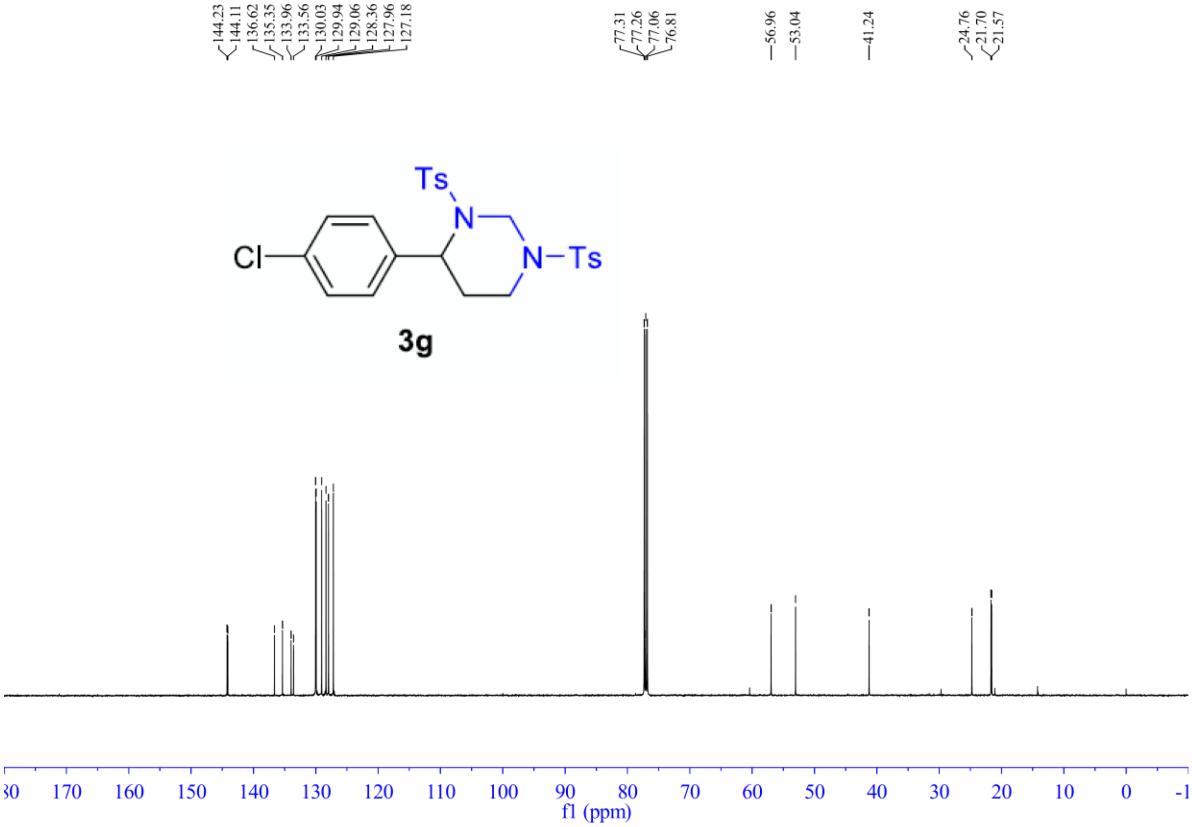

4-(2-chlorophenyl)-1, 3-ditosylhexahydropyrimidine (3h):

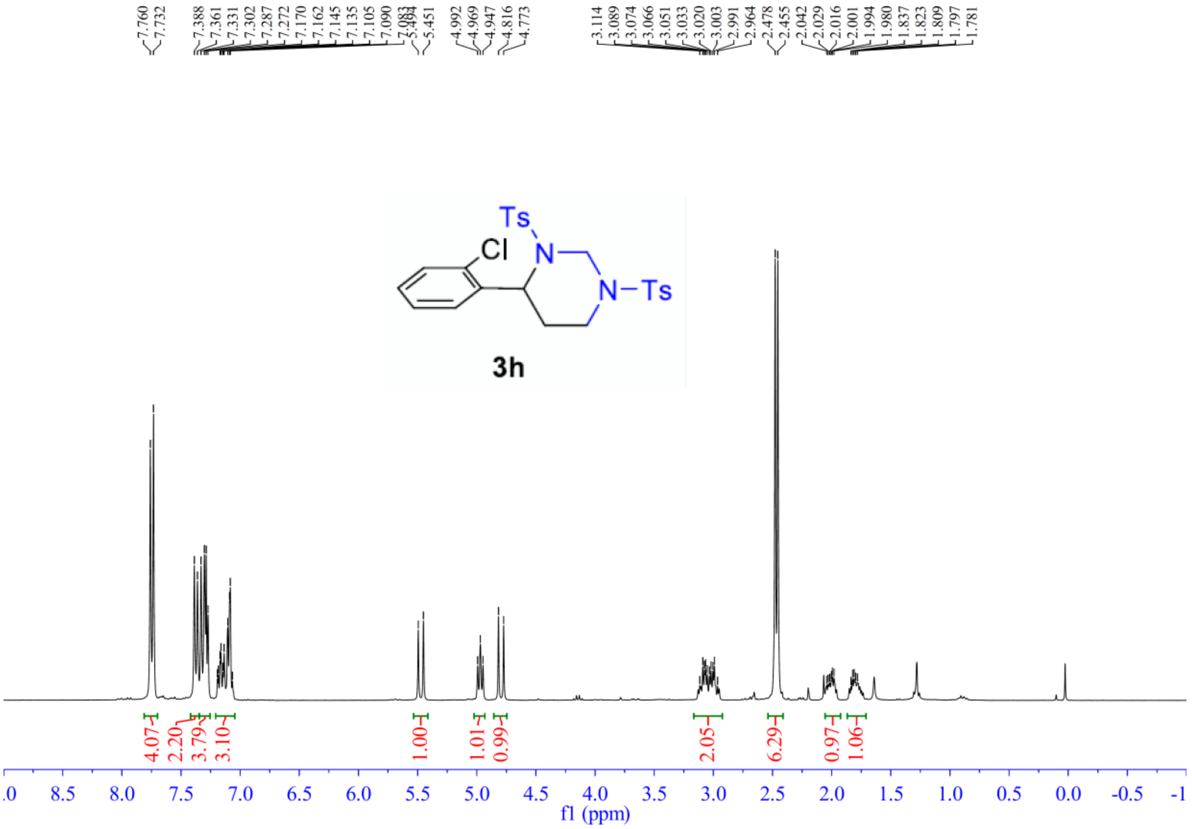

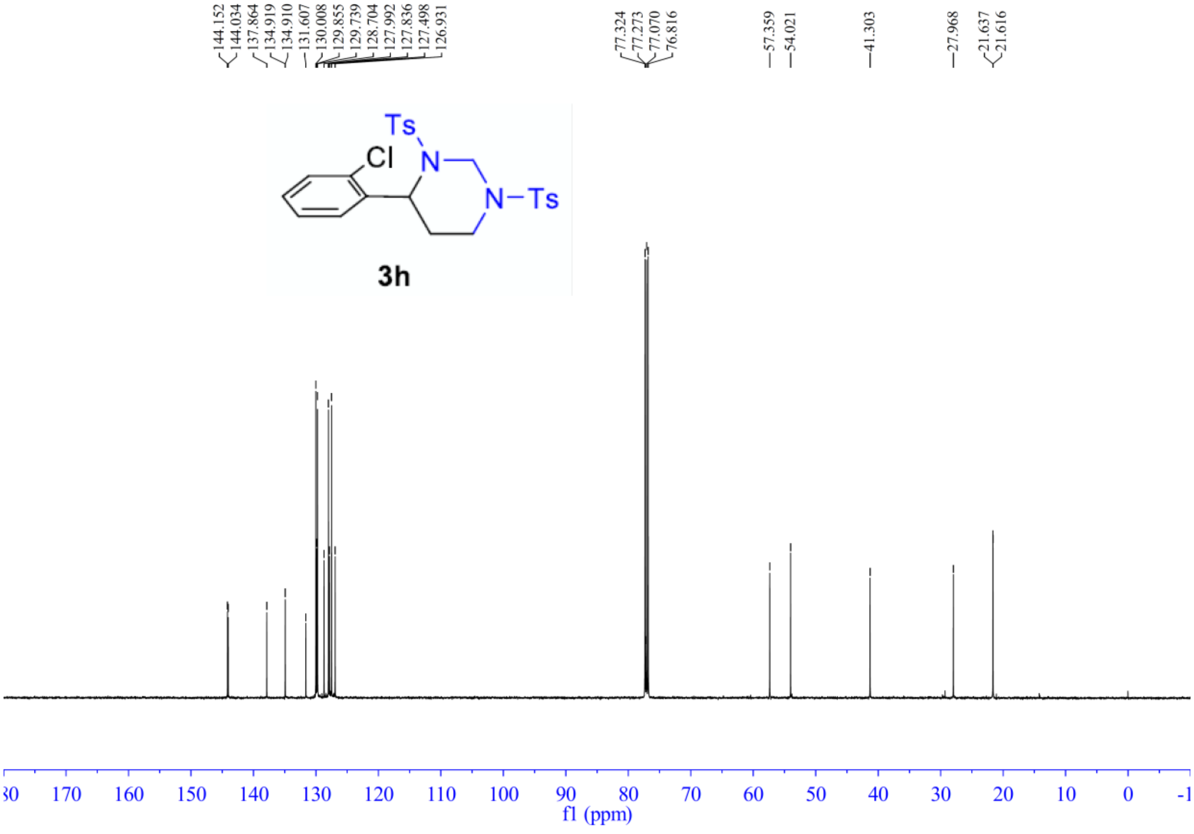

4-(4-bromophenyl)-1, 3-ditosylhexahydropyrimidine (3i):

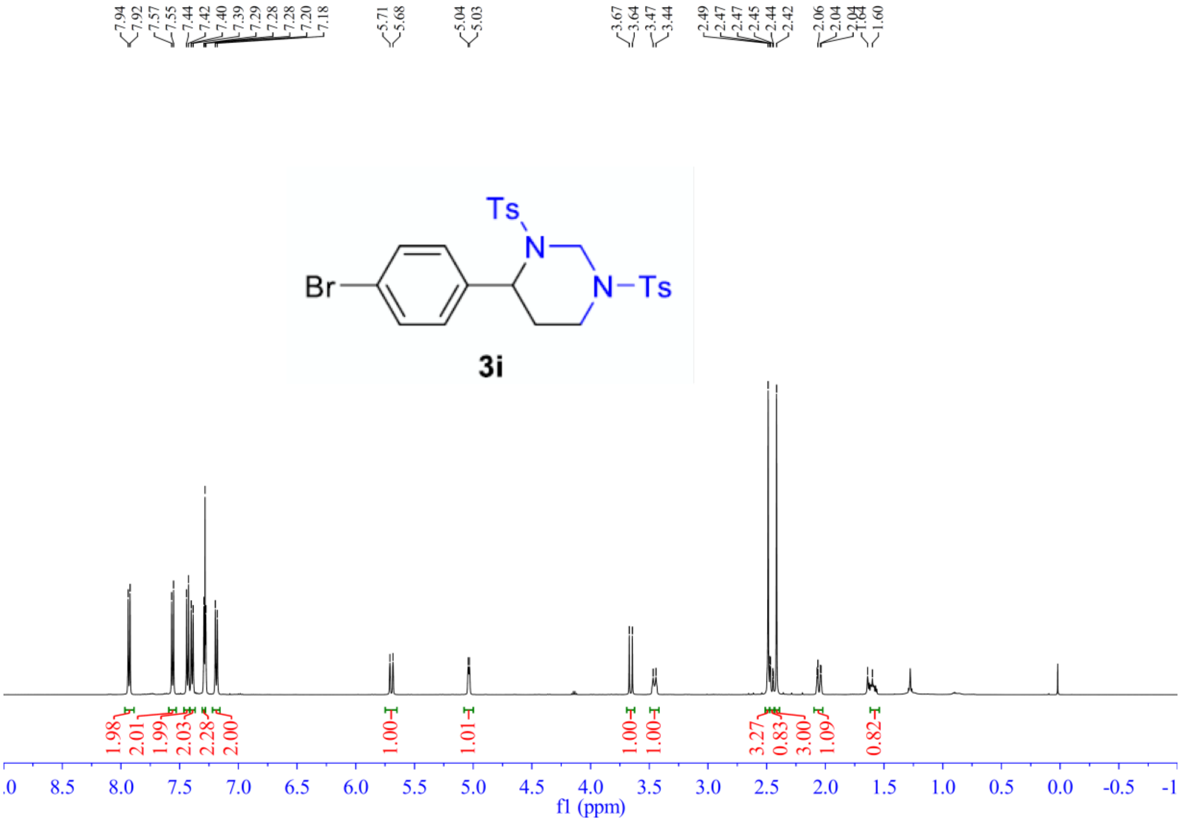

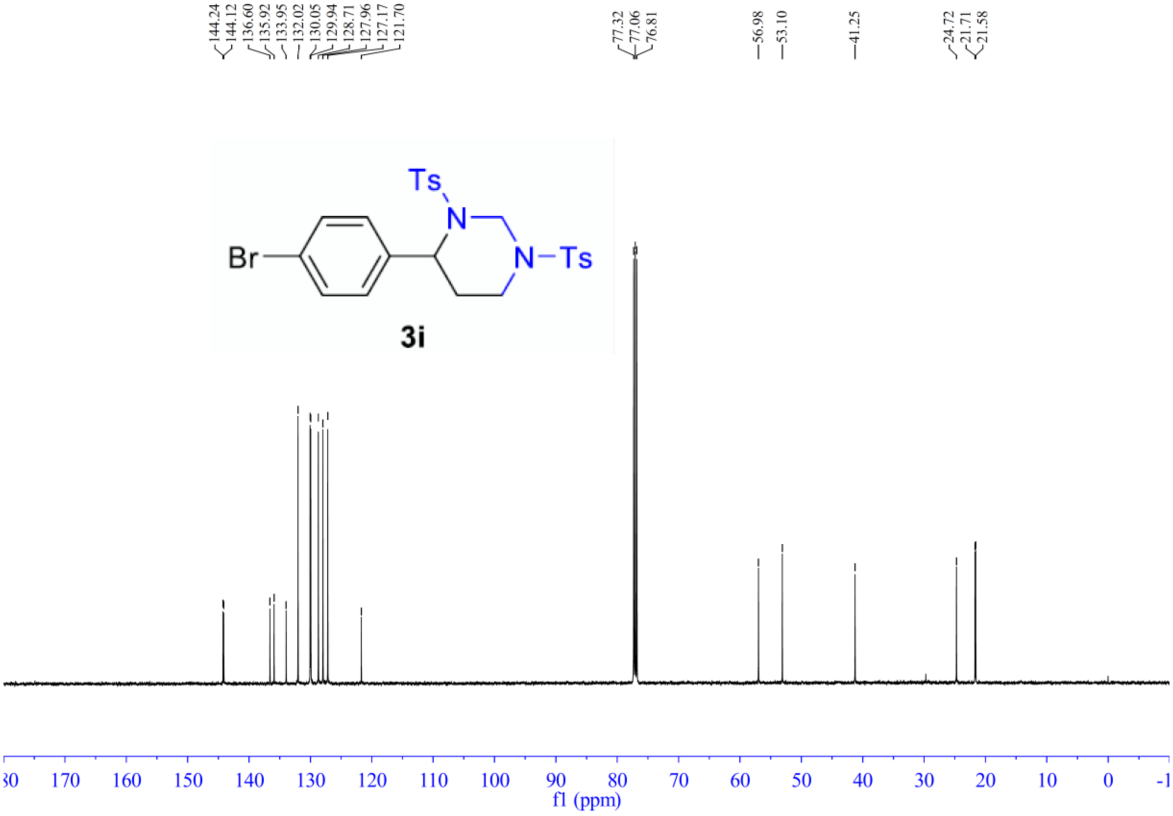

4-(2-bromophenyl)-1,3-ditosylhexahydropyrimidine (3j):

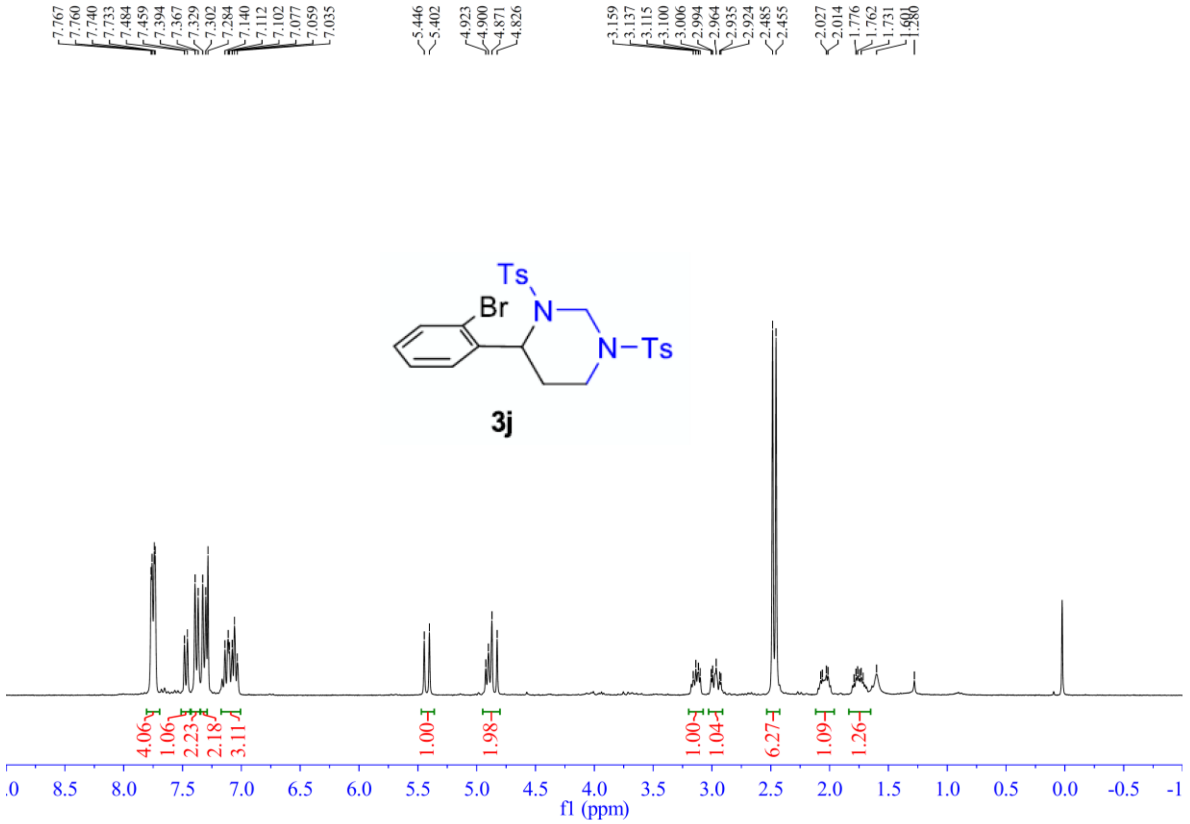

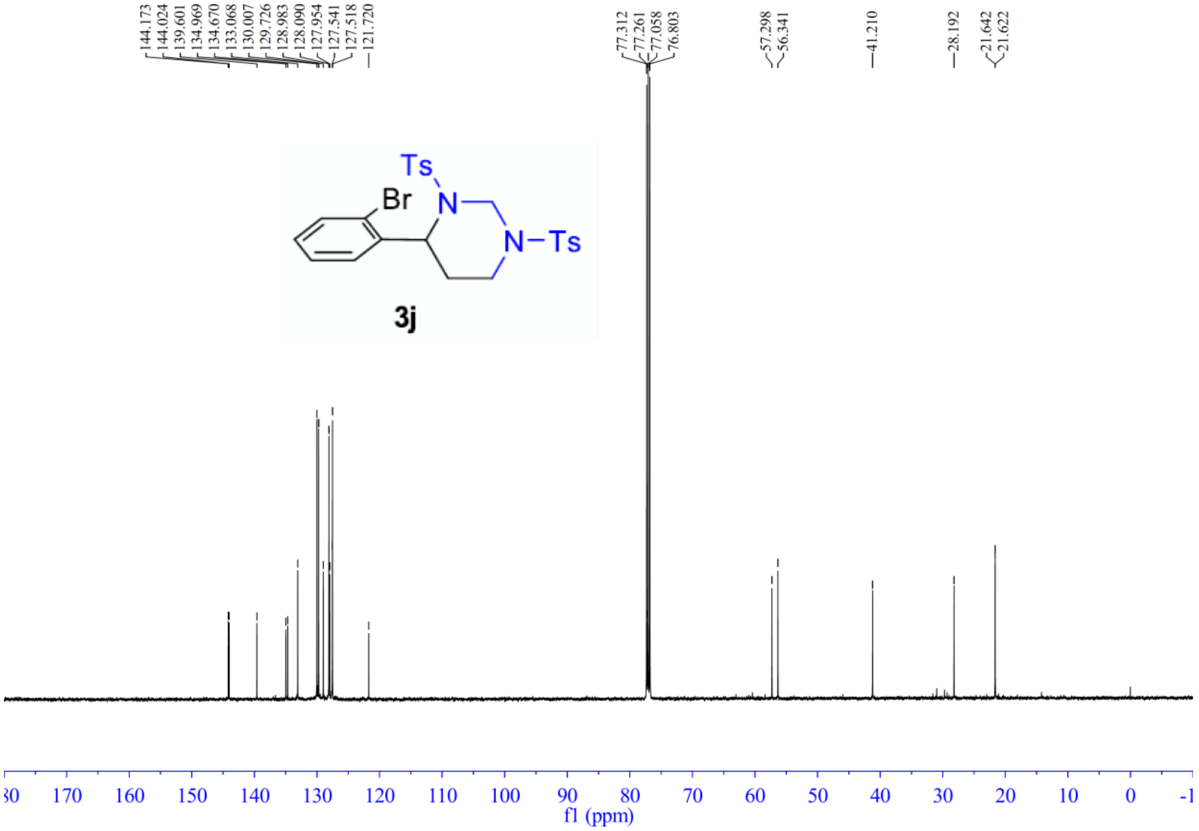

4-(naphthalen-2-yl)-1, 3-ditosylhexahydropyrimidine (3k):

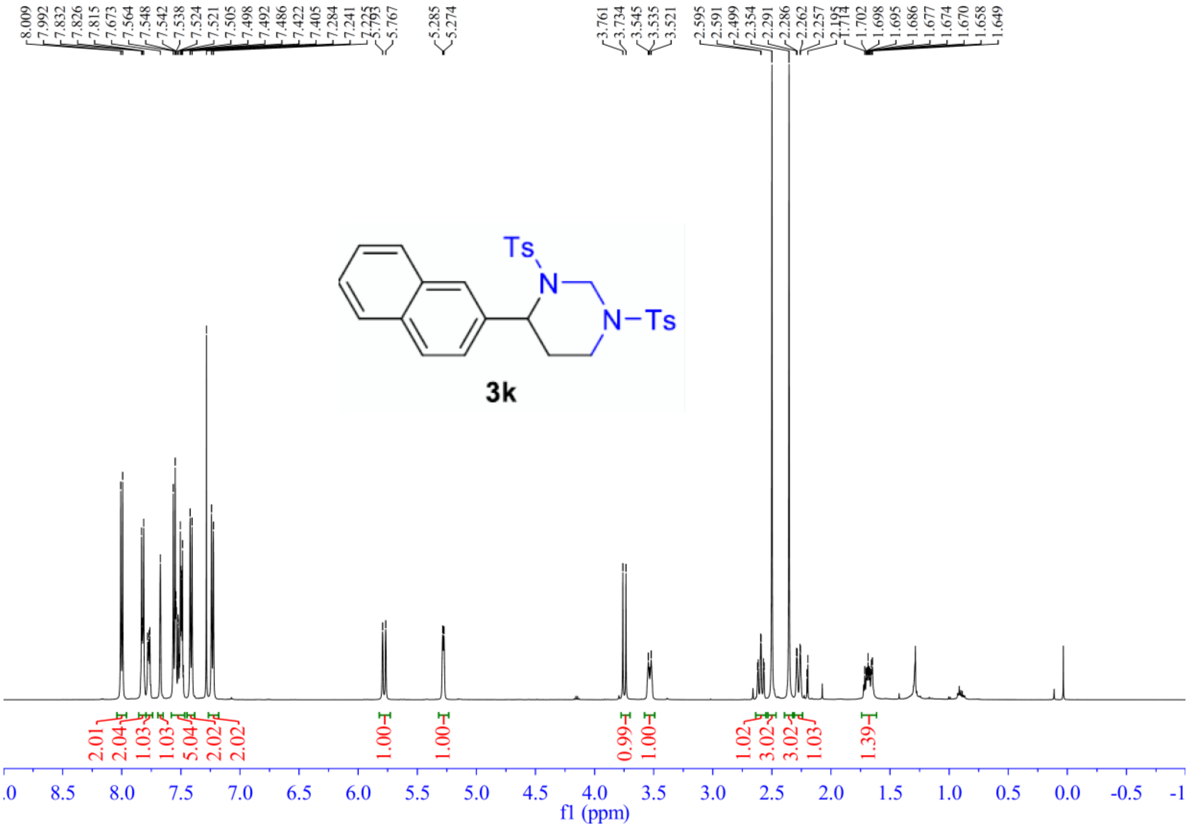

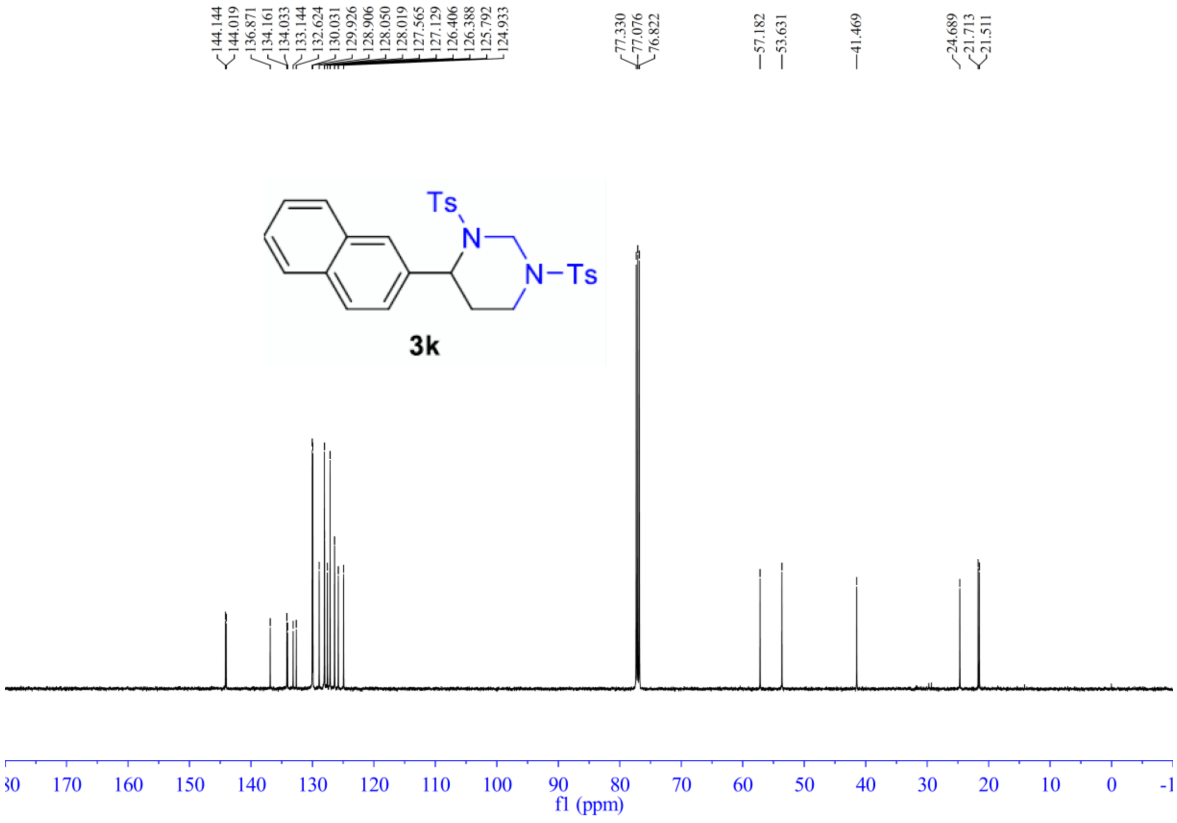

1, 3-ditosyl-1, 2, 3, 4, 4a, 5, 6, 8a-octahydroquinazoline (3l):

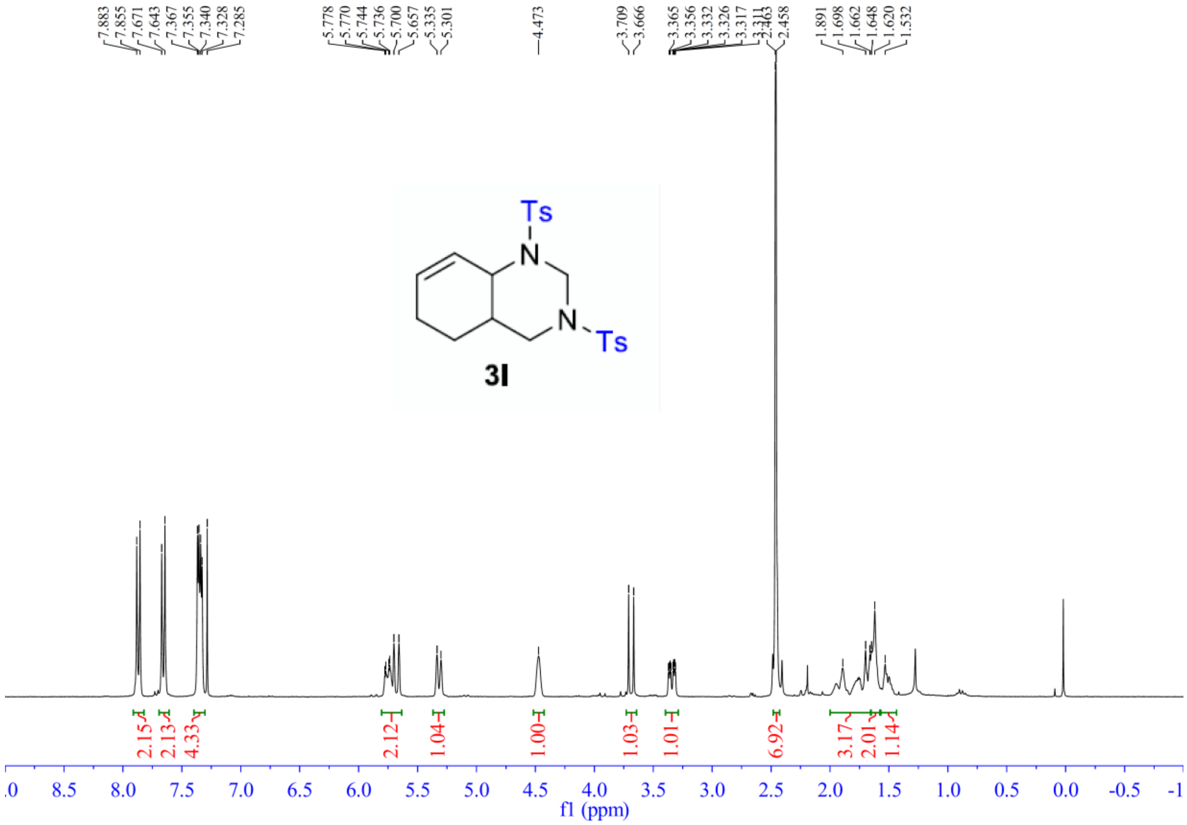

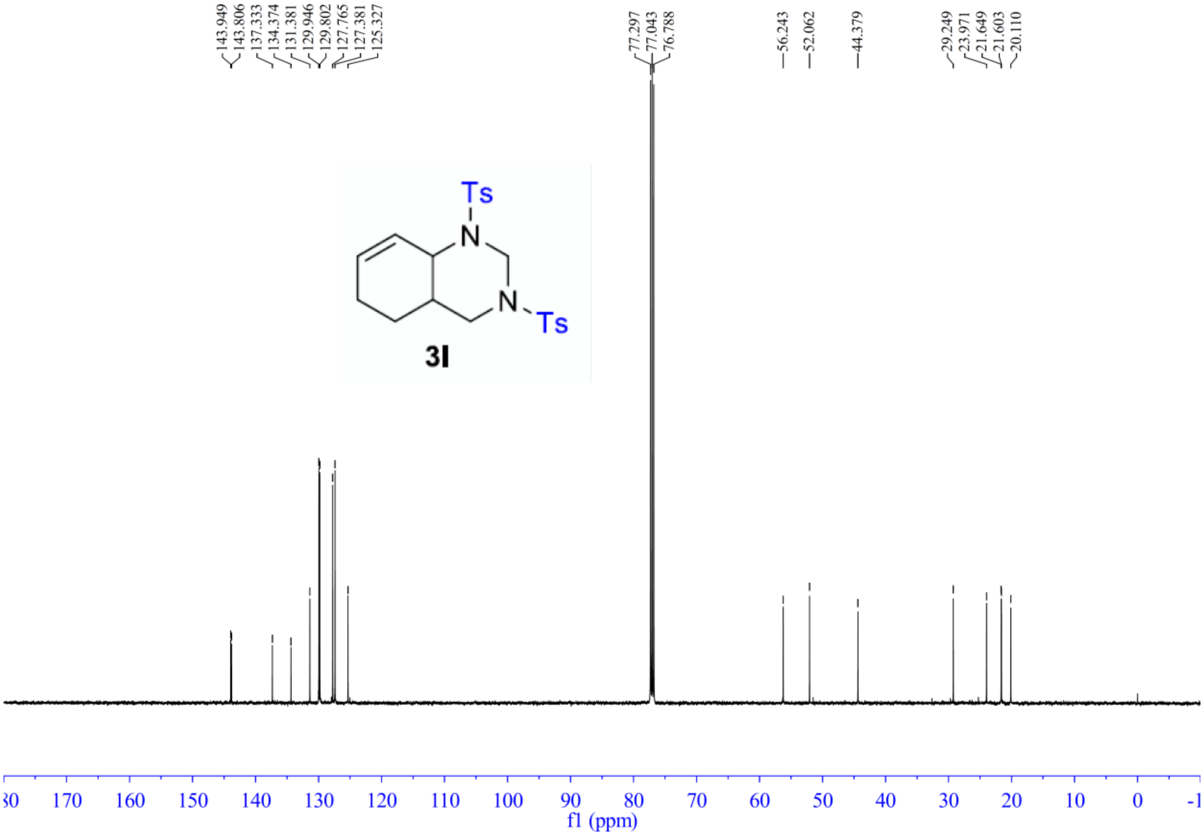

1, 3-ditosyl-2, 3, 4, 4a, 5, 9b-hexahydro-1H- indeno[1, 2-d]pyrimidine (3m):

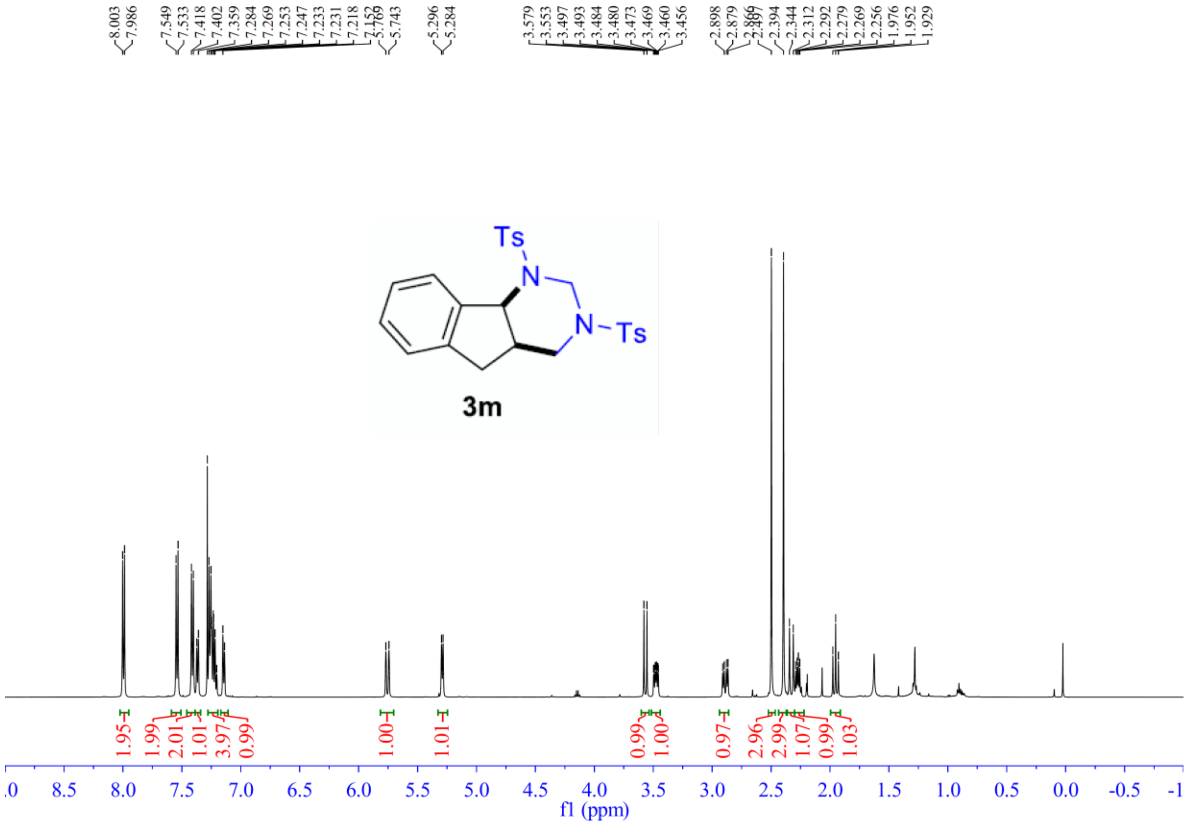

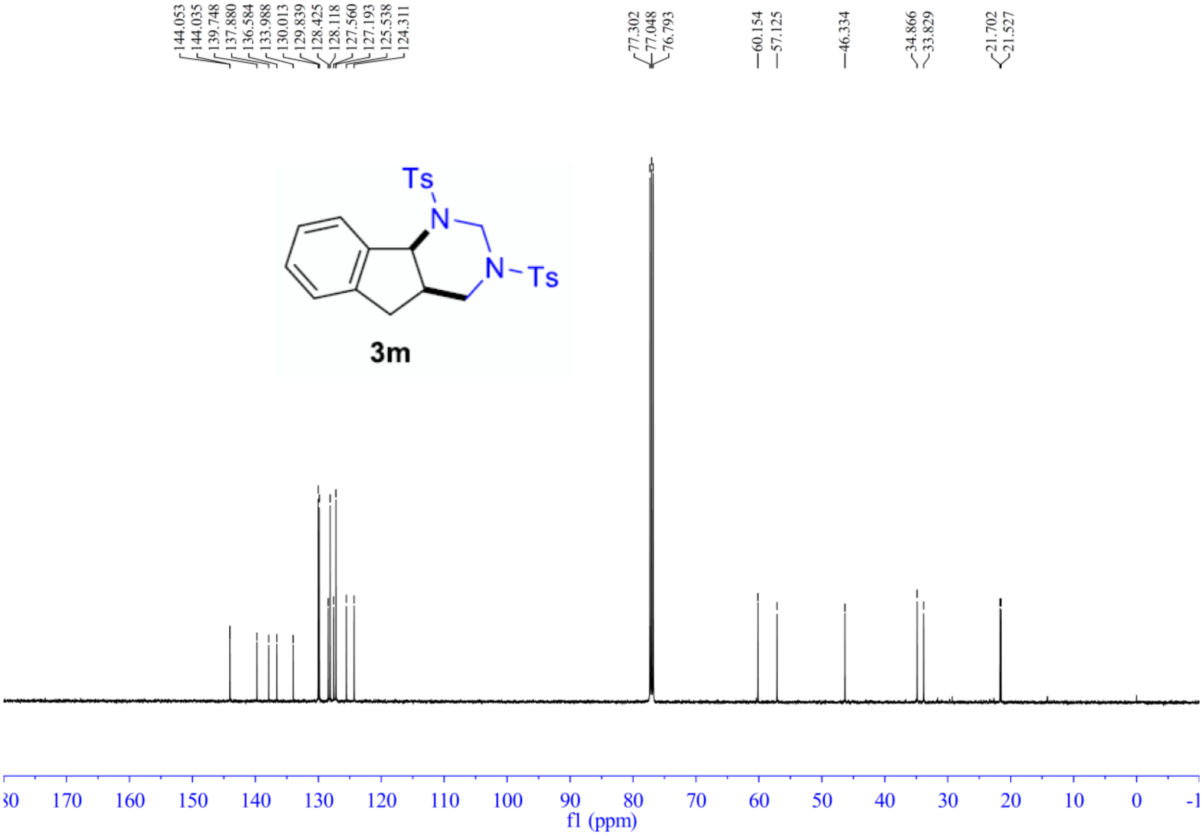

5-methyl-4-phenyl-1, 3-ditosylhexahydropyrimidine (3n):

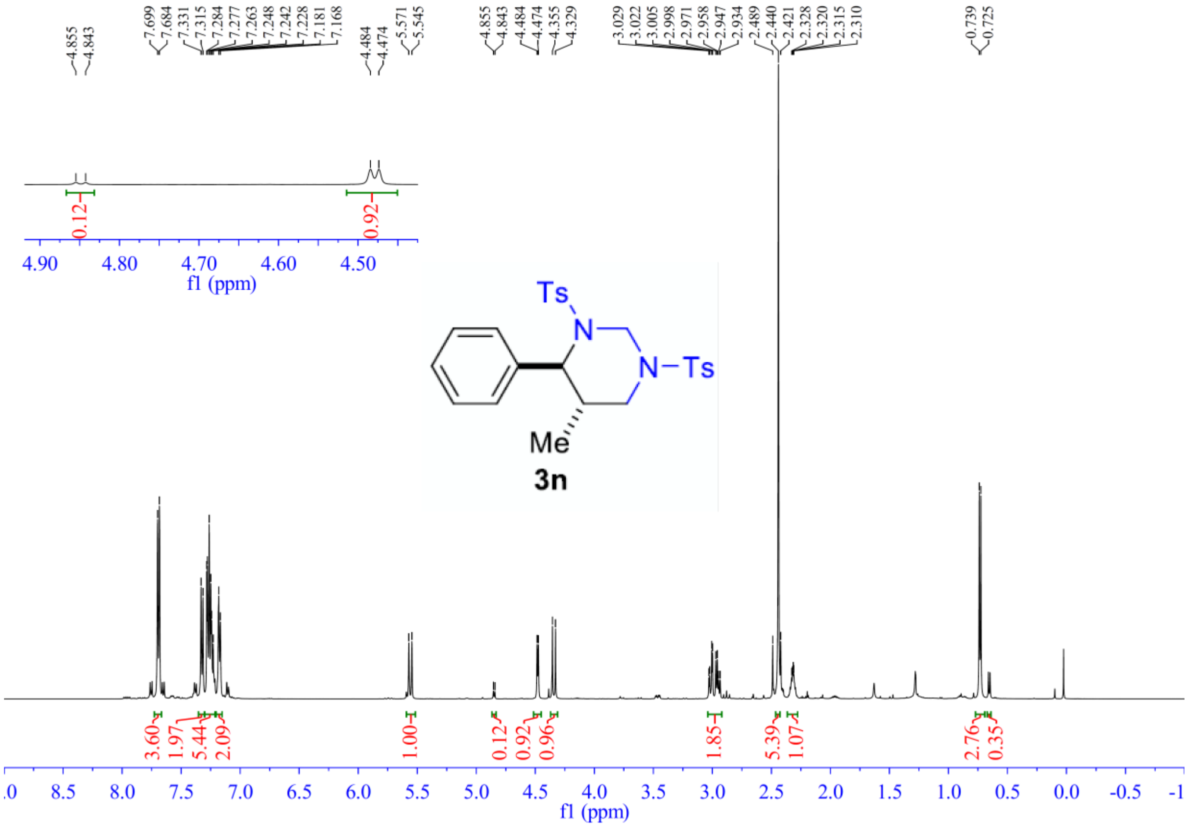

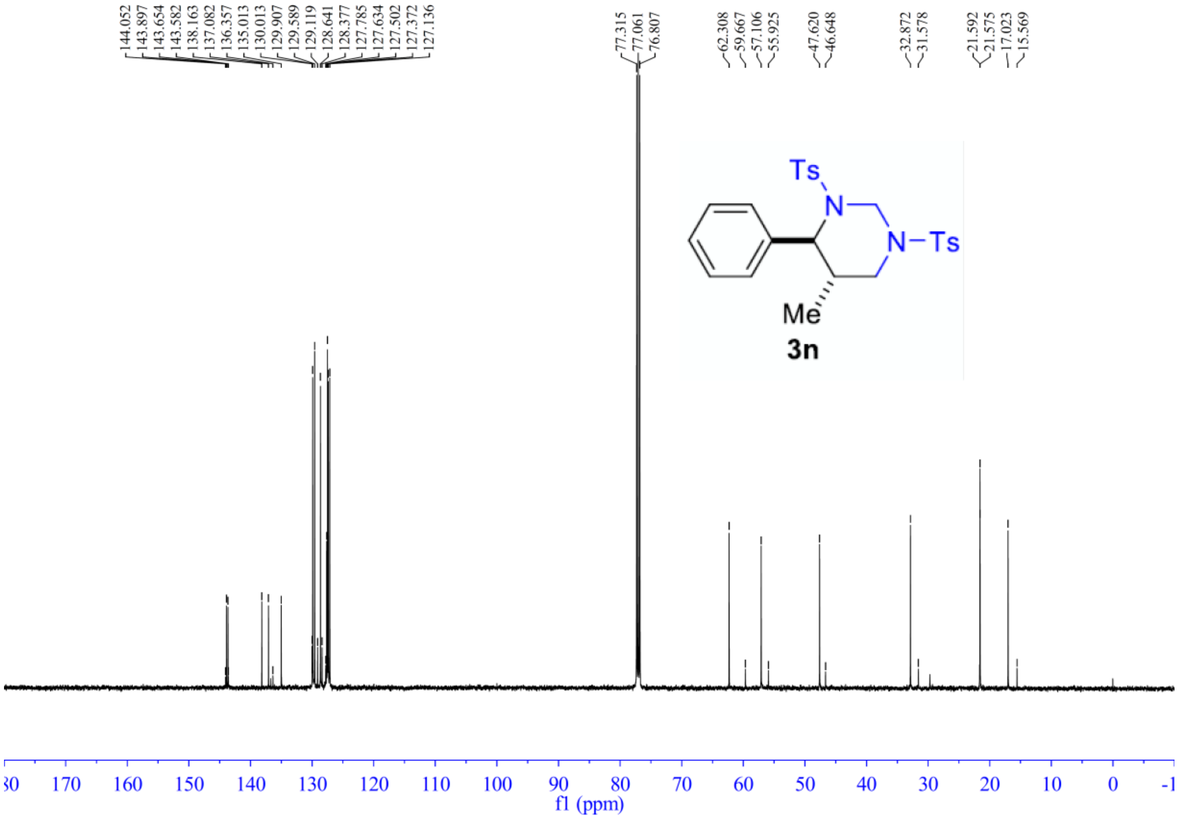

4, 5-diphenyl-1, 3-ditosylhexahydropyrimidine (3o):

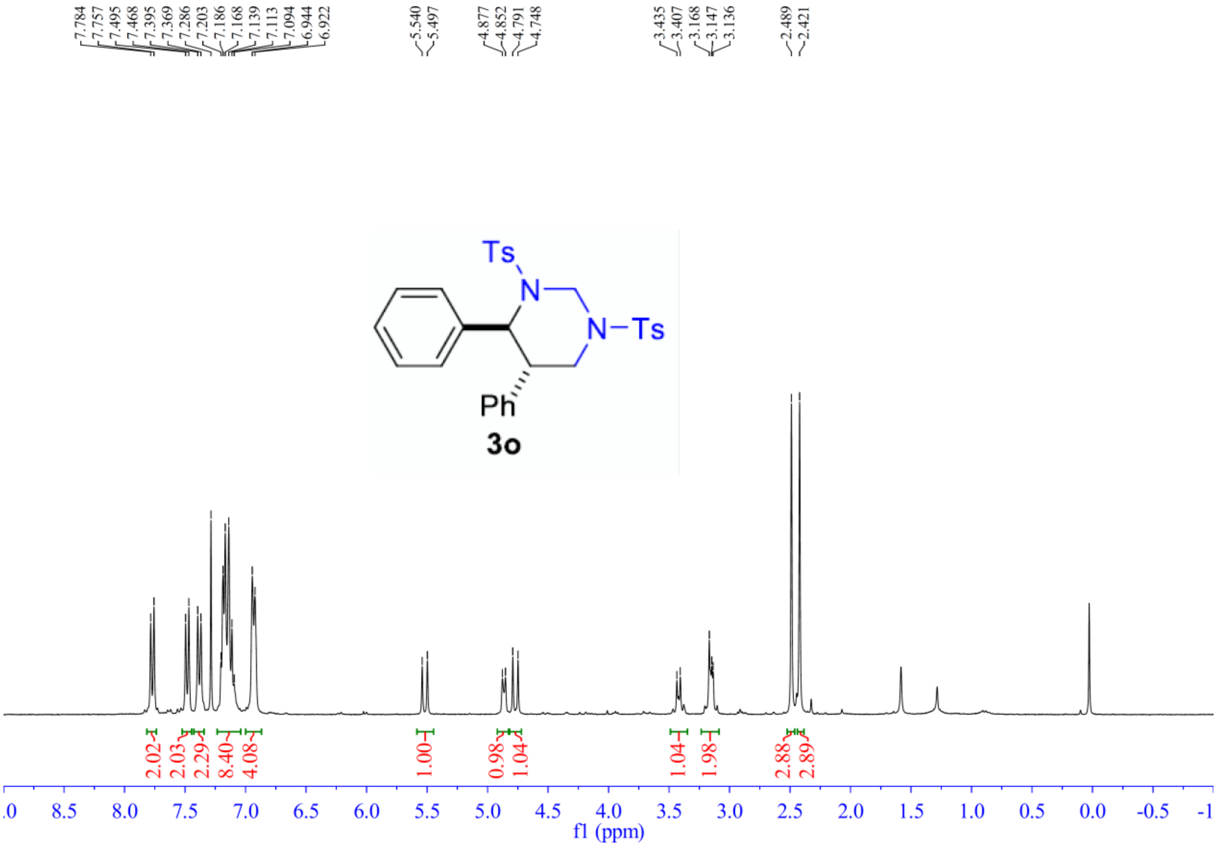

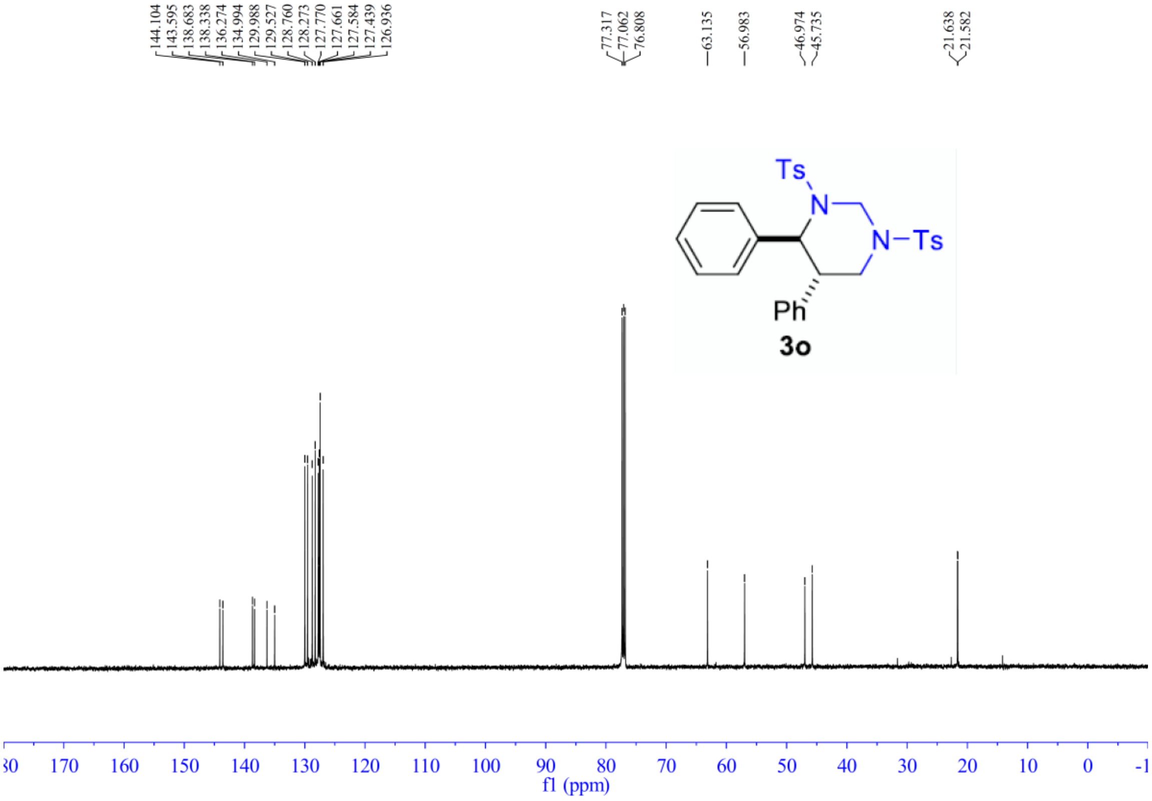

1, 3-bis(naphthalen-2-ylsulfonyl)-4-phenylhexahydropyrimidine (3p):

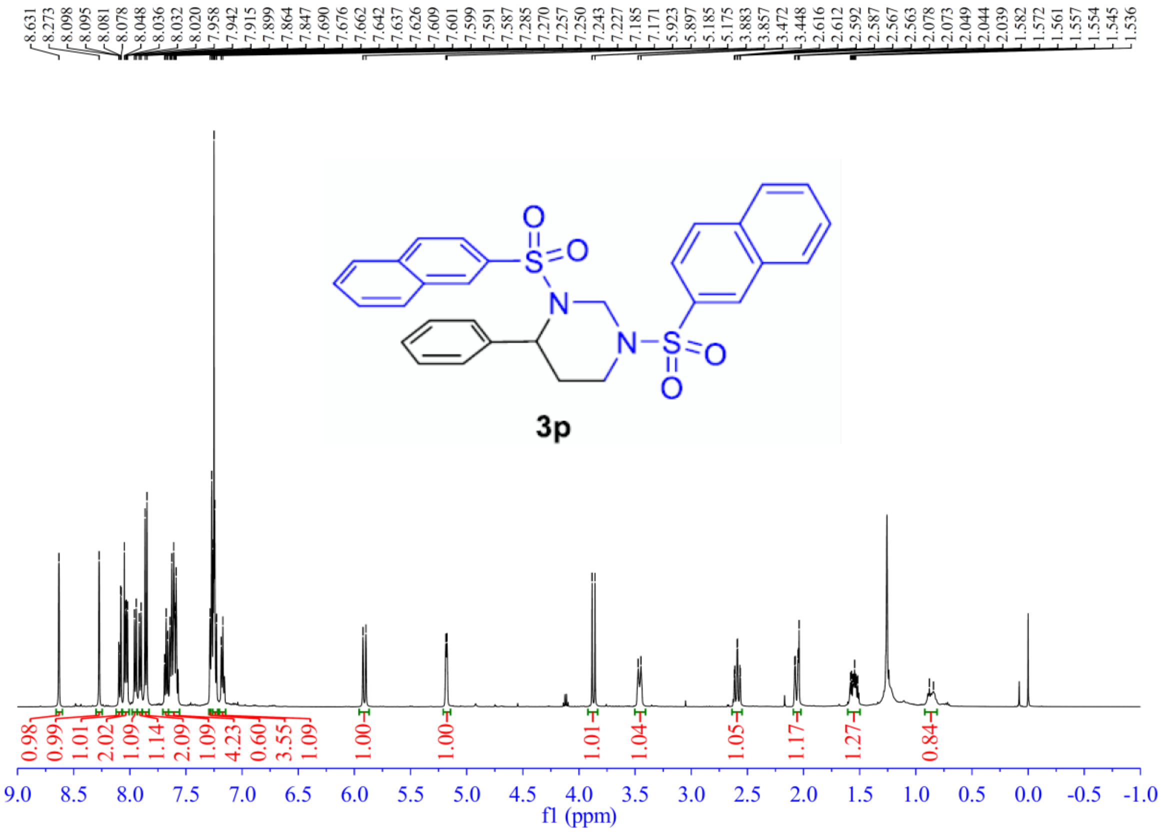

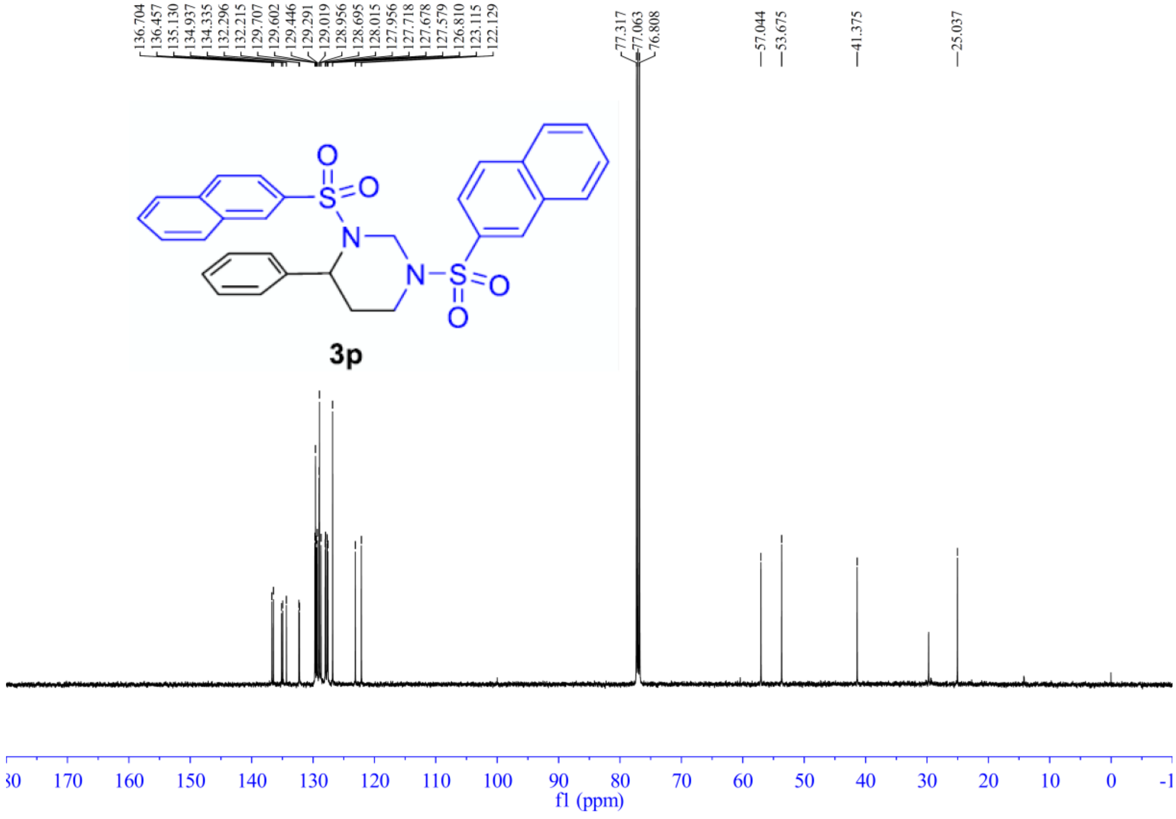

1, 3-bis(mesitylsulfonyl)-4-phenylhexahydropyrimidine (3q):

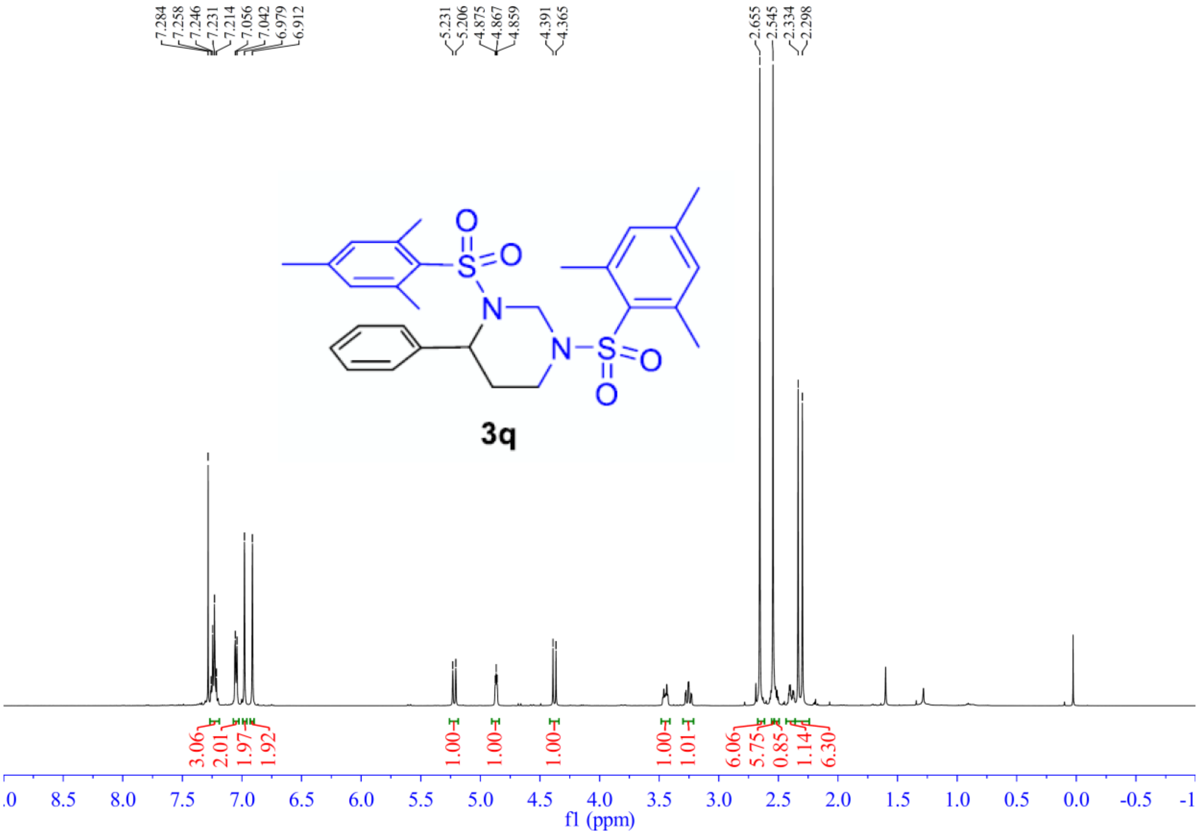

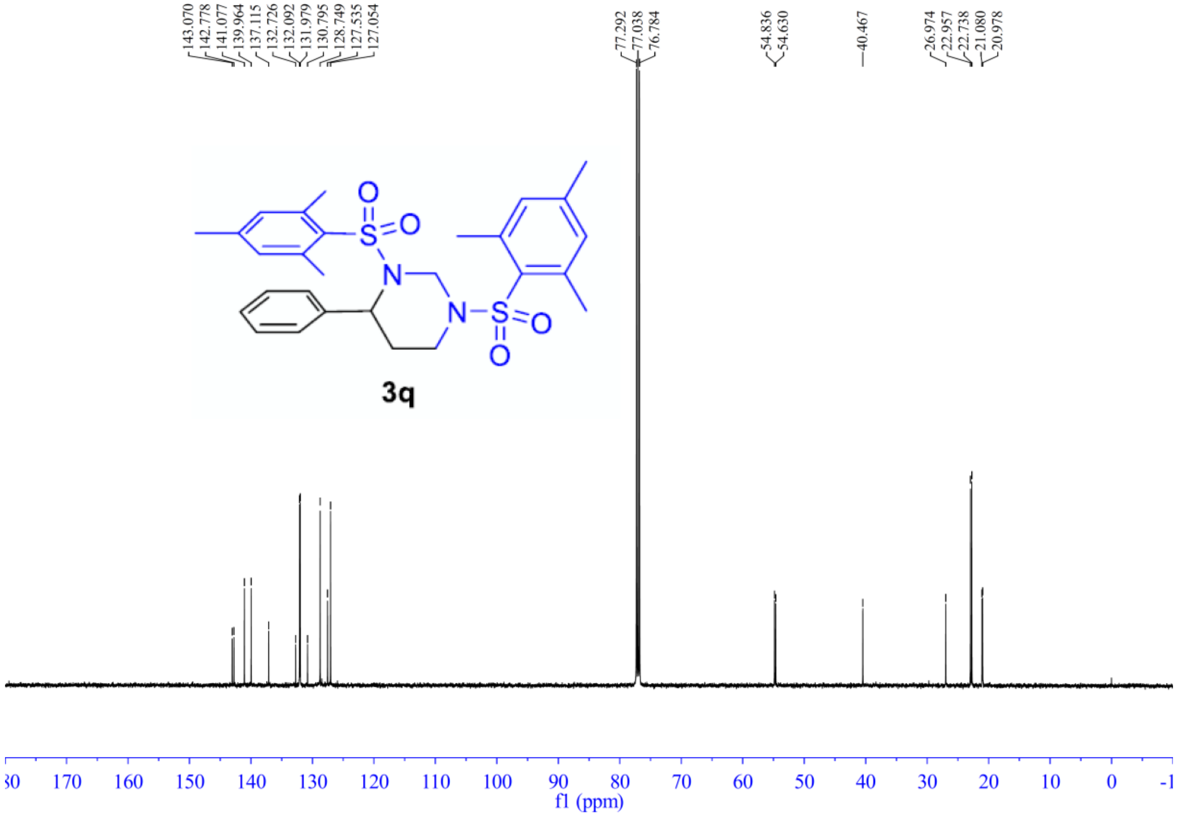

5-benzylidene-1, 3-ditosylhexahydropyrimidine (5a):

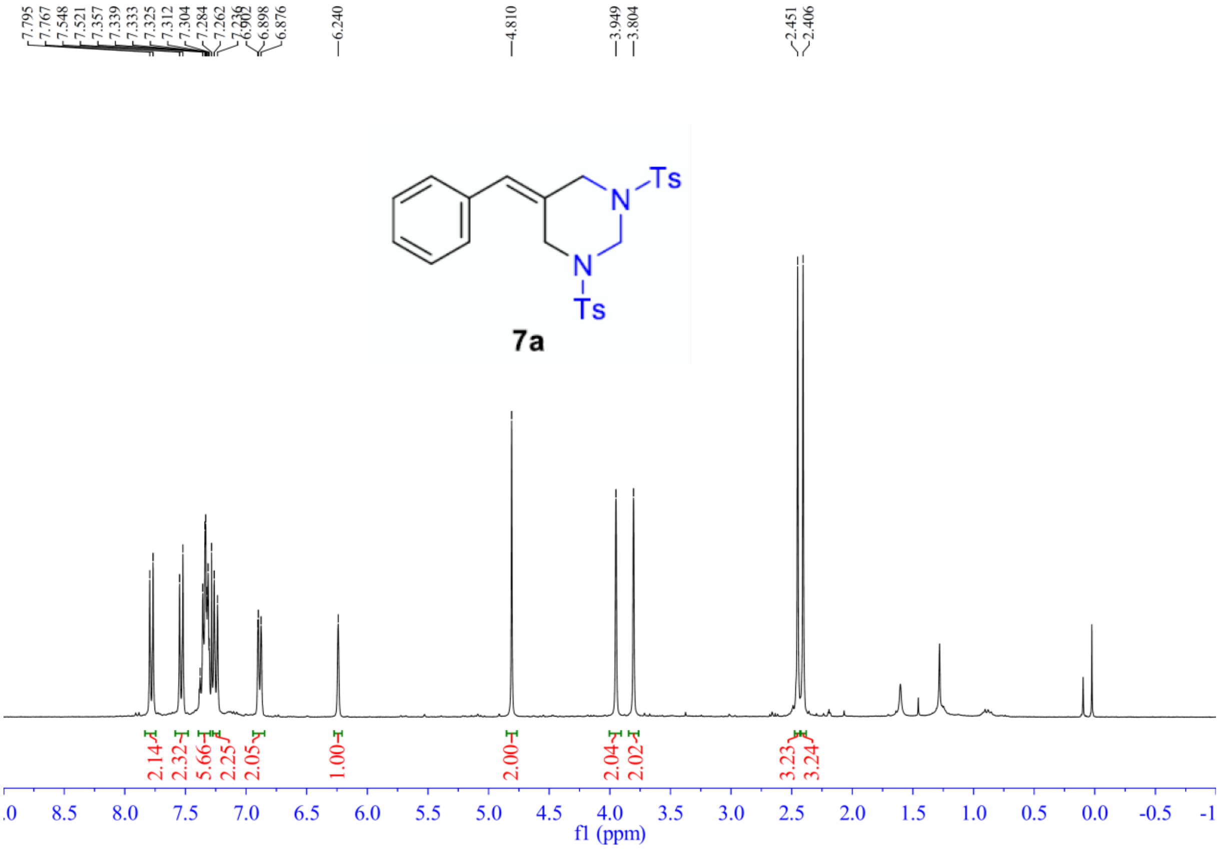

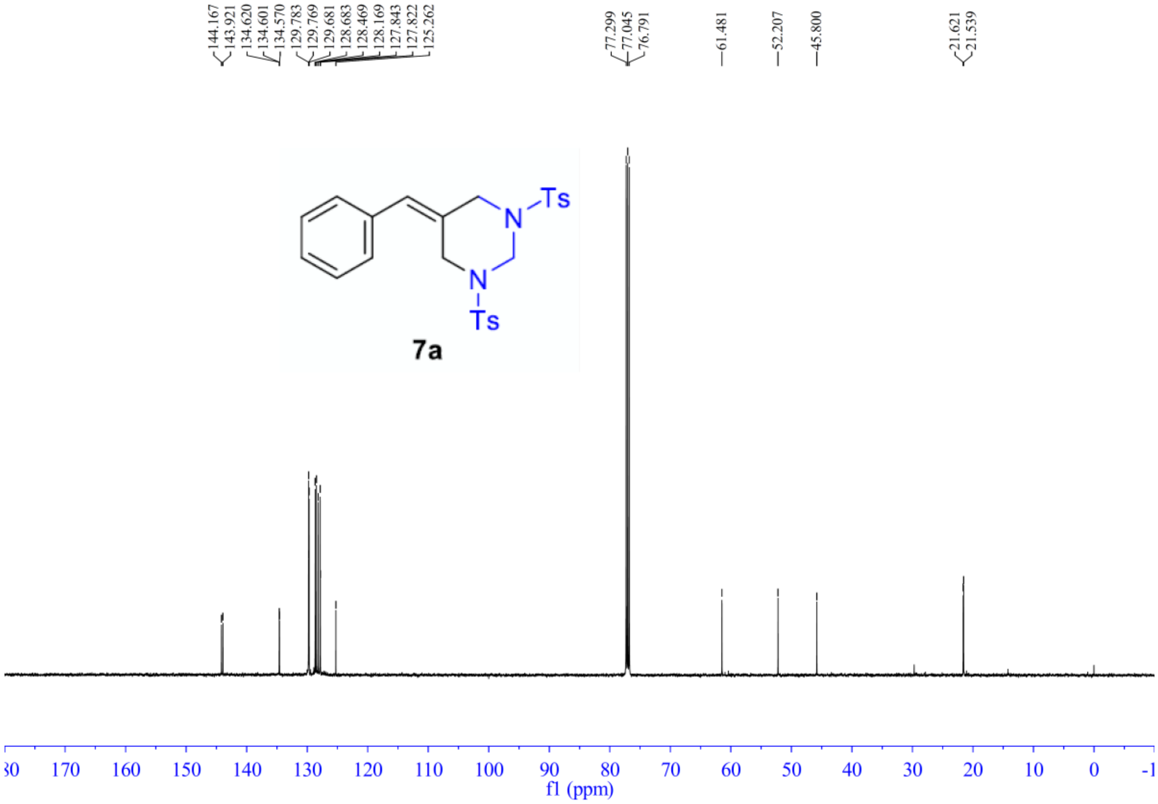

5-(4-methylbenzylidene)-1, 3-ditosylhexahydropyrimidine (5b):

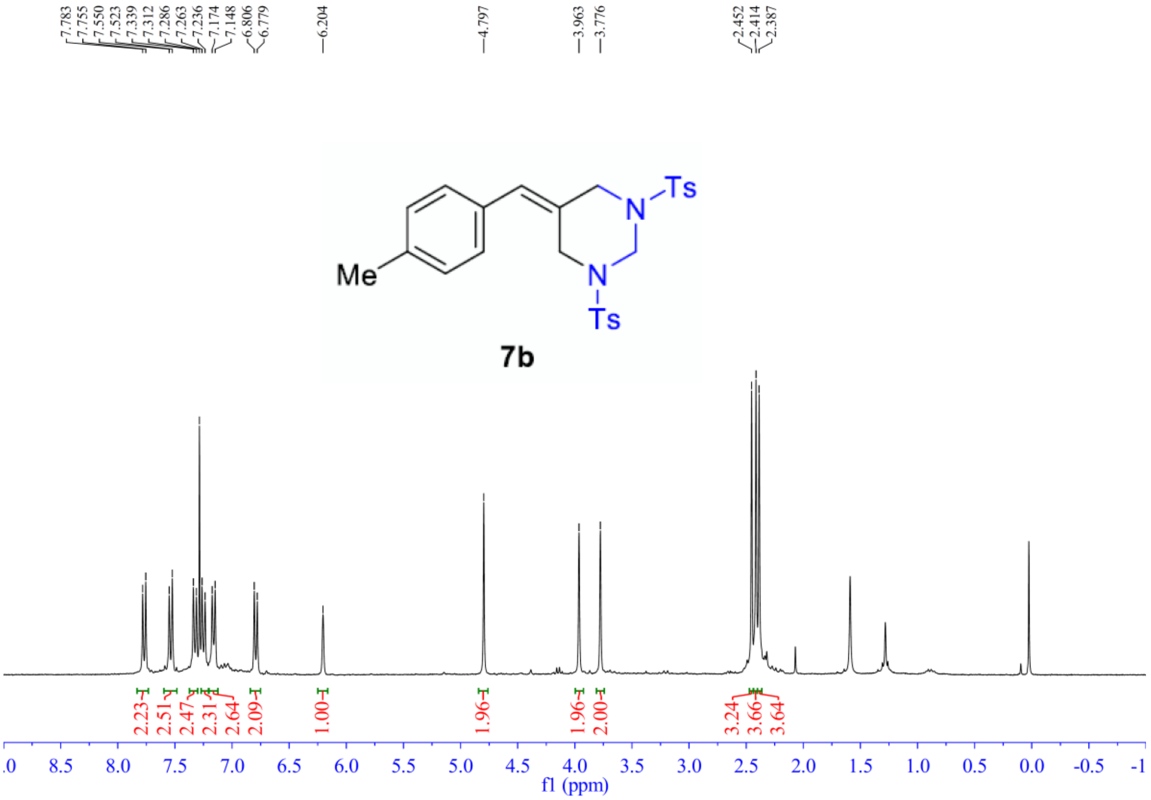

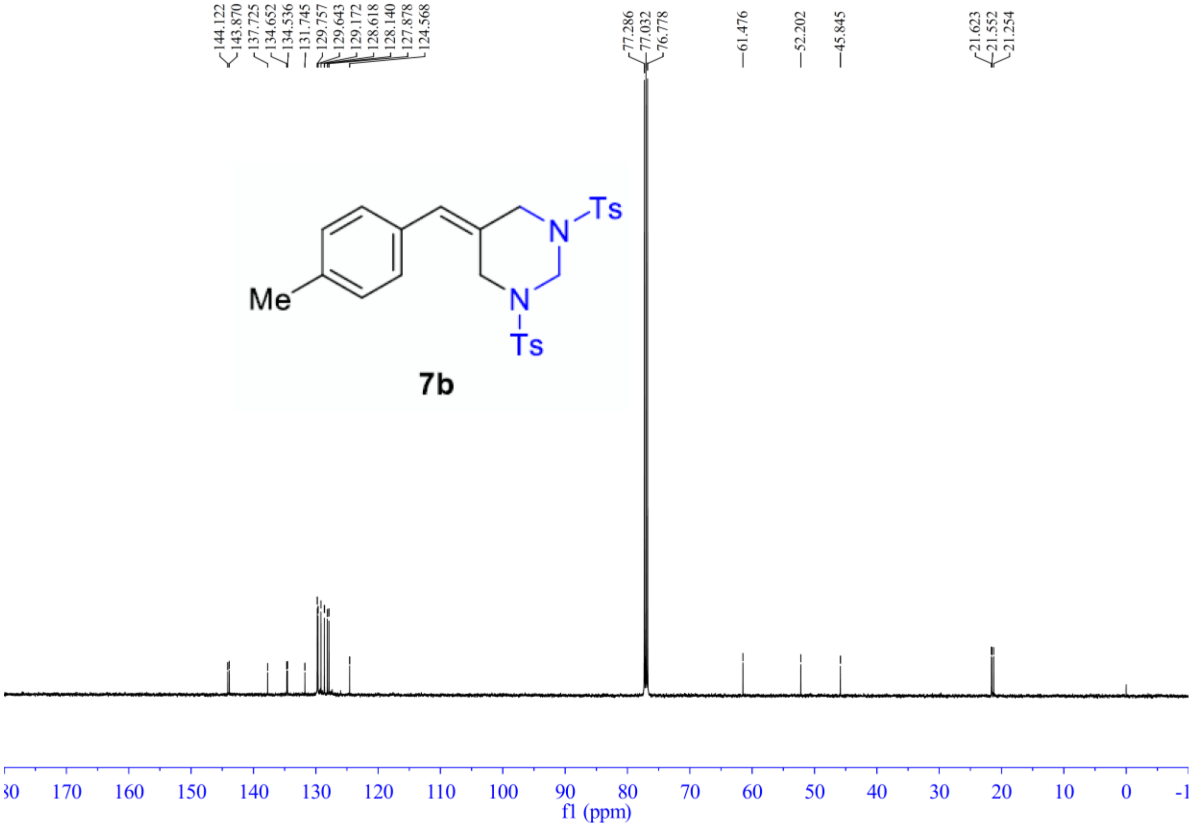

5-(3-methylbenzylidene)-1, 3-ditosylhexahydropyrimidine (5c):

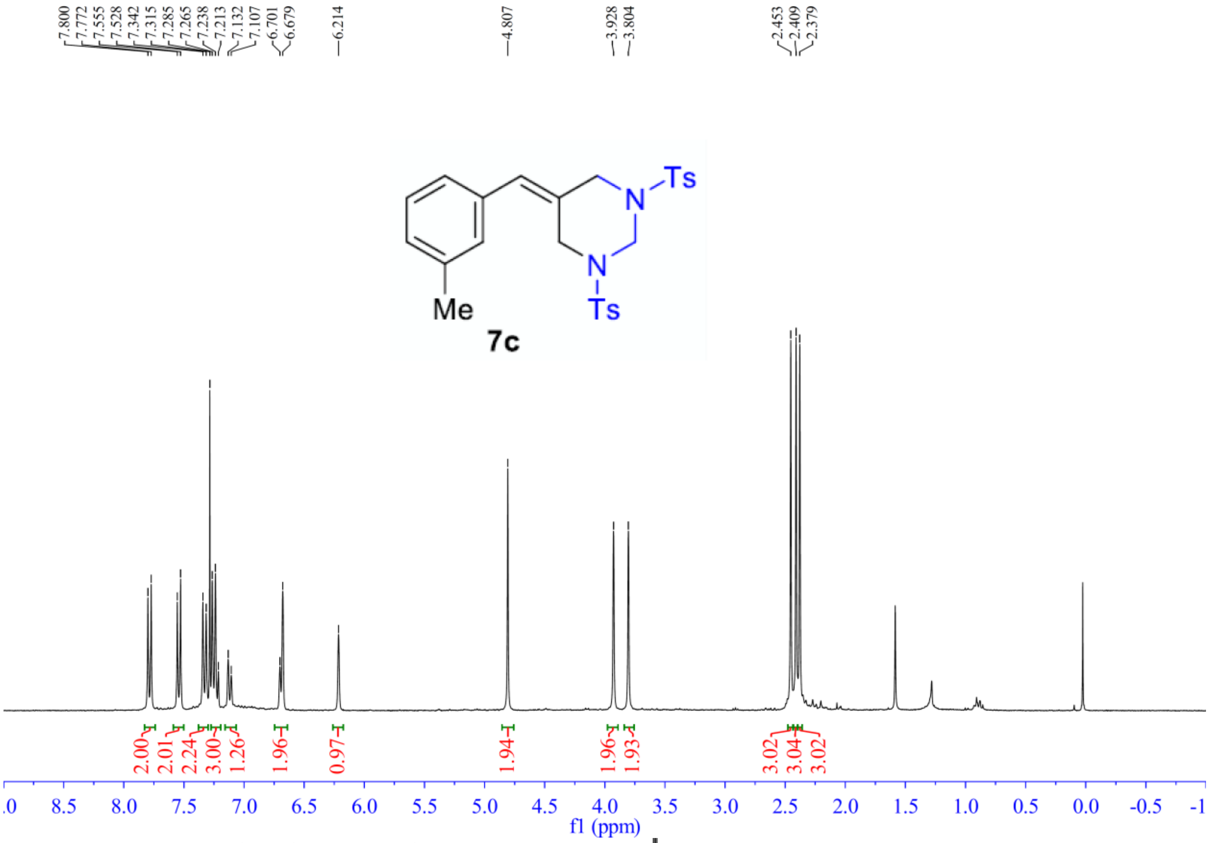

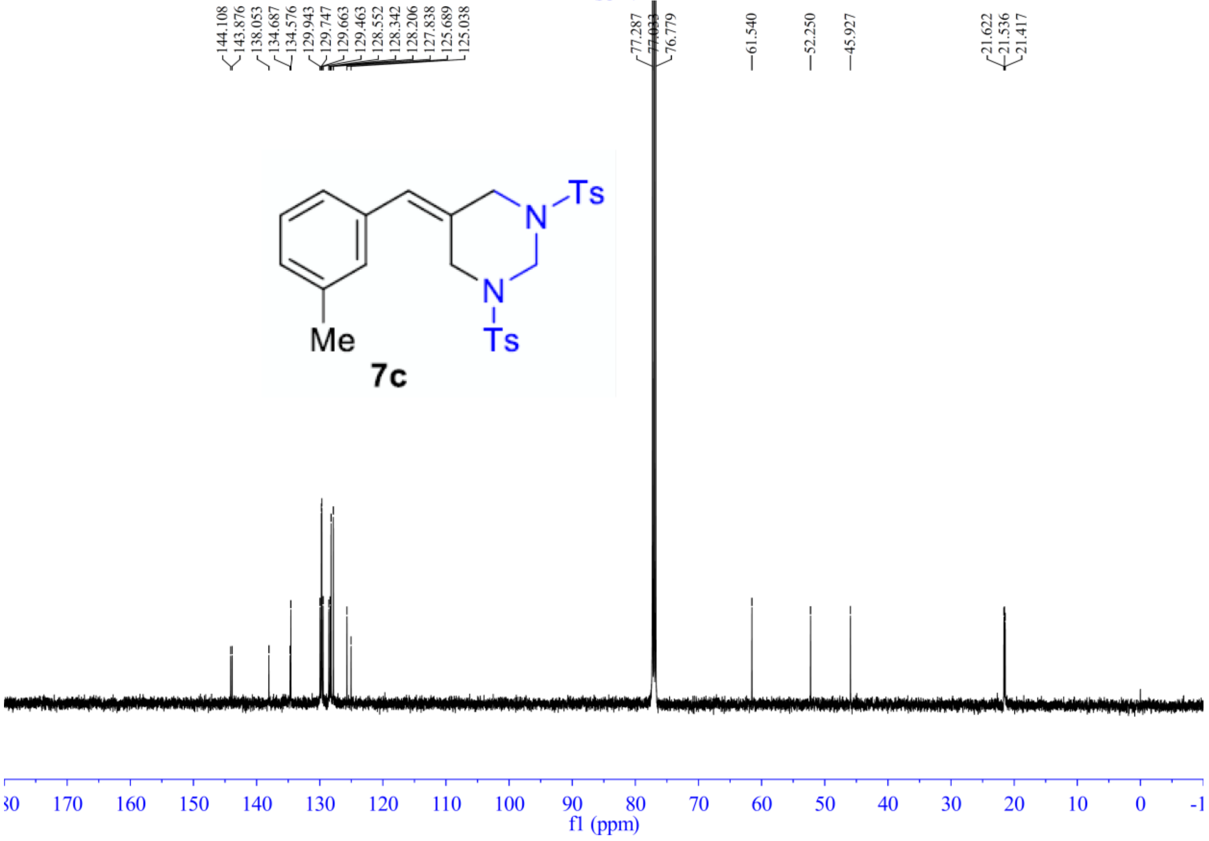

5-(2, 4-dimethylbenzylidene)-1, 3-ditosylhexahydropyrimidine (5d):

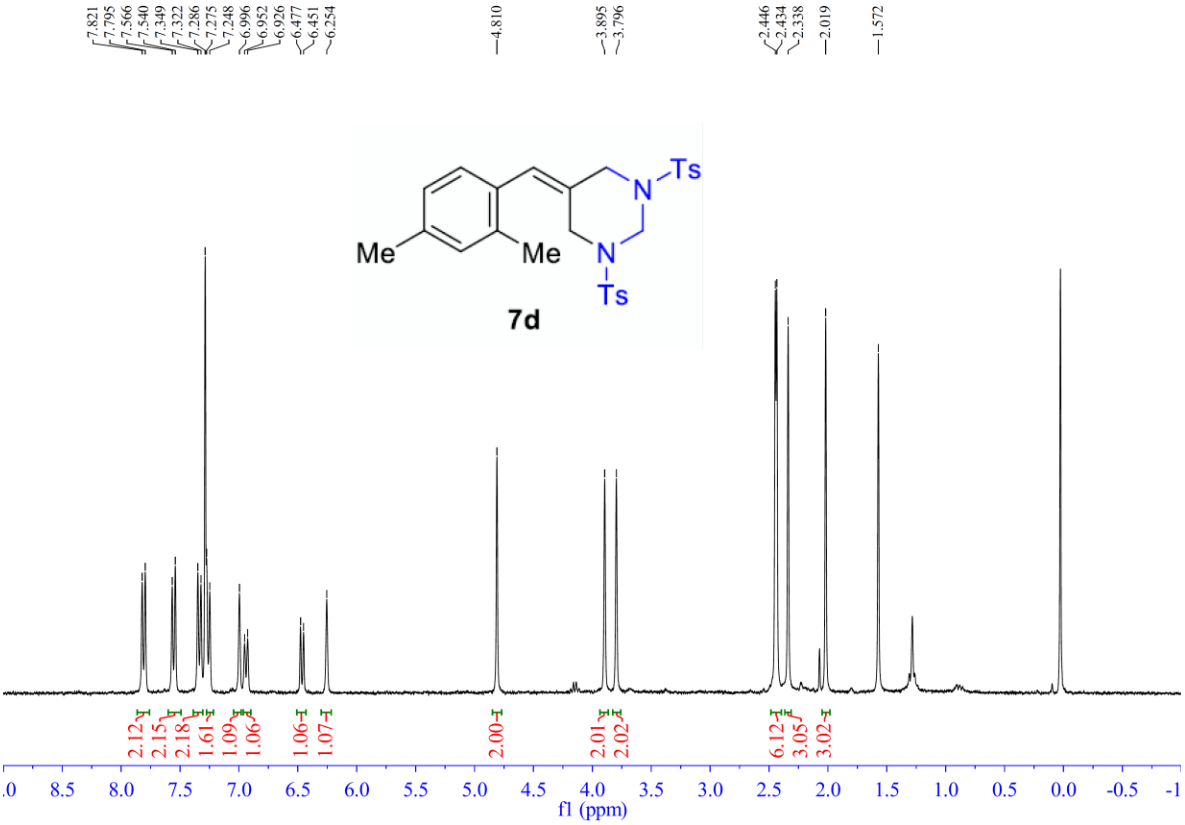

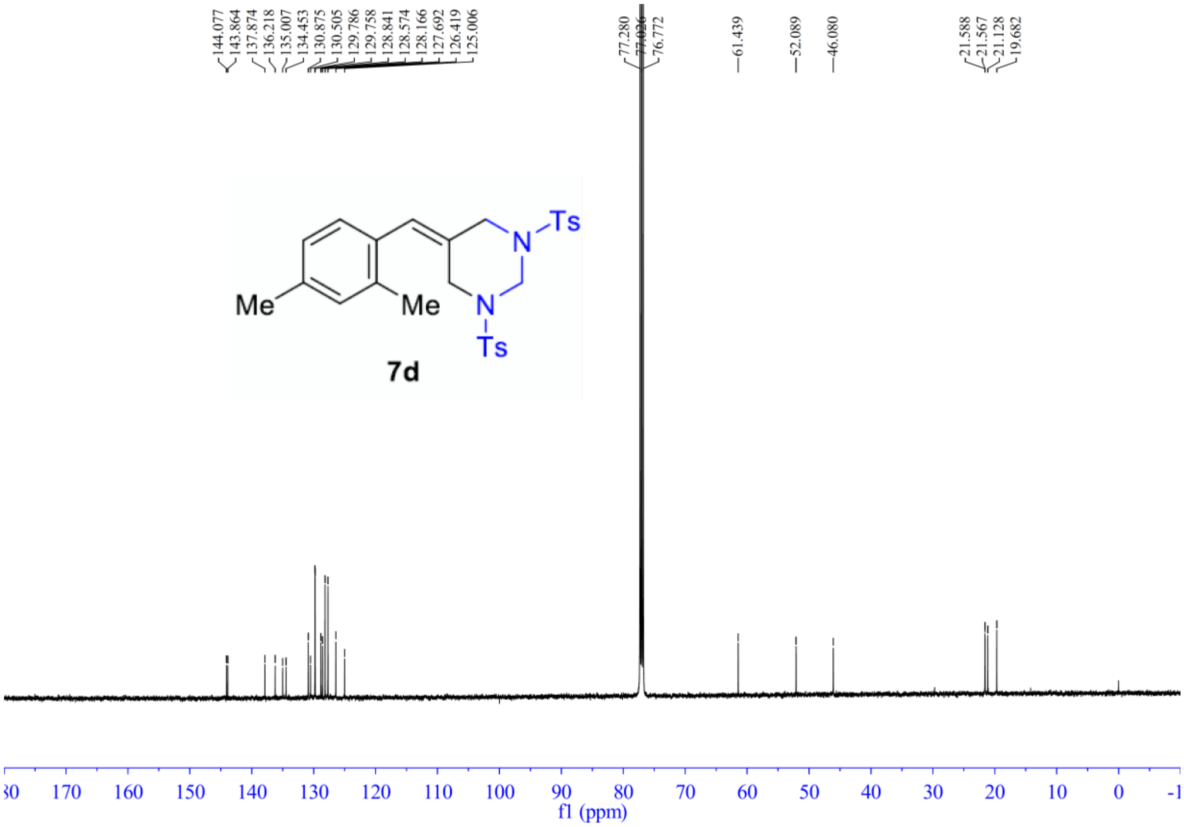

5-(4-chlorobenzylidene)-1, 3-ditosylhexahydropyrimidine (5e):

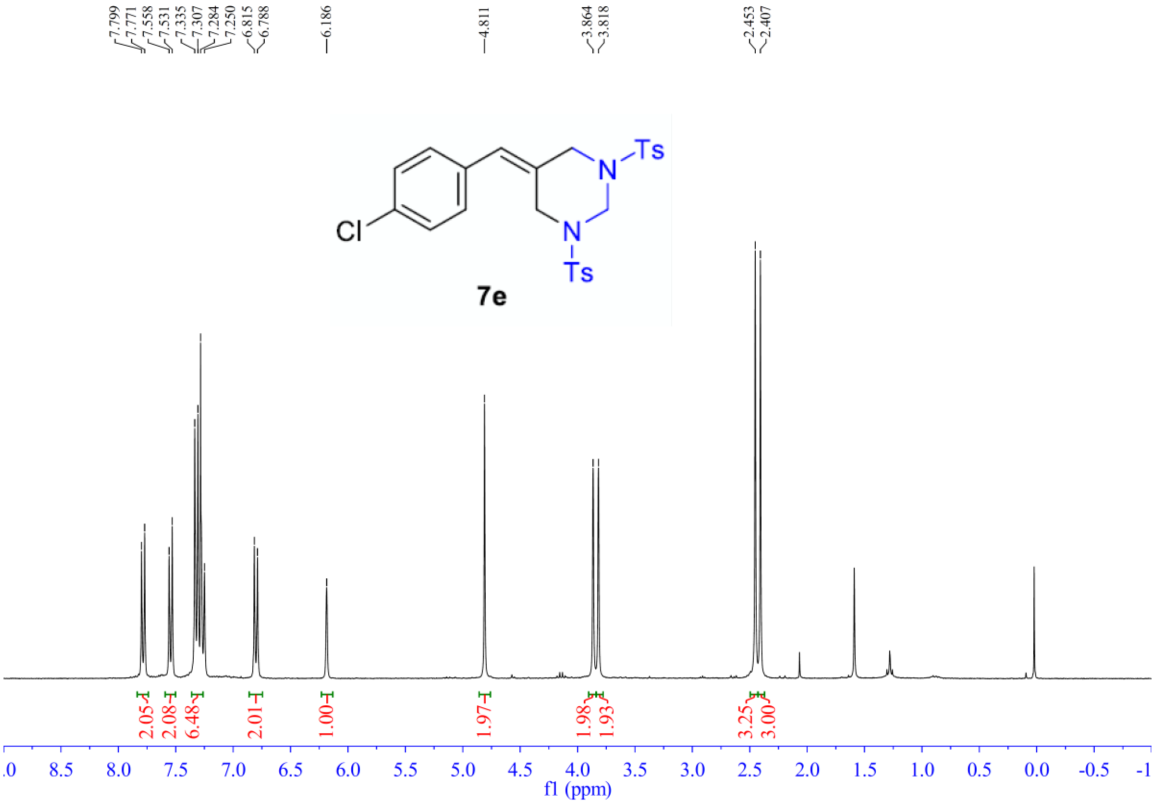

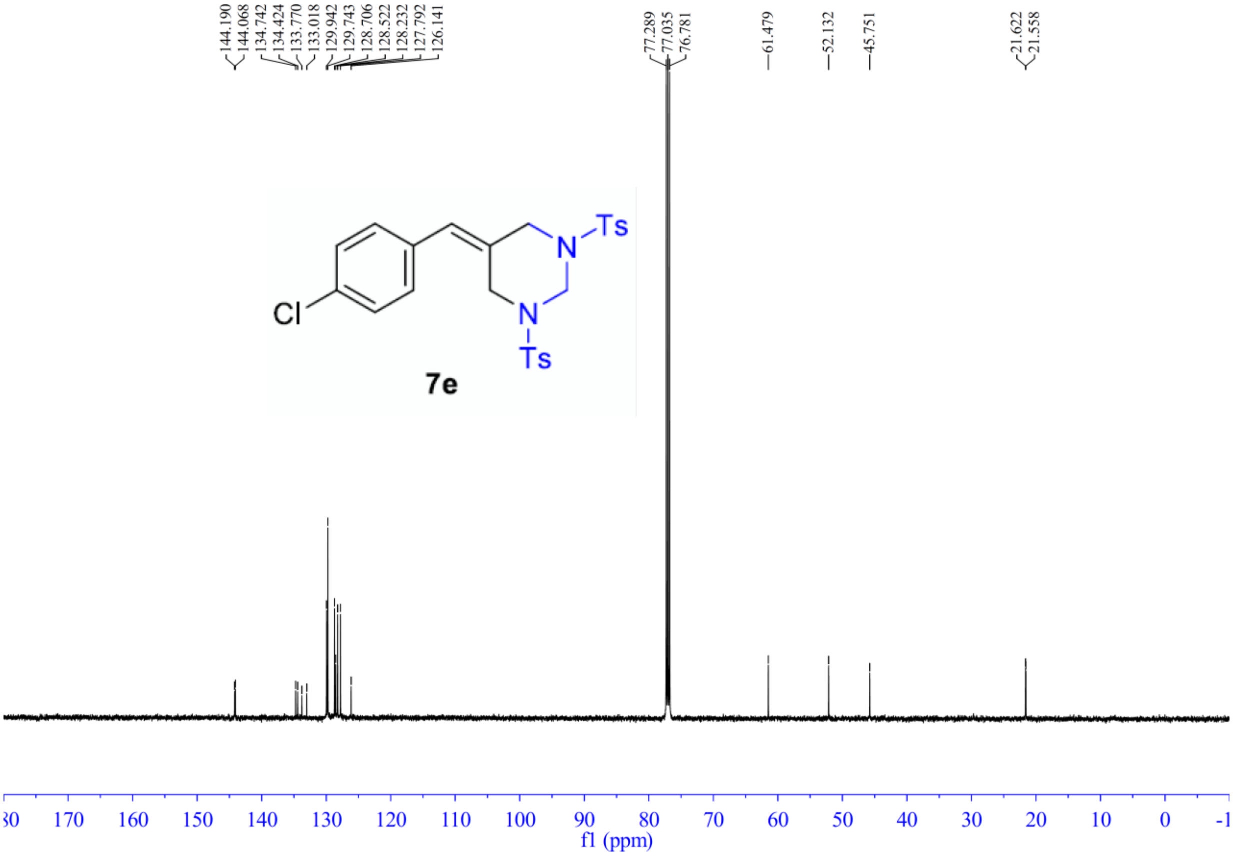

5-(4-bromobenzylidene)-1, 3-ditosylhexahydropyrimidine (5f):

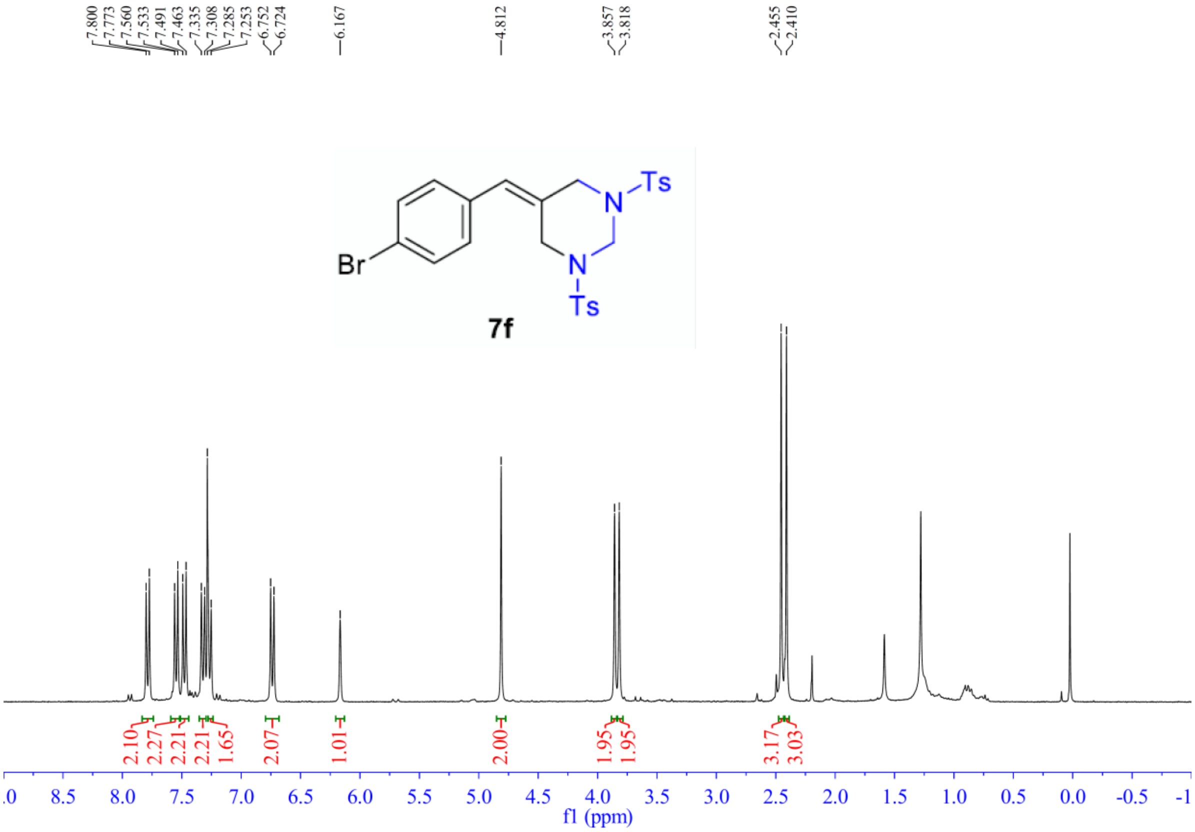

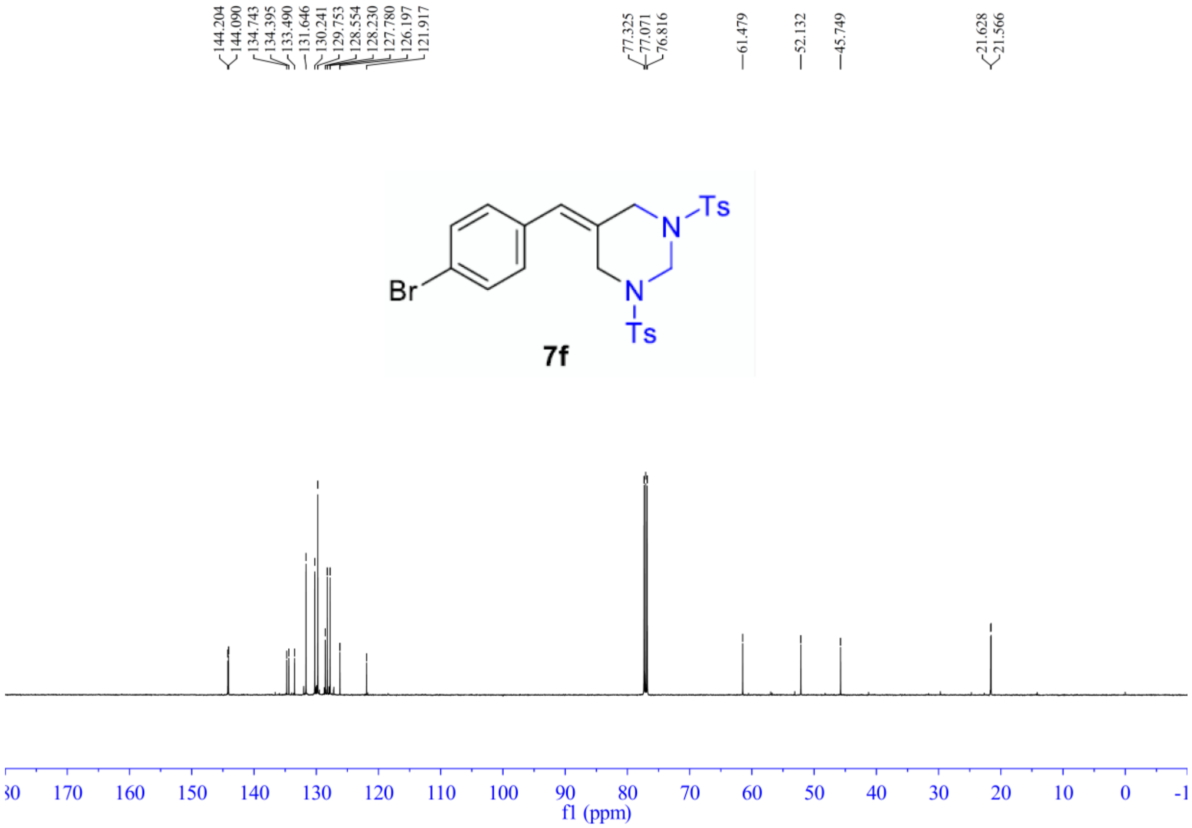

5-(2-chlorobenzylidene)-1, 3-ditosylhexahydropyrimidine (5g):

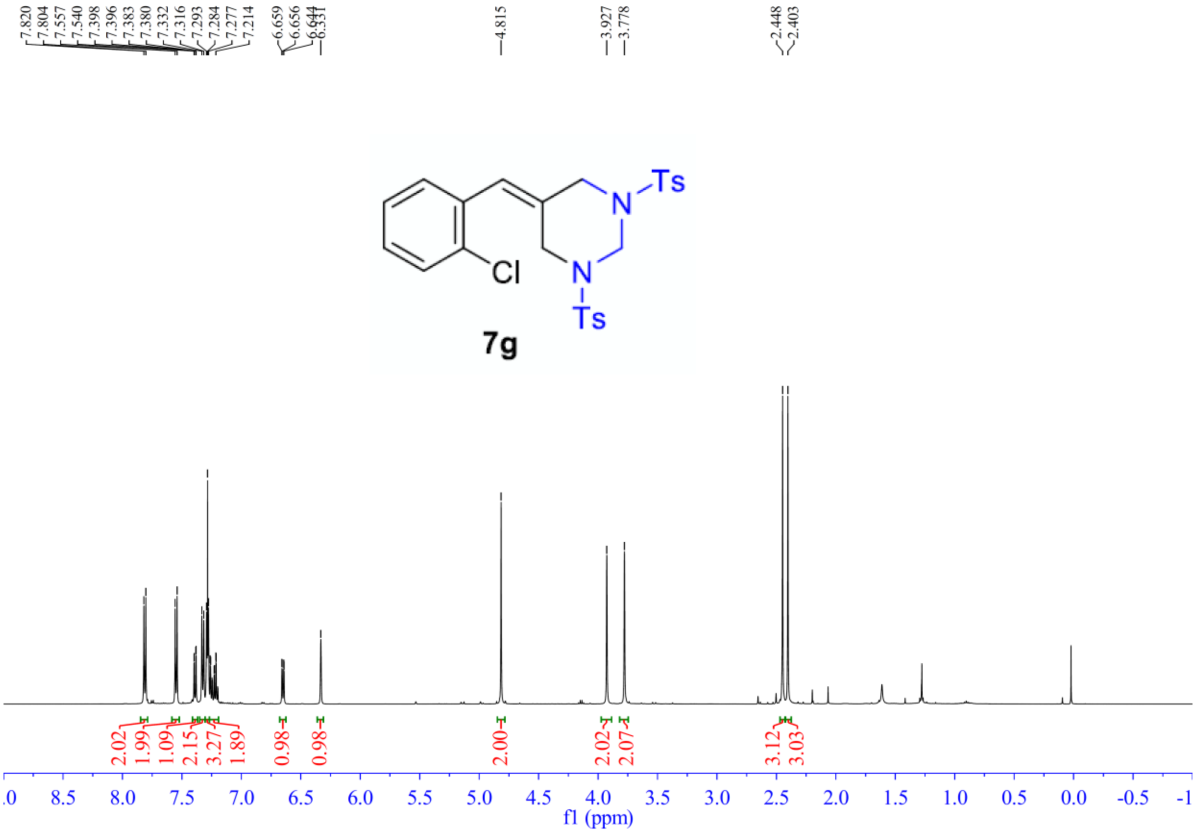

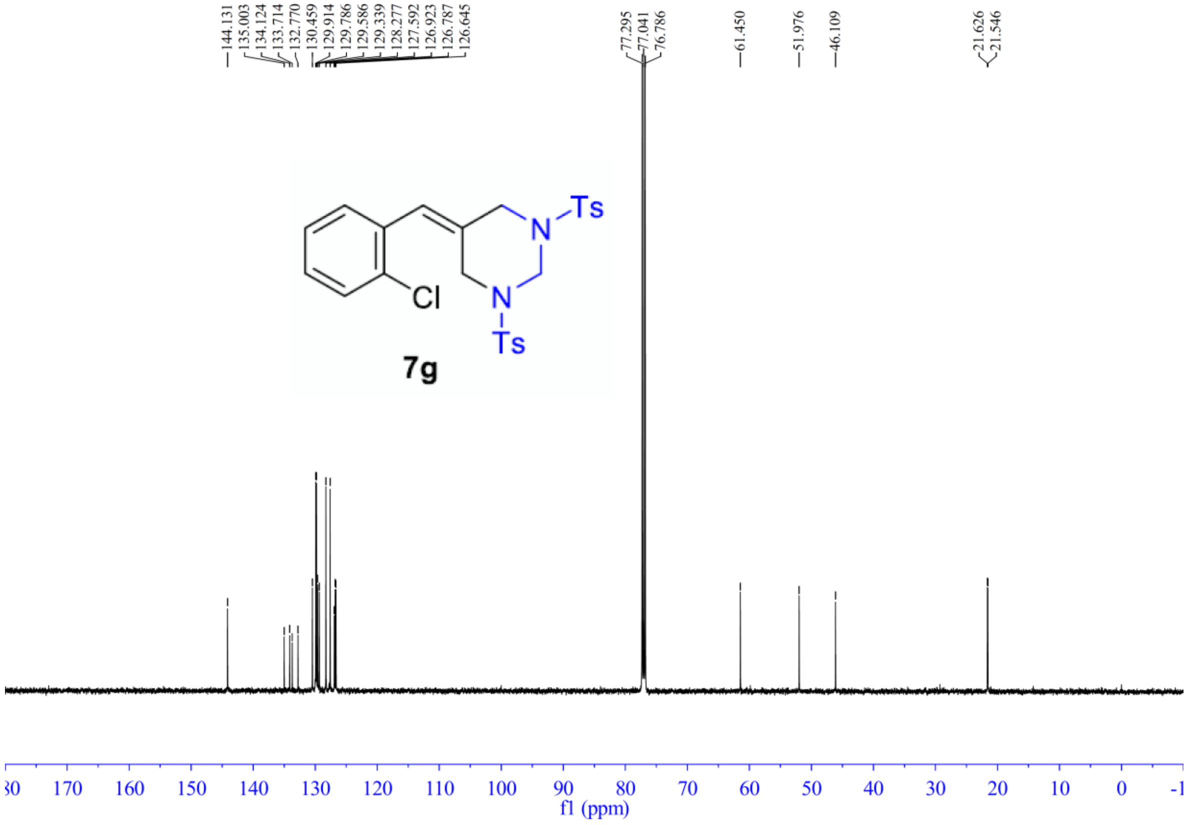

5-(1-phenylethylidene)-1, 3-ditosylhexahydropyrimidine (5h):

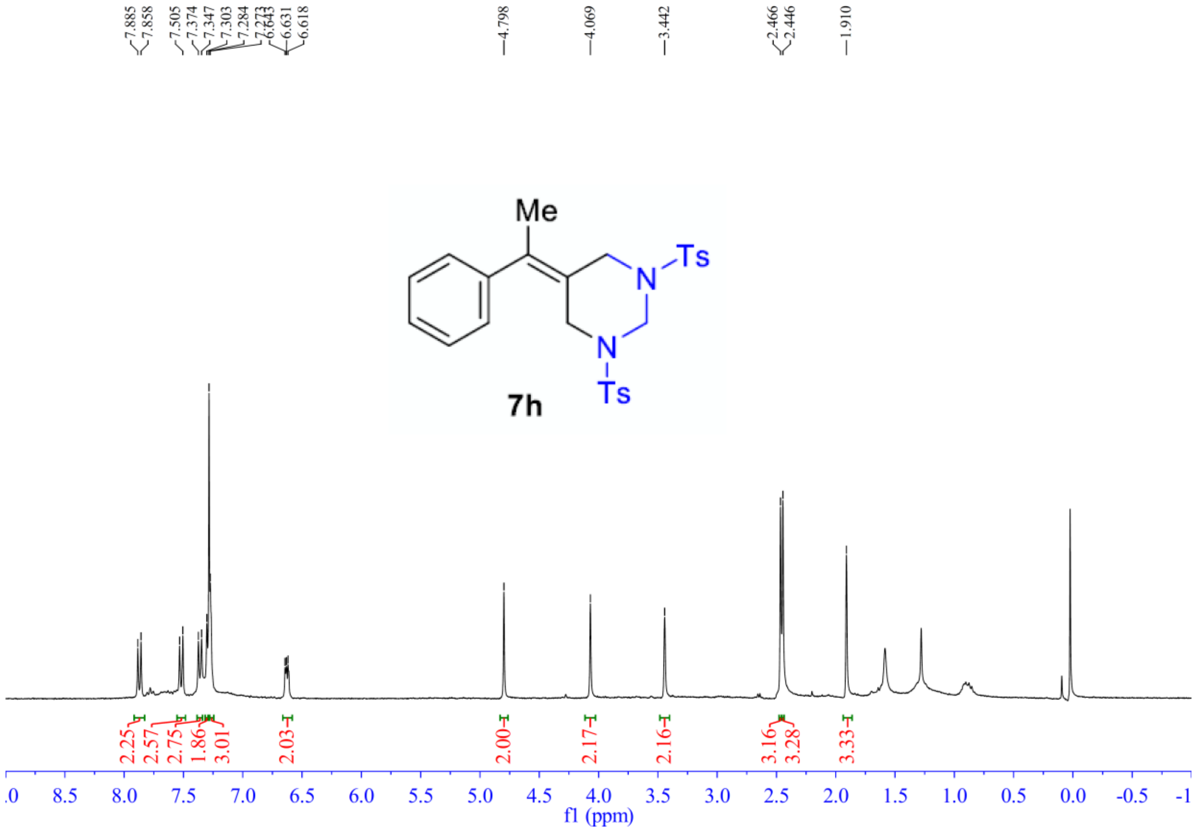

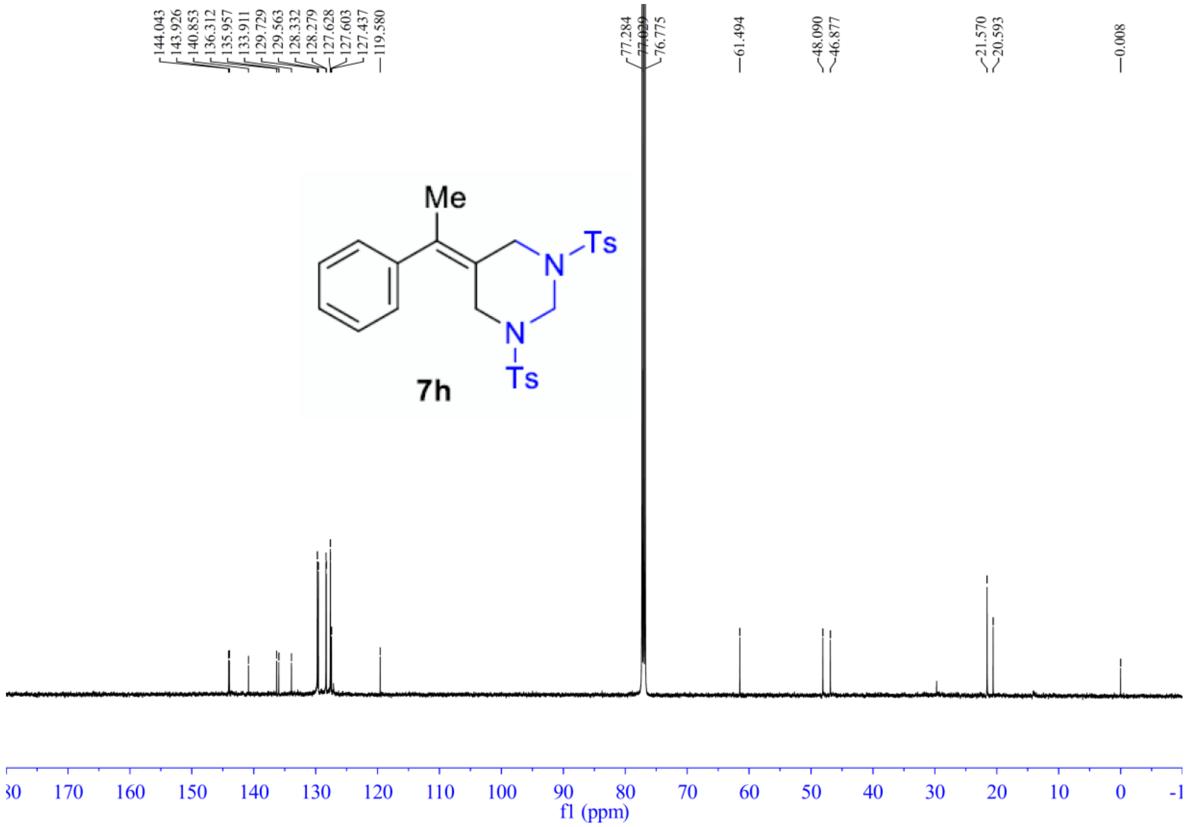

5-benzylidene-4-methyl-1, 3-ditosylhexahydropyrimidine (5i) :

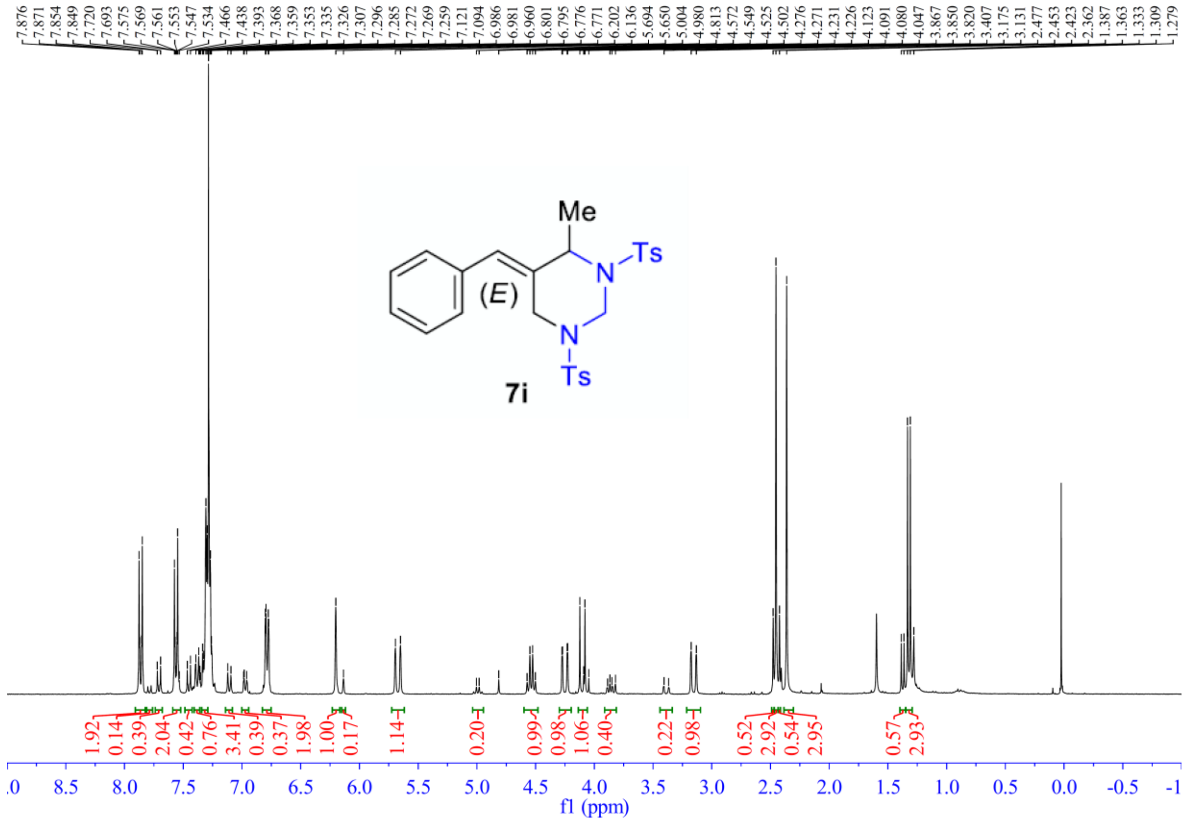

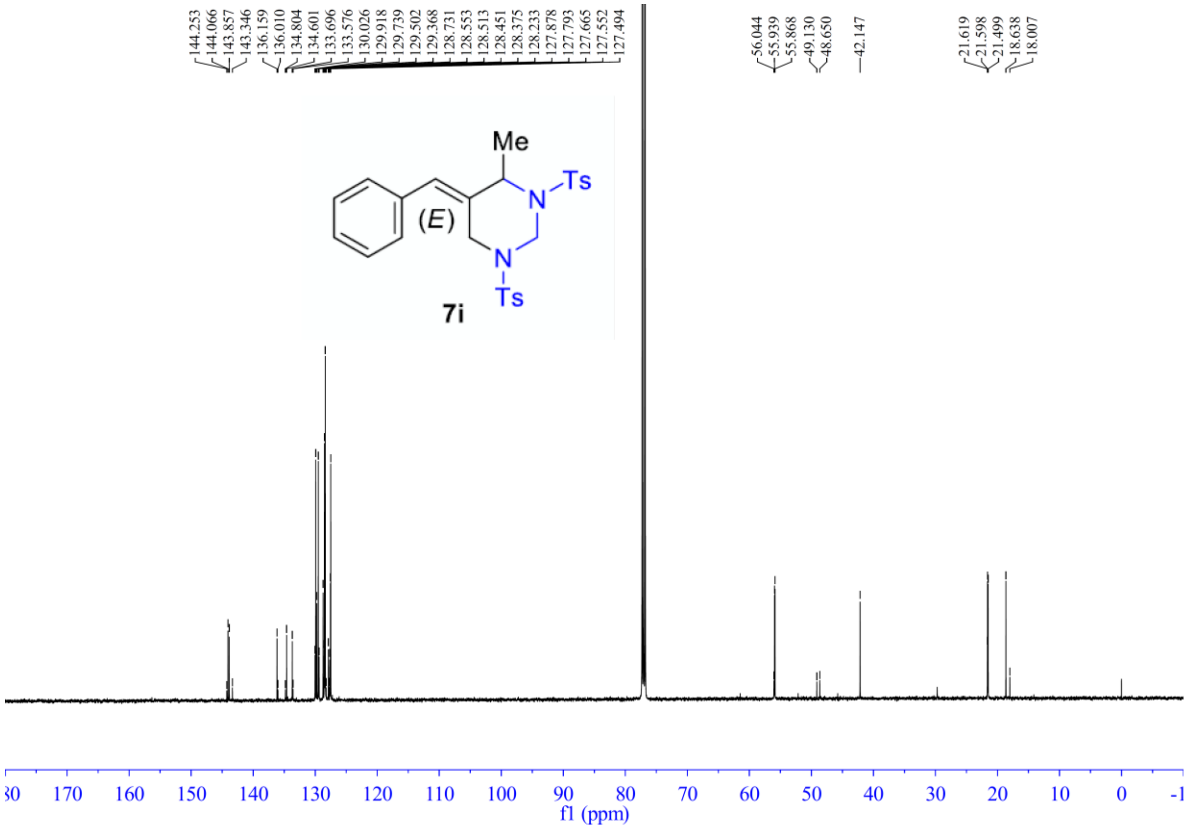

NOESY spectrum of a solution of **(*E*)- 5i** in $CDCl_3$:

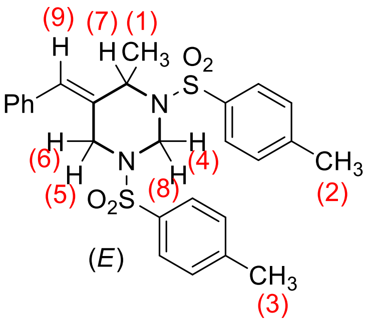

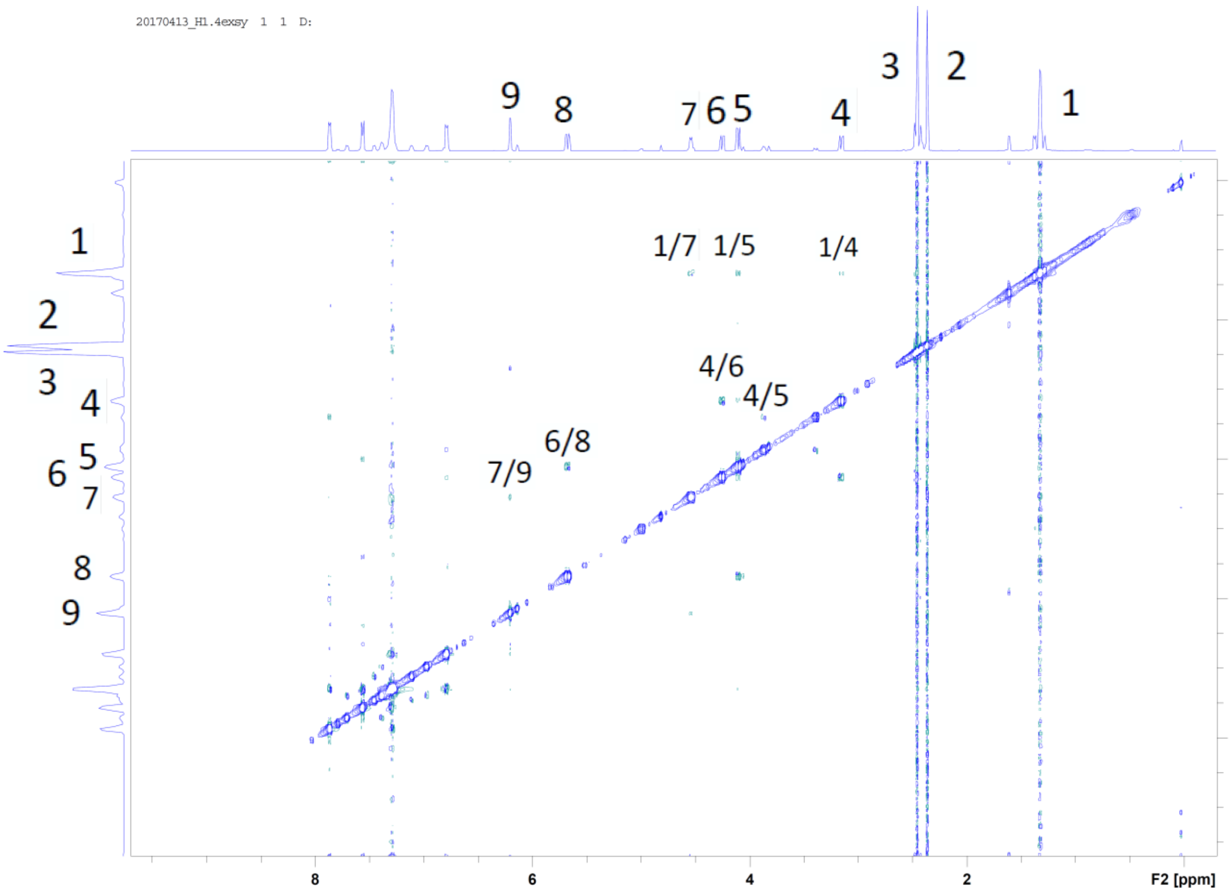

N, N'-(1-(4-tert-butylphenyl)propane-1, 3-diyl)bis(4-methylbenzenesulfonamide) (7d):

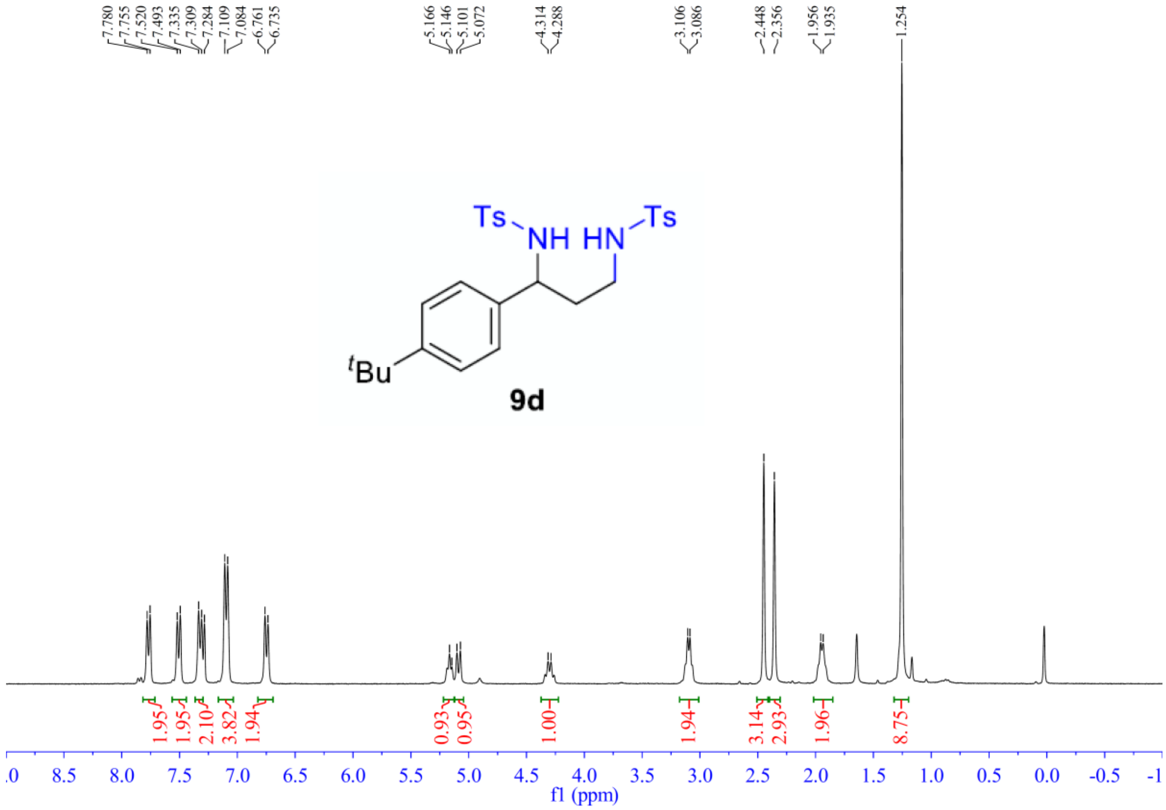

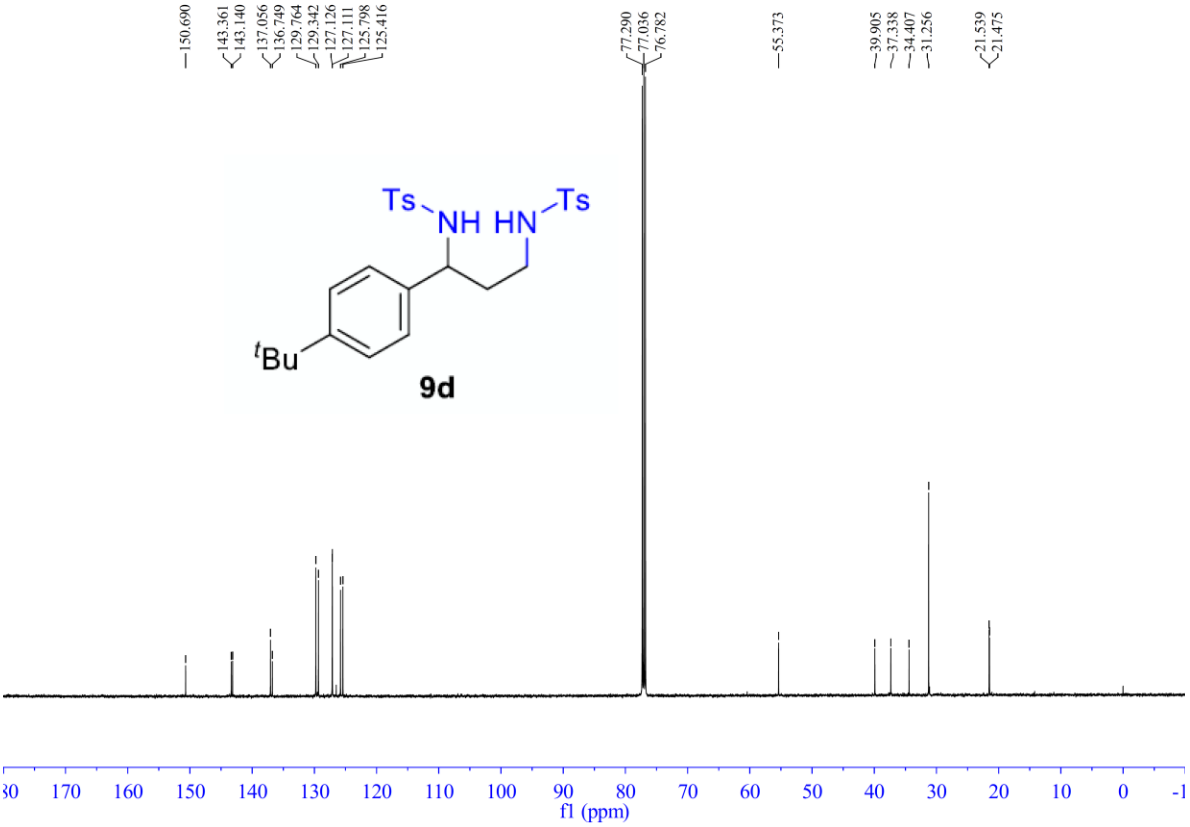

N, N'-(2-(4-chlorobenzylidene)propane-1,3-diyl)bis(4-methylbenzenesulfonamide) (7e)

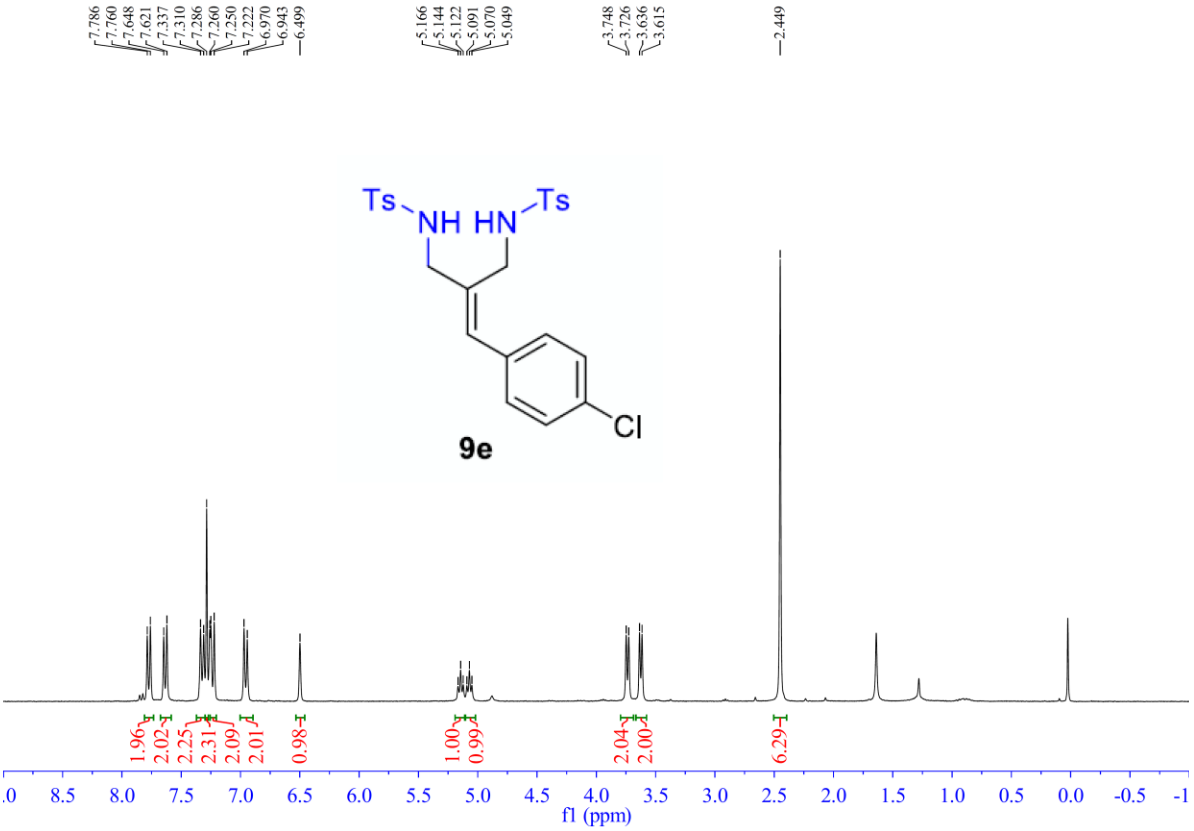

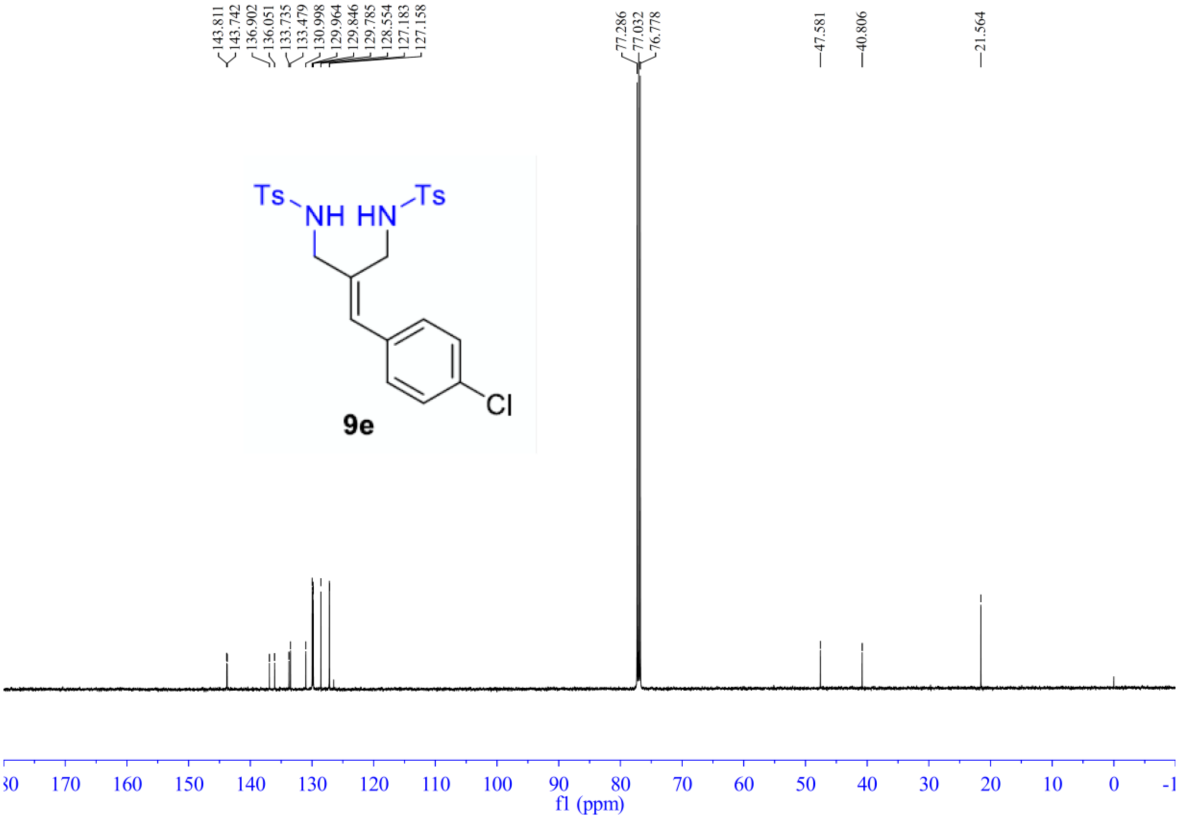

X-ray Crystallography

CRYSTAL SUMMARY (3c) (MSU0048)

Crystal data for $C_{25}H_{29}N_2O_4S_2$; M_r = 485.62; Triclinic; space group P-1; a = 9.5654(7) Å; b = 10.5731(8) Å; c = 13.0829(9) Å; α = 103.702(2)°; β = 107.842(2)°; γ = 96.750(2)°; V = 1197.53(15) Å^3; Z = 2; T = 100(2) K; λ(Mo-Kα) = 0.71073 Å; μ(Mo-Kα) = 0.257 mm^{-1}; d_{calc} = 1.347g.cm^{-3}; 26388 reflections collected; 4741 unique (R_{int} = 0.0346); giving R_1 = 0.0346, wR_2 = 0.0897 for 4741 data with [I>2σ(I)] and R_1 = 0.0461, wR_2 = 0.0936 for all 26388 data. Residual electron density (e^{-}.Å^{-3}) max/min: 0.31/-0.83.

An arbitrary sphere of data were collected on a clear and colorless block crystal, having approximate dimensions of 0.313 × 0.309 × 0.232 mm, on a Bruker D8 VENTURE PHOTON 100 CMOS diffractometer using a combination of ω- and φ-scans of 0.5° [1]. The crystal was kept at 100.0 K during data collection. Using Olex2 [2], the structure was solved with the ShelXT [3] structure solution program using direct methods and refined with the ShelXL [4] refinement package using least squares minimization. All non-hydrogen atoms were refined with anisotropic thermal displacement parameters. Unless otherwise noted, hydrogen atoms were included in calculated positions. Thermal parameters for the hydrogens were tied to the isotropic thermal parameter of the atom to which they are bonded (1.5 × for methyl, 1.2 × for all others).

CRYSTAL SUMMARY (5e) (MSU0049)

Crystal data for $C_{25}H_{25}ClN_2O_4S_2$; M_r = 517.04; Monoclinic; space group C2/c; a = 43.257(3) Å; b = 7.7831(5) Å; c = 14.4728(9) Å; α = 90°; β = 95.035(4)°; γ = 90°; V = 4853.8(5) Å^3; Z = 8; T = 100(2) K; λ(Mo-Kα) = 0.71073 Å; μ(Mo-Kα) = 0.365 mm^{-1}; d_{calc} = 1.415g.cm^{-3}; 55153 reflections collected; 4815 unique (R_{int} = 0.0379); giving R_1 = 0.0376, wR_2 = 0.0956 for 4815 data with [I>2σ(I)] and R_1 = 0.0439, wR_2 = 0.0995 for all 55153 data. Residual electron density (e^{-}.Å^{-3}) max/min: 0.68/-0.45.

An arbitrary sphere of data were collected on a clear and colorless plate crystal, having approximate dimensions of 0.445 × 0.328 × 0.06 mm, on a Bruker D8 VENTURE PHOTON 100 CMOS diffractometer using a combination of ω- and φ-scans of 0.5° [1]. The crystal was kept at 100.0 K during data collection. Using Olex2 [2], the structure was solved with the ShelXT [3] structure solution program using direct methods and refined with the ShelXL [4] refinement package using least squares minimization. All non-hydrogen atoms were refined with anisotropic thermal displacement parameters. Unless otherwise noted, hydrogen atoms were included in calculated positions. Thermal parameters for the hydrogens were tied to the isotropic thermal parameter of the atom to which they are bonded (1.5 × for methyl, 1.2 × for all others).

REFERENCES

[1] Bruker AXS. (2008). *APEX-2*. Bruker-Nonius AXS, Madison, Wisconsin, USA.

[2] Dolomanov, O.V., Bourhis, L.J., Gildea, R.J, Howard, J.A.K. & Puschmann, H. (2009), J. Appl. Cryst. 42, 339-341.

[3] Sheldrick, G.M. (2015). Acta Cryst. A71, 3-8.

[4] Sheldrick, G.M. (2015). Acta Cryst. C71, 3-8.
